# *Fusobacterium nucleatum*-reprogrammed adipocytes promote tumor cisplatin resistance through the CCL2-CCR2 axis in the necrotic metastatic neck nodes of head and neck carcinoma

**DOI:** 10.1186/s12964-025-02550-z

**Published:** 2025-11-24

**Authors:** Feiran Li, Xiaohui Yuan, Hui-Ching Lau, Yi Zhu, Hongli Gong, Huiying Huang, Chi-Yao Hsueh, Ming Zhang

**Affiliations:** 1 ENT institute and Department of Otorhinolaryngology, Eye & ENT Hospital, Fudan University, 83 Fen Yang Road, Shanghai, China; 2 Department of Radiation Oncology, Eye & ENT Hospital, Fudan University, 83 Fen Yang Road, Shanghai, China

**Keywords:** Adipocyte, Fusobacterium nucleatum, CCL2, Head and neck carcinoma, Cisplatin

## Abstract

**Background:**

Necrosis plays a pivotal role in the development of cisplatin resistance in metastatic neck lymph nodes of head and neck squamous cell carcinoma (HNSCC). However, the precise mechanisms underlying this association remain unclear.

**Methods:**

We employed qPCR and DNA in situ hybridization to detect *Fusobacterium nucleatum* (*F. nucleatum*) in postoperative tissue specimens from node-positive HNSCC patients. Transcriptomic sequencing was performed to analyze gene expression changes in adipocytes following *F. nucleatum* co-culture. RNA and protein expression alterations were validated via qPCR, Western blot, and ELISA. Additionally, subcutaneous xenograft tumor models were utilized for in vivo validation.

**Results:**

*F. nucleatum* was found to preferentially colonize necrotic neck lymph nodes in HNSCC and infiltrate adjacent adipocytes. In vitro, *F. nucleatum* induced the formation of cancer-associated adipocytes via autocrine C-C motif chemokine ligand 2 (CCL2), which stimulated lipolysis and enhanced free fatty acid release. Paracrine CCL2 further drove glutathione accumulation and cisplatin resistance in HNSCC by upregulating solute carrier family 1 member 5 (SLC1A5) and solute carrier family 7 member 11 (SLC7A11). Notably, C-C chemokine receptor type 2 (CCR2) antagonist, RS504393, effectively reversed these *F. nucleatum*-mediated pro-tumor effects. In vivo studies further confirmed the role of *F. nucleatum*-reprogrammed adipocytes and the therapeutic potential of RS504393.

**Conclusion:**

This study is the first to elucidate the crucial involvement of *F. nucleatum* in shaping cancer-associated adipocytes within the HNSCC microenvironment. *F. nucleatum*-reprogrammed adipocytes enhance cisplatin resistance via the CCL2-CCR2 axis, offering new therapeutic avenues to overcome chemotherapy resistance in necrotic neck lymph nodes.

**Graphical Abstract:**

*Fusobacterium nucleatum* (*F. nucleatum*) drives the transformation of adipocytes into a cancer-associated phenotype via autocrine CCL2 signaling. This process increases free fatty acid release to fuel tumor progression by enhancing lipolysis through the activation of CREB/HSL phosphorylation. Additionally, paracrine CCL2 from *F. nucleatum*-reprogrammed adipocytes upregulates SLC1A5 and SLC7A11 in head and neck squamous cell carcinoma (HNSCC), leading to glutathione (GSH) accumulation and cisplatin resistance. Pharmacological inhibition of CCR2 with RS504393 attenuates both cancer-associated adipocyte formation and cisplatin resistance, highlighting the therapeutic potential of targeting the CCL2-CCR2 axis in HNSCC.

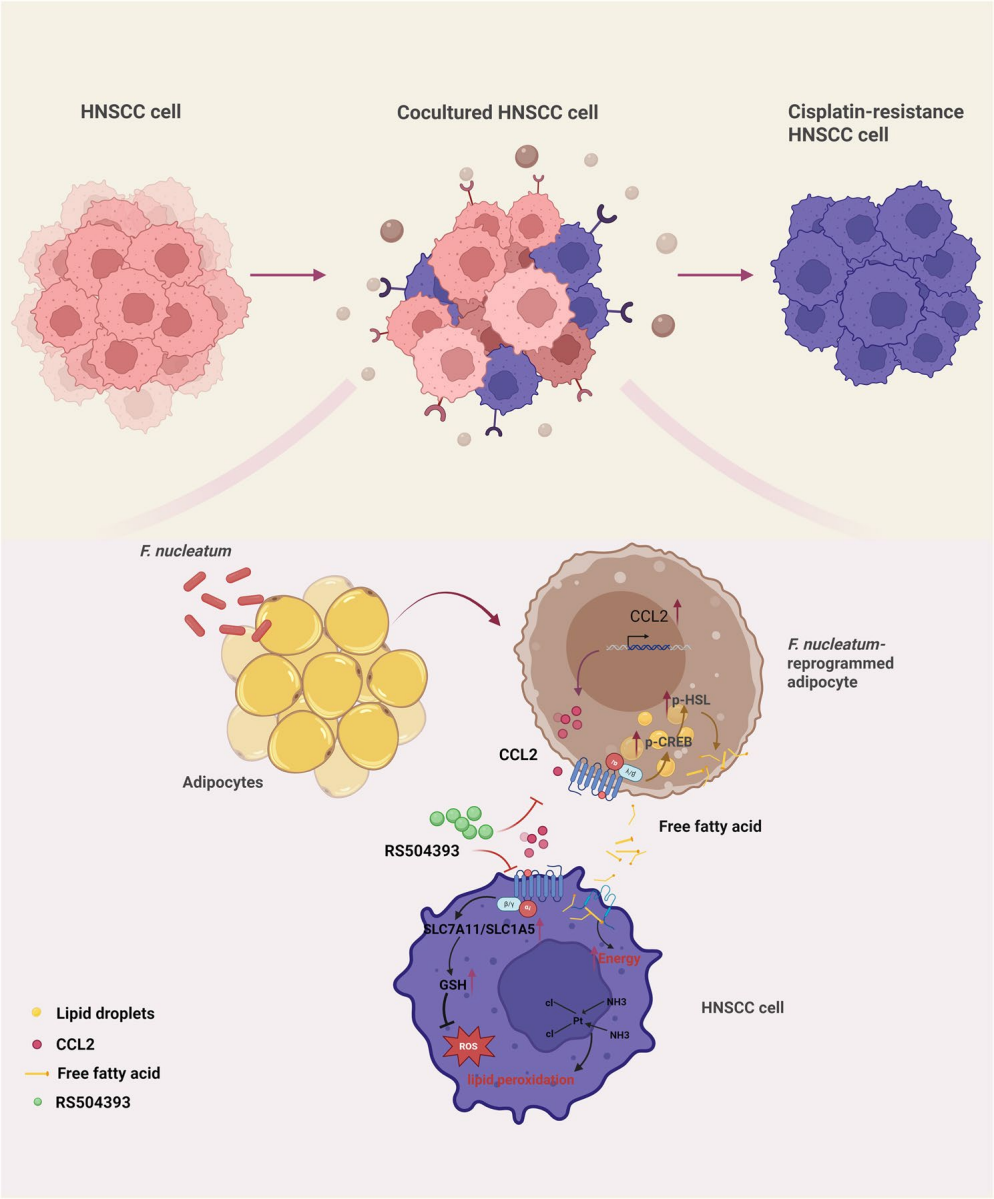

**Supplementary Information:**

The online version contains supplementary material available at 10.1186/s12964-025-02550-z.

## Introduction

Cisplatin-based chemotherapy resistance in cervical lymph node metastases remains a critical determinant of poor prognosis in patients with head and neck squamous cell carcinoma (HNSCC) [1]. In recent years, induction chemotherapy-guided treatment has gradually become an important therapeutic approach for HNSCC. However, unlike primary tumors, cervical lymph node metastases frequently exhibit poorer treatment response [2], and in some cases, recurrence is confined solely to metastatic nodes [3]. In our previous study, we systematically investigated factors influencing chemosensitivity in metastatic neck nodes and identified tumor necrosis as a critical determinant of treatment resistance [4]. Nevertheless, the underlying mechanisms by which necrotic lymph nodes develop chemotherapy resistance remained poorly understood.

Emerging clinical studies have consistently associated cancer-associated adipocytes (CAAs) infiltration with unfavorable outcomes and reduced chemosensitivity across various malignancies [5–8]. As key stromal components of the tumor microenvironment, CAAs play a pivotal role in cancer biology. Functioning as metabolic regulators and paracrine signaling hubs, CAAs sustain tumor growth via dual mechanisms: nutritional support through lipid transfer and pleiotropic signaling via cytokine secretion [8–10]. However, the potential contribution of these adipocytes to HNSCC progression and cisplatin resistance of metastatic neck nodes has not been fully investigated.

As a gateway between the external environment and the lower respiratory tract, the head and neck region maintains a complex ecosystem of commensal bacteria [11]. Our previous study demonstrated that *Fusobacterium* is significantly enriched in HNSCC specimens [12–15]. Notably, *Fusobacterium nucleatum* (*F. nucleatum*), a key species within *Fusobacterium*, has been strongly correlated with poor clinical outcomes in HNSCC patients [16, 17]. Despite these advances, the potential involvement of *F. nucleatum* in adipocyte transformation and chemoresistance development within necrotic metastatic lymph nodes remains unexplored.

To address these gaps, this study investigates the mechanistic role of *F. nucleatum*-reprogrammed adipocytes in promoting cisplatin resistance within necrotic metastatic cervical lymph nodes of HNSCC patients.

## Materials and methods

### Patient specimen collection

This study was approved by *the Institutional Review Board of Eye & ENT Hospital*,* Fudan University* (Approval No. 2023177; Clinical-trial registration number, ChiCTR2400080021) with written informed consent obtained from all participants. We enrolled 24 histologically confirmed HNSCC patients who underwent surgical resection of either primary tumors with metastatic cervical lymph nodes or residual metastatic neck nodes alone. All patients were pathologically confirmed to have cervical lymph node metastasis. Patients with distant metastases or synchronous second primary malignancies were excluded. Patients’ basic characteristics were shown in the Supplemental Table 1. Intraoperative tumor surface swabs were collected aseptically and stored at −80 °C. Resected tissues were formalin-fixed and paraffin-embedded for subsequent analyses.

### Xenograft tumor model

This study was conducted with approval from *the animal ethics committee of Fudan University* (IACUC-DWZX-2024-013). The methods/medication was administered to minimize suffering during the experiments, the methods of sacrifice, sample collection, anesthesia, housing, sustenance, and storage conditions. The sample size of five mice per group was determined based on previous similar studies, which established that this number provides sufficient statistical power [5]. An independent researcher, not involved in subsequent procedures, randomly allocated the mice to the control and treatment groups. While the personnel administering injections could not be blinded due to the different dosing regimens between groups, all data collection and outcome assessments performed by investigators blinded to the group allocation.

Three-to-four-week-old male BALB/c-Nude mice (GemPharmatech, China) were housed under specific pathogen-free (SPF) conditions throughout the study period. To establish xenograft models, 3 × 10^6^ per site Fadu cells were subcutaneously injected into the left back or right groin of nude mice (five mice per group). Subcutaneous tumor growth was monitored at three-day intervals, with tumor volume calculated as (length × width²)/2. For the establishment of an *F. nucleatum* infection model, *F. nucleatum* was injected intraperitoneally at 0.1 CFU/g every 3 days since subcutaneous tumor formation. Upon achieving a tumor volume of 100 mm³, cisplatin was injected intraperitoneally at 4 mg/kg as previously described [18], with or without RS504393 injected intraperitoneally at 6 mg/kg as described earlier [19], on Days 0, 7, and 14. Three days after the last drug administration, the mice were sacrificed, and the tumor tissues were collected. Tumor volume and tumor mass were recorded.

### In situ hybridization (ISH) and multiplex fluorescence staining

For ISH, tissue sections underwent microwave retrieval, proteinase K digestion, permeabilization, peroxidase blocking, and PBS washes. DIG-labeled *F. nucleatum* probes [20] were hybridized (42 °C, overnight) (probe sequences are provided in Supplemental Table 2), followed by SSC washes. Detection used anti-DIG-biotin, HRP-streptavidin, and DAB/hematoxylin staining.

For multiplex fluorescence staining, post-hybridization, FITC/CY3-TSA was applied (37°C, light-protected). After antigen retrieval, primary antibody (4°C, overnight, shown in Supplemental Table 3) and HRP-secondary antibody were incubated, followed by CY3/FITC-TSA and DAPI counterstaining (RT, light-protected).

### Cell lines and culture

3T3-L1 cells were obtained from the Cell Bank of the Shanghai Academy of Chinese Sciences. It was recently authenticated by STR testing and tested without mycoplasma contamination. 3T3-L1 were cultured in DMEM (Gibco, USA) supplemented with 10% newborn calf serum (Every Green, China). Adipocyte differentiation was induced following the established protocol of Furukawa et al. [21]. For conditioned medium (CM) preparation, mature adipocytes were PBS-washed and serum-starved for 24 h. Supernatants were then collected, centrifuged (3,000 rpm, 20 min), and stored at −80 °C. Hypoxia condition was established by hypoxia incubator chamber (Thermo Scientific, USA).

HNSCC cell lines (Fadu, AMC-HN-8) were obtained from Eye & ENT Hospital, Fudan University. They were recently authenticated by STR testing and tested without mycoplasma contamination. Fadu cells were maintained in high-glucose DMEM (Gibco, USA) supplemented with 10% fetal bovine serum (BI, USA), while AMC-HN-8 cells were cultured in 1640 medium (Gibco, USA) containing 10% fetal bovine serum (BI, USA), at 37 °C with 5% CO₂.

### Cell proliferation, drug sensitivity, apoptosis, and necrosis assays

Cell viability was assessed via CCK-8 assay. Cells (1,000/well in 96-well plates, 100 µL medium) adhered overnight, then treated with *F. nucleatum* and monitored for 5 days. 10 µL CCK-8 was added, incubated (37 °C, 1 h), and OD450 measured. EdU assay was performed using BeyoClick™ EdU Kit (AF594). For drug sensitivity, cells (8,000/well) were treated with cisplatin/RS504393 (gradient conc.), incubated for 48 h, and viability was measured via CCK-8 to calculate IC50. Apoptosis/necrosis was detected using Annexin V-FITC/PI staining (Beyotime Kit), analyzed by flow cytometry/fluorescence microscopy.

### Intracellular lipid droplet and extracellular free fatty acid (FFA) detection

Lipid droplets were visualized using both Oil Red O (Oricell, China) and BODIPY 493/503 (MCE, USA). For Oil Red O staining, following 4% PFA fixation, cells were stained with freshly prepared Oil Red O solution (3:2 dilution in distilled water, centrifuged at 250×g) for 15 min at RT. For BODIPY 493/503 staining, samples were incubated with 2 µM BODIPY 493/503 (1 ml/well) for 30 min at RT. The content of supernatant free fatty acid was measured using the Amplex Red Free Fatty Acid Assay Kit (Beyotime, China) according to the manufacturer’s recommended protocol.

### RNA and DNA extraction and real-time quantitative PCR

Total RNA was isolated from collected cells using TRIzol reagent, followed by cDNA synthesis with a reverse transcription kit (Accurate Biotechnology, China). qPCR analysis was performed using SYBR Green Mix (Accurate Biotechnology, China) on a real-time PCR instrument (Applied Biosystems, USA). *F. nucleatum* DNA was extracted from swab of primary sites using QIAamp DNA Host-Free Microbiome Kit (QIAGEN, Germany) and qPCR was performed using Supplemental Microbial qPCR Mastermix (ROX) (QIAGEN, Germany). Primer sequences (designed by Primer Blast) are provided in Supplemental Tables 4, and relative gene expression was determined using the ΔΔCt method or -ΔCt method.

### Western blot and ELISA quantitation

For western blot, protein extracts (RIPA buffer) were denatured, separated by SDS-PAGE, and transferred to PVDF membranes. After blocking (5% milk), membranes were incubated with primary (4 °C, overnight, shown in Supplemental Table 3) and HRP-secondary antibodies (RT, 1 h), then detected via ECL.

The ELISA quantitation of CCL2 was performed using Mouse MCP-1/CCL2 ELISA Kit (Boster Biological Technology, China). Samples and standards were incubated (37 °C, 90 min) in antibody-coated wells, followed by detection antibody (37 °C, 1 h), ABC solution (37 °C, 30 min), and TMB substrate. Absorbance was measured at 450 nm after stopping the reaction.

### Transcriptome sequencing

Total RNA was isolated using TRIzol, with mRNA enriched via oligo-dT-attached magnetic beads. The RNA-seq library was constructed using the Illumina^®^ Stranded mRNA Prep, Ligation protocol (Illumina, San Diego, CA) with 1 µg of total RNA input following the manufacturer’s specifications. Sequencing data were analyzed with DESeq2 software (thresholds: |log2FC|≥1, *p* < 0.05) and processed on the Majorbio Cloud Platform (https://cloud.majorbio.com/page/tools/*).*

### GSH analysis and lipid peroxidation assay

The content of GSH was measured using the GSH and GSSG Assay Kit (Beyotime, China) according to the manufacturer’s recommended protocol. BODIPY 581/591 C11 probe (Beyotime, China) was used for lipid peroxidation. BODIPY 581/591 C11 probe was diluted in PBS at a ratio of 1:1000 to a final concentration of 2 µM and then added to the culture plate for incubation at 37 °C for 30 min. After incubation, cells were observed under a fluorescence microscope.

### Statistical analysis

Continuous variables were analyzed using Student’s t-test (two groups) or one-way ANOVA (multiple groups), while two-way ANOVA with coefficient calculation was applied for dual-variable comparisons. Categorical data were assessed by the chi-square test. ImageJ was used to quantify fluorescence/Oil Red O-positive areas. All experiments were performed with at least three independent biological replicates. Statistical analysis was performed via GraphPad Prism 9 (GraphPad Software, USA). A two-sided *P* < 0.05 was considered statistically significant.

## Results

### *F. nucleatum* invades adipocytes in necrotic metastatic neck nodes of HNSCC

**Fig. 1:**
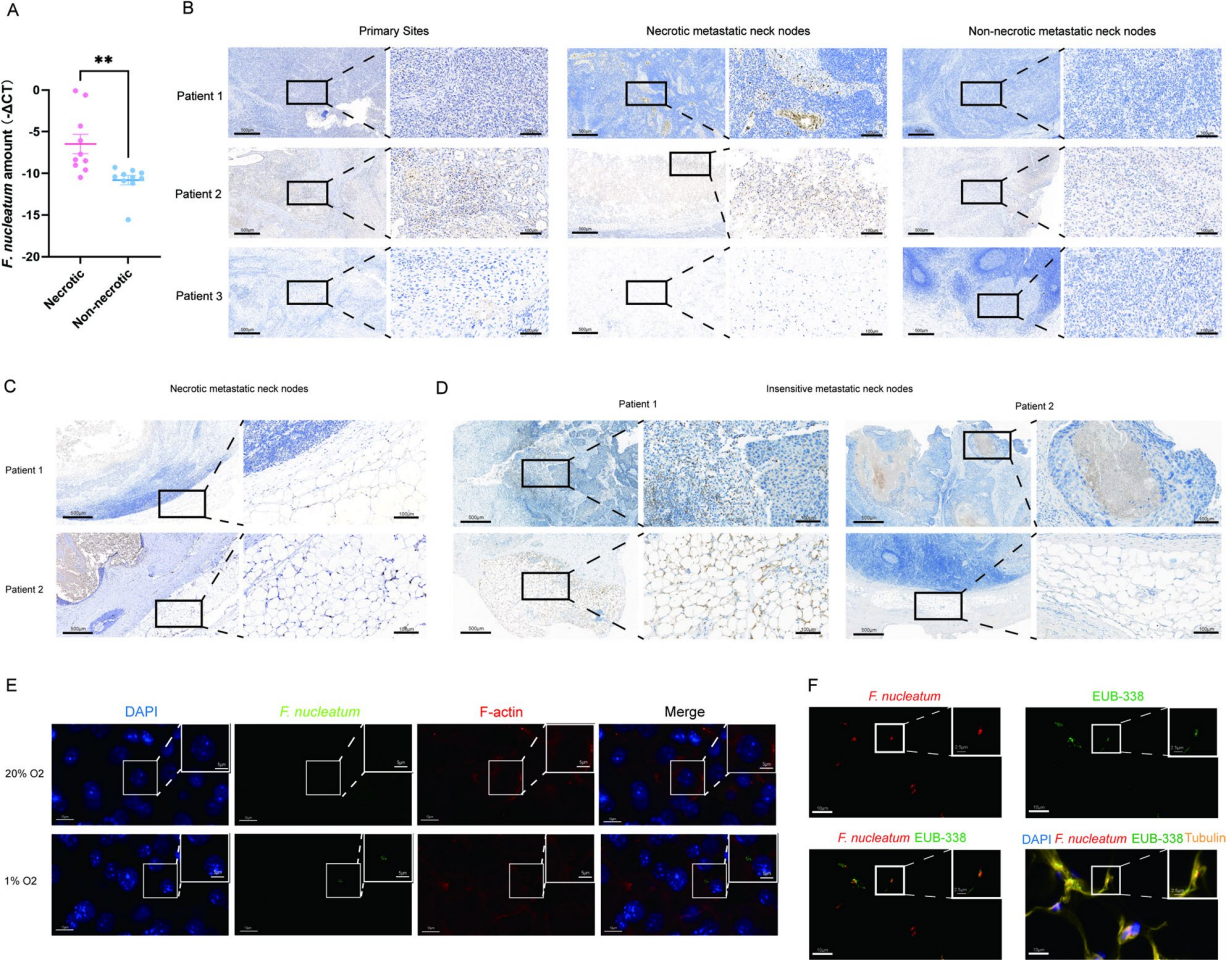
*F. nucleatum* invades adipocytes in necrotic metastatic neck nodes of HNSCC. **A** The amount of *F. nucleatum* in the tumor surface swabs from patients with necrotic and non-necrotic metastatic neck nodes (*n* = 20). **B** In situ hybridization image of *F. nucleatum* in primary sites, necrotic metastatic neck nodes, non-necrotic metastatic neck nodes of HNSCC patients (*n* = 3). **C** In situ hybridization image of *F. nucleatum* invades the surrounding adipocytes (*n* = 2). **D** In situ hybridization image of *F. nucleatum* in residual neck nodes after induction chemotherapy (*n* = 2). **E** Fluorescence in situ hybridization image of *F. nucleatum* coculturing with adipocytes in normal or hypoxic conditions (*n* = 3). **F** Fluorescence in situ hybridization image of *F. nucleatum* in the groin adipose tissue of nude mice

To explore the potential role of *F. nucleatum* in cisplatin resistance, we conducted a comparative analysis of clinical specimens from HNSCC patients. Intriguingly, quantitative analysis revealed a significant increase in *F. nucleatum* load from tumor surface swabs in patients with necrotic metastatic neck nodes compared to those with non-necrotic metastases (*P* = 0.0050) (Fig. 1A), implying a possible association between *F. nucleatum* and nodal metastasis necrosis. Further investigation using in situ hybridization (ISH) demonstrated markedly elevated *F. nucleatum* abundance in both necrotic metastatic lymph nodes and primary tumor tissues relative to their non-necrotic counterparts (Fig. 1B). These findings suggested that *F. nucleatum* exhibited a tropism for necrotic tissue microenvironments, with primary tumors potentially serving as reservoirs for bacterial dissemination to metastatic sites. Notably, *F. nucleatum* was detected not only within necrotic regions but also in adjacent adipose tissue, suggesting possible adipocyte infiltration and infection in the necrotic metastatic neck nodes (Fig. 1C).

To directly assess the association between *F. nucleatum* and therapeutic resistance, we analyzed residual metastatic lymph nodes from patients exhibiting poor response to induction chemotherapy. ISH confirmed the presence of *F. nucleatum* in these chemotherapy-resistant nodes. Moreover, bacterial infiltration into surrounding adipose tissue raised the possibility that *F. nucleatum*-modified adipocytes contribute to treatment failure (Fig. 1D).

Subsequent in vitro experiments demonstrated that hypoxic conditions, mimicking the necrotic microenvironment, significantly enhanced *F. nucleatum* infection of adipocytes (Fig. 1E). Vivo studies also confirmed *F. nucleatum*’s ability to colonize adipose tissue following intraperitoneal injection in murine models (Fig. 1F). Therefore, *F. nucleatum* invaded adipocytes within necrotic metastatic lymph nodes, which may contribute to the development of cisplatin resistance in HNSCC.

### *F. nucleatum*-infected adipocytes acquire cancer-promoting traits

**Fig. 2:**
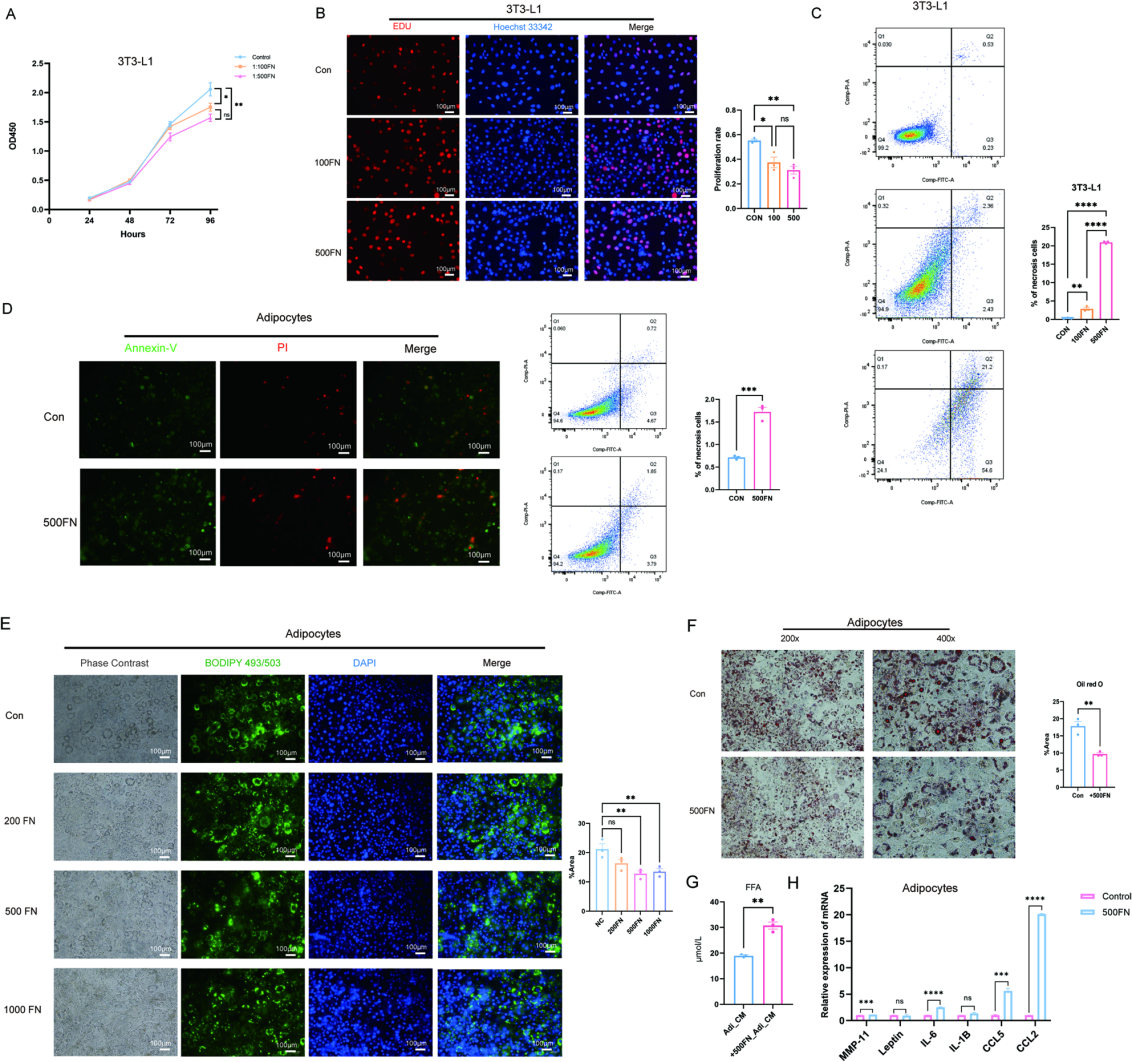
*F. nucleatum*-infected adipocytes acquire cancer-promoting traits. **A** CCK-8 assay of 3T3-LI cells with different concentrations of *F. nucleatum* (*n* = 3). **B** EDU image of 3T3-LI cells with different concentrations of *F. nucleatum* (*n* = 3). **C** Annexin/PI stain of 3T3-L1 after cocultured with *F. nucleatum* (*n* = 3). **D** Annexin/PI stain of adipocytes after coculturing with *F. nucleatum* (*n* = 3). **E** BODIPY 493/503 staining of lipid droplets in adipocytes after coculturing with *F. nucleatum* (*n* = 3). **F** Oil Red O staining of lipid droplets in adipocytes after coculturing with *F. nucleatum* (*n* = 3). **G** The content of free fatty acid in the supernatant of adipocytes after coculturing with *F. nucleatum* (*n* = 3). **H** Relative expression level of genes regarding pro-tumorigenic cytokines after coculturing with *F. nucleatum* (*n* = 3). FN: *Fusobacterium nucleatum*. FFA: free fatty acid. AdiCM: adipocyte supernatant. Data are presented as means ± SEM. *P < 0.05.* *P* < 0.05,‌** *P* < 0.01, ‌*** *P* < 0.001, **** *P* < 0.0001, ns: no statistical significance

When preadipocytes and mature adipocytes were co-cultured with *F. nucleatum* at different bacterial-to-host cell ratios, we observed that the infection significantly inhibited preadipocyte growth (*P* = 0.0011) (Fig. 2A and B) and induced necrosis in both preadipocytes and mature adipocytes (Fig. 2C and D). Moreover, *F. nucleatum* infection triggered a transformation in adipocytes, manifesting as reduced intracellular lipid droplets (*P* = 0.0056) (Fig. 2E and F), accompanied by increased release of free fatty acids into the culture supernatant (*P* = 0.0012) (Fig. 2G). Concurrently, we observed marked upregulation of multiple pro-tumor factors, including MMP-11, IL-6, IL-1β, CCL5, and CCL2 (Fig. 2H). These findings demonstrated that *F. nucleatum* reprogrammed adipocytes into a cancer-associated phenotype marked by enhanced lipolytic activity and sustained secretion of tumor-promoting factors.

### *F. nucleatum*-reprogrammed adipocytes accelerate the cisplatin resistance of HNSCC

**Fig. 3:**
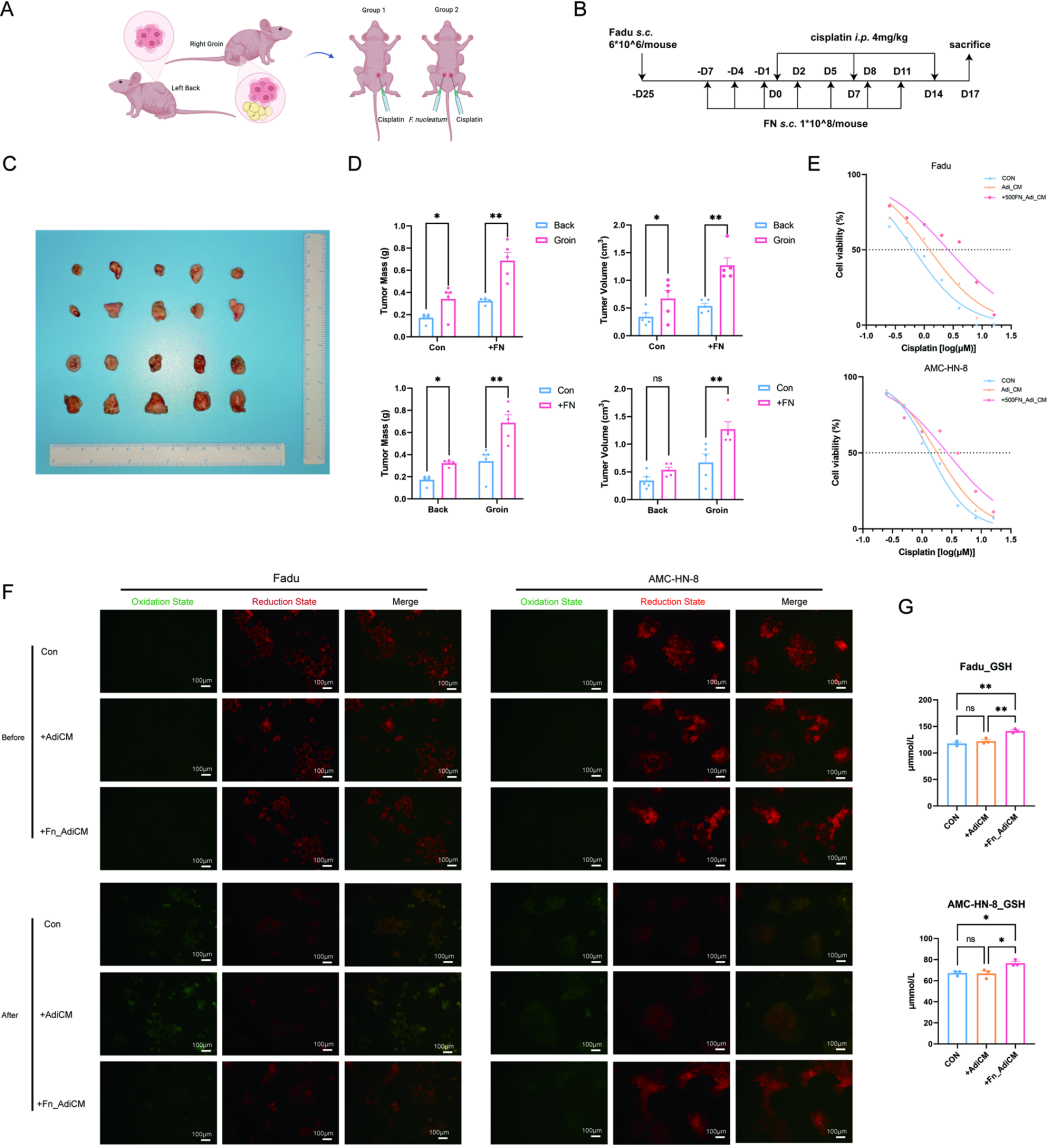
*F. nucleatum*-reprogrammed adipocytes accelerate the cisplatin resistance of HNSCC. **A**-**B** Schematic of BALB/c-Nude mouse modelling protocol. **C** Photos of left back and right groin tumors obtained from nude mice (*n* = 5). From top to bottom: dorsal tumor (Control), inguinal tumor (Control), dorsal tumor (FN-treated), and inguinal tumor (FN-treated). **D** The analysis of volume and mass of the obtained tumors (*n* = 5). **E** The change of cisplatin IC50 in Fadu and AMC-HN-8 cells after adding normal culture medium (0.6580, 95%CI 0.5587 to 0.7663 and 1.353, 95%CI 1.250 to 1.463), supernatant of adipocyte (1.226, 95%CI 0.9716 to 1.526 and 1.791, 95%CI 1.651 to 1.942) and *F. nucleatum*-cocultured adipocytes (2.580, 95%CI 1.981 to 3.350 and 2.667, 95%CI 2.205 to 3.226) (*n* = 3). **F** Lipid peroxidation detection of Fadu and AMC-HN-8 cells using BODIPY C11 (*n* = 3). **G** Intracellular GSH analysis of Fadu and AMC-HN-8 cells (*n* = 3). FN: *Fusobacterium nucleatum*. AdiCM: adipocyte supernatant. GSH: glutathione. Data are presented as means ± SEM. * *P* < 0.05,‌** *P* < 0.01, ‌*** *P* < 0.001, **** *P* < 0.0001, ns: no statistical significance

We further explored the role of *F. nucleatum*-reprogrammed adipocytes in the cisplatin resistance of HNSCC. In the vivo study, we established xenograft models by subcutaneously injecting Fadu cells into both groin (adipocyte-rich) and back (adipocyte-poor) regions of nude mice and randomly divided mice into two groups, one with *F. nucleatum* infection and one without (Fig. 3A and B). Notably, *F. nucleatum* infection significantly enhanced tumor growth (volume: *P* = 0.1565 and 0.0039, mass: *P* = 0.0294 and 0.0015), with groin tumors exhibiting greater volume and mass compared to back tumors (volume: *P* = 0.0341 and 0.0018, mass: *P* = 0.0201 and 0.0012) (Fig. 3C and D). Moreover, *F. nucleatum* exerted a stronger pro-tumor effect in the groin compared to the back (volume: *P* = 0.0257, mass: *P* = 0.0208). This anatomical disparity in tumor progression strongly implicated *F. nucleatum*-modified adipocytes in promoting cisplatin resistance.

Complementary in vitro experiments using conditioned medium from adipocyte cultures revealed that *F. nucleatum*-exposed adipocyte secretions substantially increased the IC50 of cisplatin in both Fadu and HN8 cell lines (*P* = 0.0013 and 0.0020) (Fig. 3E). Mechanistic investigations demonstrated that while cisplatin normally elevated lipid peroxidation - a key antitumor mechanism - this effect was significantly attenuated by conditioned medium from *F. nucleatum*-exposed adipocytes (Fig. 3F). Further analysis identified concomitant increases in intracellular glutathione (GSH) levels (*P* = 0.0023 and 0.0331) (Fig. 3G), which was an important reductive metabolite in cells. Therefore, *F. nucleatum*-reprogrammed adipocytes conferred cisplatin resistance to HNSCC by elevation of intracellular GSH and lipid peroxidation.

### *F. nucleatum*-reprogrammed adipocytes facilitate tumor cisplatin-resistance through activation of CREB/HSL and secretion of CCL2

**Fig. 4:**
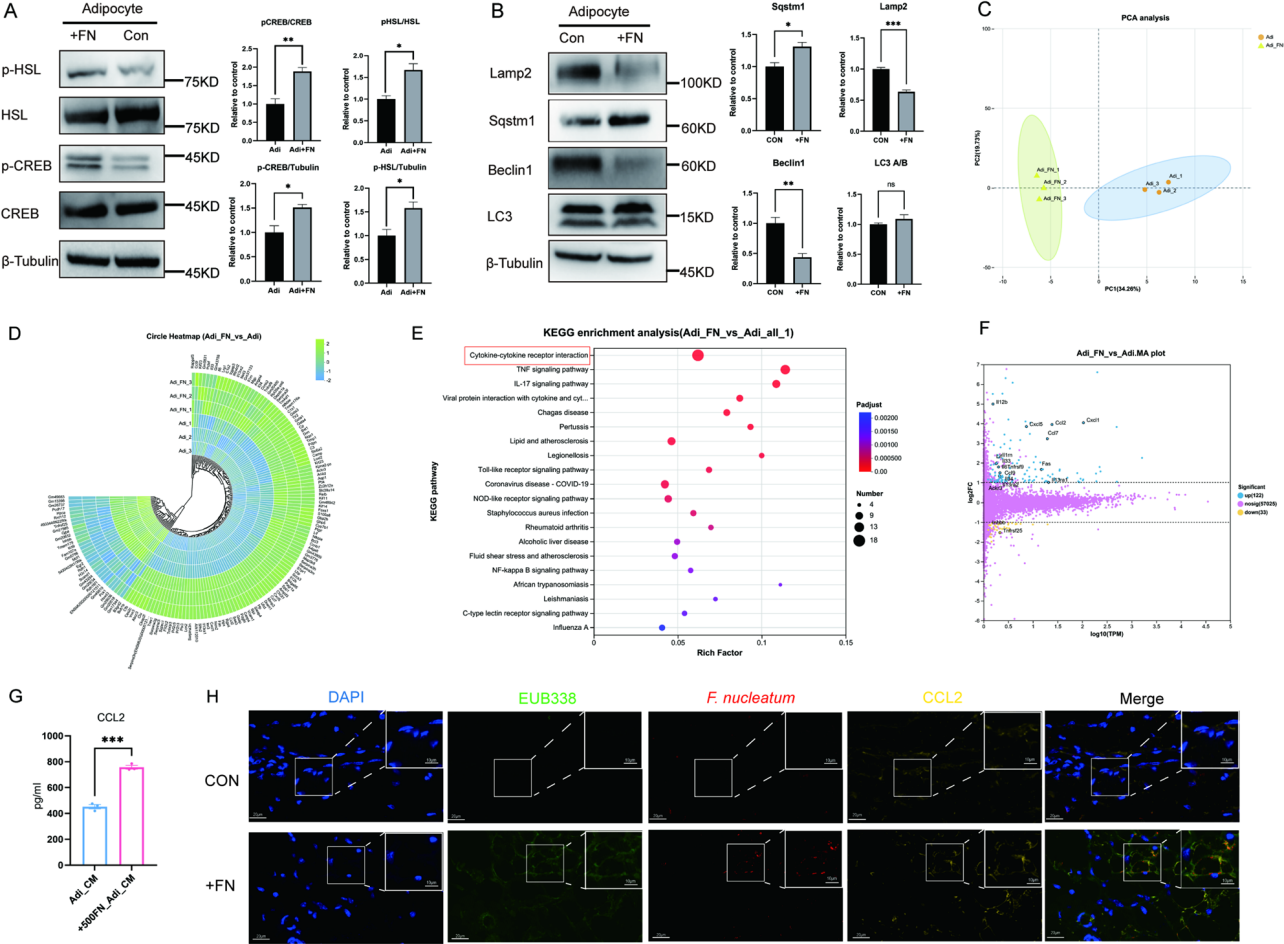
*F. nucleatum*-reprogrammed adipocytes facilitate tumor cisplatin-resistance through activation of CREB/HSL and secretion of CCL2. **A** Western blot analysis of phosphorylation level of CREB/HSL in adipocytes and *F. nucleatum*-cocultured adipocytes (*n* = 3). **B** Western blot analysis of lipophagy-related genes in adipocytes and *F. nucleatum*-cocultured adipocytes (*n* = 3). **C** PCA plot showed the overall differences in transcriptional profile of adipocytes and *F. nucleatum*-cocultured adipocytes. **D** The circle heatmap of differential genes in adipocytes and *F. nucleatum*-cocultured adipocytes. **E** KEGG pathway enrichment. **F** MA plot of differential genes with cytokine-related genes highlighted. **G** ELISA analysis of CCL2 content in the supernatant of adipocytes and *F. nucleatum*-cocultured adipocytes (*n* = 3). **H** Changes in CCL2 expression in inguinal adipose tissue following *F. nucleatum* infection. AdiCM: adipocyte supernatant. FN: *Fusobacterium nucleatum*. Adi: adipocytes. Data are presented as means ± SEM. * *P* < 0.05,‌** *P* < 0.01, ‌*** *P* < 0.001, **** *P* < 0.0001, ns: no statistical significance

To elucidate the mechanistic basis of *F. nucleatum*-induced adipocyte reprogramming and subsequent cisplatin resistance, we investigated the underlying molecular pathways. Our study focused on two principal lipolytic mechanisms in adipocytes: hormone-sensitive lipase (HSL)-mediated lipolysis and lipophagy. *F. nucleatum* coculture significantly enhanced phosphorylation of both CREB and HSL (Fig. 4A), while simultaneously downregulating key lipophagy markers (LAMP2 and beclin1) and upregulating SQSTM1/p62 expression, with no significant alteration in LC3A/B ratios (Fig. 4B). These findings demonstrated that *F. nucleatum* preferentially activates the canonical CREB/HSL lipolytic pathway rather than lipophagic degradation.

Transcriptomic profiling through RNA sequencing provided further mechanistic insights. Principal component analysis (PCA) showed clear segregation between *F. nucleatum*-exposed and control adipocytes (Fig. 4C). Differential gene expression patterns were visualized in a circular heatmap (Fig. 4D), and KEGG pathway analysis revealed significant enrichment in cytokine-mediated signaling pathways (Fig. 4E). Among the most prominently upregulated factors identified in the MA plot (Fig. 4F), CCL2 emerged as a key adipokine, with its overexpression confirmed by ELISA of conditioned media (*P* = 0.0002) (Fig. 4G). Spatial validation through fluorescence in situ hybridization (FISH) coupled with immunofluorescence in murine adipose tissue demonstrated *F. nucleatum*-induced CCL2 upregulation in vivo (Fig. 4H).

Collectively, our results established that *F. nucleatum* reprogrammed adipocytes through two synergistic mechanisms: (1) metabolic activation via CREB/HSL-mediated lipolysis to provide energy substrates, and (2) paracrine signaling through CCL2 secretion to create a tumor-protective microenvironment. This dual-axis reprogramming substantially contributed to the development of cisplatin resistance in HNSCC tumors.

### CCR2 antagonist reverses the protumor cisplatin resistance effects of *F. nucleatum*-reprogrammed adipocytes through downregulation of SLC1A5 and SLC7A11

**Fig. 5:**
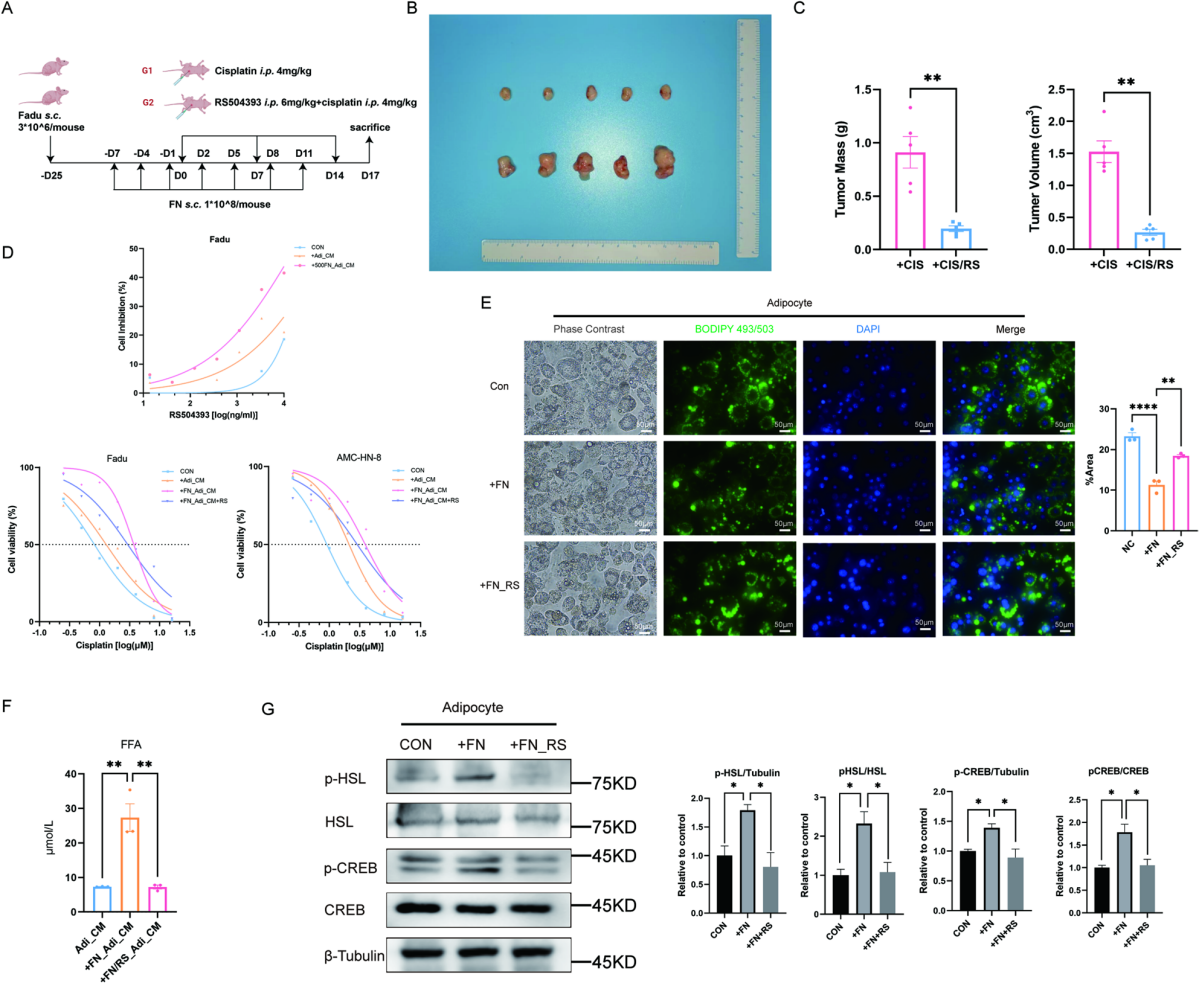
CCR2 antagonist reverses the protumor cisplatin resistance effects of *F. nucleatum*-reprogrammed adipocytes. **A** Schematic of BALB/c-Nude mouse modelling protocol. **B** Photos of right groin tumors obtained from nude mice (*n* = 5). From top to bottom: Control, RS504393-treated. **C** The analysis of volume and mass of the obtained tumors (*n* = 5). **D** The change of cell inhibitory rate in Fadu cells after adding supernatant of normal culture medium, adipocytes, and *F. nucleatum*-cocultured adipocytes (top). The change of cisplatin IC50 in Fadu and AMC-HN-8 cells after adding normal culture medium (0.8190, 95%CI 0.7346 to 0.9108 and 0.9079, 95%CI 0.8446 to 0.9759), supernatant of adipocyte (1.285, 95%CI 1.065 to 1.540 and 2.103, 95%CI 1.970 to 2.244), *F. nucleatum*-cocultured adipocytes (3.701, 95%CI 3.378 to 4.047 and 3.880, 95%CI 3.146 to 4.738), and RS504393 (2.934, 95%CI 2.429 to 3.544 and 2.865, 95%CI 2.365 to 3.458) (bottom) (*n* = 3). **E** The changes of lipid droplet content in adipocytes after coculturing with *F. nucleatum* and after adding RS504393 (*n* = 3). **F** The changes in free fatty acid content of the adipocytes’ supernatant after coculturing with *F. nucleatum* and after adding RS504393 (*n* = 3). **G** The changes in phosphorylation level of CREB/HSL in adipocytes after coculturing with *F. nucleatum* and after adding RS504393 (*n* = 3). FN: *Fusobacterium nucleatum*. AdiCM: adipocyte supernatant. FFA: free fatty acid. RS: RS504393. Data are presented as means ± SEM. * *P* < 0.05,‌** *P* < 0.01, ‌*** *P* < 0.001, **** *P* < 0.0001, ns: no statistical significance

Given that CCR2 is the main receptor for CCL2, we investigated whether blocking CCR2 could reverse the cisplatin resistance induced by *F. nucleatum*-reprogrammed adipocytes. Using the CCR2 antagonist RS504393 in our in vivo studies (Fig. 5A), we found that combining RS504393 with cisplatin significantly reduced tumor volume and mass in *F. nucleatum*-infected mice compared to cisplatin alone (*P* = 0.0011 and 0.0075) (Fig. 5B and C), demonstrating the anti-resistance effect of CCR2 blockade. In vitro experiments showed that RS504393 inhibited Fadu cell proliferation in a dose-dependent manner, with the strongest effect observed when cells were cultured with conditioned medium from *F. nucleatum*-exposed adipocytes (*P* = 0.0078 and 0.0013). Importantly, RS504393 treatment significantly lowered the IC50 of cisplatin in HNSCC cells exposed to this conditioned medium (*P* = 0.0324 and 0.0417) (Fig. 5D). Further mechanistic studies revealed that RS504393 treatment increased lipid droplet accumulation (Fig. 5E), decreased free fatty acid release (*P* = 0.0018) (Fig. 5F), and reduced phosphorylation of CREB and HSL in *F. nucleatum*-exposed adipocytes (Fig. 5G).

These findings demonstrated that *F. nucleatum*-reprogrammed adipocytes promoted cisplatin resistance through CCL2-mediated paracrine signaling, which could be reversed by CCR2 blockade. Also, *F. nucleatum* induced adipocyte lipolysis through CCL2 autocrine signaling, and that CCR2 inhibition also reversed this metabolic reprogramming, thereby reducing the energy supply to tumor cells. Together, these results showed that adipocyte-secreted CCL2 contributed to cisplatin resistance through both paracrine signaling to tumor cells and autocrine stimulation of lipolysis in adipocytes, with both pathways being effectively targeted by CCR2 blockade.

**Fig. 6:**
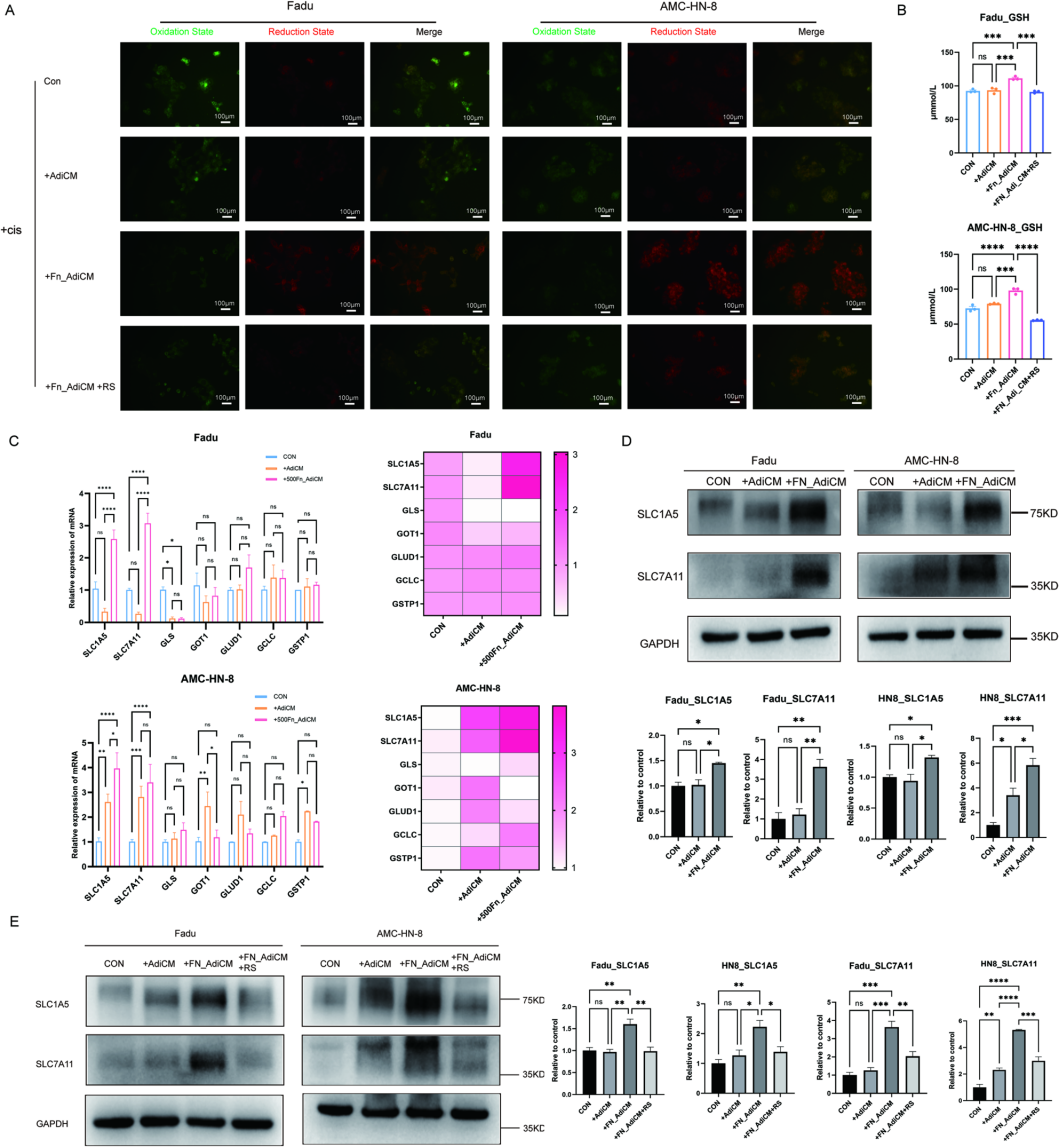
CCR2 antagonist reverses cisplatin resistance through downregulation of SLC1A5 and SLC7A11. **A** The change of lipid peroxidation in Fadu and AMC-HN-8 cells after adding supernatant of adipocyte, *F. nucleatum*-cocultured adipocytes, and RS504393 (*n* = 3). **B** The change of GSH level in Fadu and AMC-HN-8 cells after adding supernatant of adipocyte, *F. nucleatum*-cocultured adipocytes, and RS504393 (*n* = 3). **C** Relative expression level of genes regarding glutamine metabolism in Fadu and AMC-HN-8 cells after coculturing with adipocyte or *F. nucleatum*-cocultured adipocytes (*n* = 3). **D** Western blot analysis of SLC1A5 and SLC7A11 in Fadu and AMC-HN-8 cells after coculturing with adipocyte or *F. nucleatum*-cocultured adipocytes (*n* = 3). **E** Western blot analysis of SLC1A5 and SLC7A11 in Fadu and AMC-HN-8 cells after treatment with RS504393 (*n* = 3). FN: *Fusobacterium nucleatum*. AdiCM: adipocyte supernatant. RS: RS504393. GSH: glutathione. Data are presented as means ± SEM. * *P* < 0.05,‌** *P* < 0.01, ‌*** *P* < 0.001, **** *P* < 0.0001, ns: no statistical significance

To further clarify the underlying mechanism of CCR2 blocking and cisplatin resistance, our study explored the possible downstream targets in HNSCC. Our mechanistic investigations revealed that CCR2 blockade with RS504393 effectively reversed the redox imbalance induced by *F. nucleatum*-reprogrammed adipocytes, restoring normal levels of lipid peroxidation (Fig. 6A) and reducing excessive intracellular GSH accumulation (*P* = 0.0001 and < 0.0001) (Fig. 6B). Since GSH homeostasis was tightly coupled with glutamine metabolism, we further examined the expression of key glutamine metabolic regulators. We found that conditioned medium from *F. nucleatum*-exposed adipocytes significantly upregulated both mRNA and protein expression of the glutamine transporters SLC1A5 and SLC7A11 in HNSCC cells (Fig. 6C and D). Notably, this metabolic reprogramming was effectively counteracted by RS504393 treatment (Fig. 6E), demonstrating CCR2’s pivotal role in mediating these effects. These findings delineated a complete signaling cascade in which *F. nucleatum*-reprogrammed adipocytes activated the CCL2/CCR2 axis to upregulate glutamine transporters, thereby enhancing GSH synthesis and conferring cisplatin resistance - a process that could be therapeutically targeted through CCR2 inhibition.

## Discussion

The focus of our study is the potential role of *F. nucleatum*-adipocyte interaction in HNSCC progression. It has been established in multiple cancers, especially in lipid-rich cancers such as breast, prostate and pancreatic cancer, that adipocytes residing alongside tumor cells acquire unique traits, marked by upregulated lipolysis and paracrine release of tumor-promoting factors [22, 23]. Also, studies have explored the interaction between microbes and adipocytes in the progression of obesity and other metabolic disorders [24, 25]. Accumulating evidence indicates that gut microbiota contribute to adipose tissue inflammation and the development of obesity through mechanisms involving gut barrier dysfunction [26]. Notably, lipopolysaccharide (LPS) from pathogens, including *F. nucleatum*, has been reported to stimulate adipocytes to produce IL-6, a key pro-tumor cytokine [27]. Nevertheless, the direct impact of microorganism-induced functional remodeling of adipocytes on cancer progression remains underexplored. Only in it has shown that Epstein–Barr virus drives adipocyte dedifferentiation in the bone marrow of nasopharyngeal carcinoma patients, generating a protumorigenic niche that supports cancer progression [28]. This study parallels our observation in the metastatic neck nodes of HNSCC that microbiota could directly reprogram adipocytes to foster tumor progression. Furthermore, a prospective observational study investigating the microbiome–adipose tissue axis in rectal cancer is currently recruiting [29]. Moving forward, more research is warranted to elucidate the role of microbe–adipocyte crosstalk in other lipid-rich tumor microenvironments.

The molecular mechanisms underlying *F. nucleatum*-mediated adipocyte reprogramming may involve bacterial surface components such as LPS [27]. However, the complete repertoire of bacterial factors and host signaling pathways involved in this process remains to be elucidated through more systematic mechanistic studies. Our work not only expands the current understanding of microbiota-host cell interactions in the tumor microenvironment but also highlights the need for further investigation into microbial contributions to metabolic reprogramming in the cancer microenvironment.

Nodal necrosis presents as a prevalent clinical feature in HNSCC. It has been reported that the incidence of nodal necrosis was nearly 22.9% for nasopharyngeal carcinoma [30], which is significantly higher than the primary tumor (2% vs. 21.5%) [31]. Previous large-scale clinical studies have established lymph node necrosis as an independent predictor of treatment resistance in HNSCC [32, 33]. It has been reported that tumors exceeding 4 mm in radial expansion frequently develop hypoxia and nutrient deprivation, potentially inducing necrotic changes and conferring treatment resistance [34, 35]. This hypoxic environment may partially explain the reason why *F. nucleatum* tends to be enriched in the necrotic metastatic neck nodes. Previous studies have also revealed shared microbial signatures in metastatic lymph nodes and primary tumor sites, suggesting microbial dissemination during tumor progression [36]. Also, researchers have found that during metastatic dissemination, circulating tumor cells (CTCs) and their intracellular microbiota establish a mutualistic relationship - bacterial symbionts enhance CTC survival under hemodynamic shear stress, while tumor cells serve as vectors for microbial dissemination to distant sites [37]. Thus, CTCs may serve as vectors for *F. nucleatum* dissemination, preferentially delivering them to their favored metastatic niche - necrotic lymph nodes. However, this intriguing hypothesis requires further experimental validation to establish the precise mechanisms of microbial trafficking during metastasis.

CCL2, the prototypical member of the CC chemokine family, has emerged as a critical mediator of tumor progression and therapeutic resistance. Clinical studies across diverse malignancies have consistently demonstrated that elevated CCL2 expression correlates with adverse clinical outcomes [38, 39], with mechanistic investigations revealing its multifaceted role in promoting drug resistance through various molecular pathways [40, 41]. Within the complex tumor microenvironment, multiple cellular constituents - including malignant cells, adipocytes, cancer-associated fibroblasts, and tumor-associated macrophages - contribute to CCL2 production through both paracrine and autocrine signaling mechanisms. It has been reported that in esophageal carcinogenesis, the CCL2-CCR2 axis mediates immune evasion through the recruitment of tumor-associated macrophages and activation of the PD-1 signaling pathway [42]. Thus, the resulting CCL2 gradient activates CCR2-dependent signaling cascades that collectively foster a tumor-permissive niche. In the future, a deeper investigation into the role of CCL2 in the tumor microenvironment may hold promise for developing strategies to overcome drug resistance.

Our current study provides novel evidence that *F. nucleatum* infection triggers significant CCL2 secretion from adipocytes, which in turn promotes cisplatin resistance in HNSCC through dual autocrine and paracrine mechanisms. Importantly, we demonstrate that pharmacological inhibition of CCR2 using the specific antagonist RS504393 effectively reverses this resistance phenotype. In previous studies, the combination of RS504393 and IL-6 blockade synergistically produced a robust antitumor response in orthotopic HNSCC mouse models [19]. These preclinical findings gain translational relevance from ongoing clinical trials showing that CCR2 blockade enhances the efficacy of conventional chemotherapy regimens like FOLFIRINOX [43]. While these results are promising, more dedicated preclinical and clinical studies are needed to evaluate the therapeutic potential of CCR2 inhibitors specifically in tumor patients, particularly those with *F. nucleatum*-associated or adipocyte-rich tumors.

*F. nucleatum*-reprogrammed adipocytes induce cisplatin resistance in HNSCC cells by orchestrating a profound metabolic shift characterized by enhanced glutathione (GSH) biosynthesis and suppressed lipid peroxidation. These findings align with established mechanisms of chemoresistance, as glutamate - a key metabolic derivative of glutamine - serves as the fundamental precursor for GSH synthesis. The critical involvement of glutamine metabolic reprogramming in cisplatin resistance has been well-documented in prior studies [44], with glutamine deprivation shown to sensitize ovarian and lung cancer cells to cisplatin by depleting intracellular GSH pools [45, 46]. Notably, *F. nucleatum*-modified adipocytes drive the upregulation of glutamine transporters (SLC1A5/SLC7A11) in HNSCC cells, establishing a metabolic program that sustains GSH production and confers chemoresistance. This observation complements existing reports that overexpression of amino acid transporters (e.g., SLC38A5) promotes tumor cell survival through glutamine metabolic rewiring and subsequent cisplatin desensitization [47]. While our current work establishes the crucial link between CCL2-CCR2 signaling and glutamine metabolic reprogramming, further investigation is warranted to fully elucidate: (1) the precise molecular mechanisms connecting CCR2 activation to transporter upregulation, and (2) potential tissue-specific variations in this metabolic adaptation pathway.

## Conclusion

Our findings demonstrate that *F. nucleatum* drives the transformation of adipocytes into a cancer-associated phenotype, which subsequently promotes cisplatin resistance in necrotic lymph nodes of HNSCC through activation of the CCL2-CCR2 signaling axis. These results not only reveal a novel mechanism of microbial-mediated chemoresistance but also suggest that targeting this tumor-adipocyte-microbe interplay can represent a promising strategy to improve cisplatin efficacy in HNSCC treatment.

## Supplementary Information

Supplementary Material 1.

Supplementary Material 2: Supplemental Table 1. Basic characteristics of included patients.

Supplementary Material 3: Supplemental Table 2. DNA probs.

Supplementary Material 4: Supplemental Table 3. Details of antibodies.

Supplementary Material 5: Supplemental Table 4. Sequences of primers and probes.

## Data Availability

Datasets generated and/or analyzed during the current study are available from the corresponding author on reasonable request.

## References

[1] Hanai N, Ozawa T, Hirakawa H, et al. The nodal response to chemoselection predicts the risk of recurrence following definitive chemoradiotherapy for pharyngeal cancer [J]. Acta Otolaryngol. 2014;134(8):865–71. 25022795 10.3109/00016489.2014.894252

[2] Wolf G T, Fisher SG. Effectiveness of salvage neck dissection for advanced regional metastases when induction chemotherapy and radiation are used for organ preservation [J]. Laryngoscope. 1992;102(8):934–9. 1495357 10.1288/00005537-199208000-00015

[3] Liauw SL, Amdur RJ, Morris CG, et al. Isolated neck recurrence after definitive radiotherapy for node-positive head and neck cancer: salvage in the dissected or undissected neck [J]. Head Neck. 2007;29(8):715–9. 17274056 10.1002/hed.20580

[4] Li F, Hsueh C, Huang H, et al. A nomogram to predict nodal response after induction chemotherapy for hypopharyngeal carcinoma [J]. Laryngoscope. 2022; 133(4):849–55 10.1002/lary.30241 35699589

[5] Huang CK, Chang PH, Kuo WH, et al. Adipocytes promote malignant growth of breast tumours with monocarboxylate transporter 2 expression via β-hydroxybutyrate [J]. Nat Commun. 2017;8:14706. 28281525 10.1038/ncomms14706 PMC5353665

[6] Takehara M, Sato Y, Kimura T, et al. Cancer-associated adipocytes promote pancreatic cancer progression through SAA1 expression [J]. Cancer Sci. 2020;111(8):2883–94. 32535957 10.1111/cas.14527 PMC7419047

[7] Hu W, Ru Z, Zhou Y, et al. Lung cancer-derived extracellular vesicles induced myotube atrophy and adipocyte lipolysis via the extracellular IL-6-mediated STAT3 pathway [J]. Biochim Biophys Acta Mol Cell Biol Lipids. 2019;1864(8):1091–102. 31002945 10.1016/j.bbalip.2019.04.006

[8] Nieman KM, Kenny HA, Penicka CV, et al. Adipocytes promote ovarian cancer metastasis and provide energy for rapid tumor growth [J]. Nat Med. 2011;17(11):1498–503. 22037646 10.1038/nm.2492 PMC4157349

[9] He JY, Wei XH, Li SJ, et al. Adipocyte-derived IL-6 and leptin promote breast cancer metastasis via upregulation of Lysyl Hydroxylase-2 expression [J]. Cell Commun Signal. 2018;16(1):100. 30563531 10.1186/s12964-018-0309-z PMC6299564

[10] Zhang Q, Deng T, Zhang H et al. Adipocyte-Derived Exosomal MTTP suppresses ferroptosis and promotes chemoresistance in colorectal cancer [J]. Advanced science (Weinheim, Baden-Wurttemberg, Germany). 2022;9(28):e2203357. 10.1002/advs.202203357 PMC9534973 35978266

[11] Li K, Bihan M, Yooseph S, et al. Analyses of the microbial diversity across the human Microbiome [J]. PLoS ONE. 2012;7(6):e32118. 22719823 10.1371/journal.pone.0032118 PMC3374608

[12] Gong HL, Shi Y, Zhou L, et al. The composition of Microbiome in larynx and the throat biodiversity between laryngeal squamous cell carcinoma patients and control population [J]. PLoS ONE. 2013;8(6):e66476. 23824228 10.1371/journal.pone.0066476 PMC3688906

[13] Gong H, Shi Y, Zhou X, et al. Microbiota in the throat and risk factors for laryngeal carcinoma [J]. Appl Environ Microbiol. 2014;80(23):7356–63. 25239901 10.1128/AEM.02329-14 PMC4249186

[14] Hsueh CY, Gong H, Cong N, et al. Throat microbial community structure and functional changes in postsurgery laryngeal carcinoma patients [J]. Appl Environ Microbiol. 2020;86(24):e01849–20. 10.1128/AEM.01849-20 PMC7688240 33008819

[15] Gong H, Shi Y, XiaO X, et al. Alterations of microbiota structure in the larynx relevant to laryngeal carcinoma [J]. Sci Rep. 2017;7(1):5507. 28710395 10.1038/s41598-017-05576-7 PMC5511217

[16] Hsueh C Y, Huang Q, Gong H, et al. A positive feed-forward loop between Fusobacterium nucleatum and ethanol metabolism reprogramming drives laryngeal cancer progression and metastasis [J]. iScience. 2022;25(2):103829. 35198889 10.1016/j.isci.2022.103829 PMC8851092

[17] Hsueh CY, Lau HC, Huang Q, et al. Fusobacterium nucleatum impairs DNA mismatch repair and stability in patients with squamous cell carcinoma of the head and neck [J]. Cancer. 2022;128(17):3170–84. 35789992 10.1002/cncr.34338

[18] Park HR, Ju EJ, Jo SK, et al. Enhanced antitumor efficacy of cisplatin in combination with HemoHIM in tumor-bearing mice [J]. BMC Cancer. 2009;9:85. 19292900 10.1186/1471-2407-9-85 PMC2666758

[19] Yang F, Yuan C, Chen F, et al. Combined IL6 and CCR2 Blockade potentiates antitumor activity of NK cells in HPV-negative head and neck cancer [J]. J Exp Clin Cancer Res. 2024;43(1):76. 38468260 10.1186/s13046-024-03002-1 PMC10929116

[20] Yu J, Chen Y, Fu X, et al. Invasive Fusobacterium nucleatum May play a role in the carcinogenesis of proximal colon cancer through the serrated neoplasia pathway [J]. Int J Cancer. 2016;139(6):1318–26. 10.1002/ijc.30168 27130618

[21] Furukawa N, Ongusaha P, Jahng WJ, et al. Role of Rho-kinase in regulation of insulin action and glucose homeostasis [J]. Cell Metab. 2005;2(2):119–29. 10.1016/j.cmet.2005.06.011 16098829

[22] Fontana F, Anselmi M. Carollo E, Adipocyte-Derived extracellular vesicles promote prostate cancer cell aggressiveness by enabling multiple phenotypic and metabolic changes [J]. Cells. 2022;11(15):2388. 10.3390/cells11152388 PMC9368412 35954232

[23] Maguire OA, Ackerman SE, Szwed SK, et al. Creatine-mediated crosstalk between adipocytes and cancer cells regulates obesity-driven breast cancer [J]. Cell Metab. 2021;33(3):499–e5126. 10.1016/j.cmet.2021.01.018 PMC7954401 33596409

[24] Newman NK, Zhang Y, Padiadpu J, et al. Reducing gut microbiome-driven adipose tissue inflammation alleviates metabolic syndrome [J]. Microbiome. 2023;11(1):208. 10.1186/s40168-023-01637-4 PMC10512512 37735685

[25] Cani PD, Van Hul M. Gut microbiota in overweight and obesity: crosstalk with adipose tissue [J]. Nat Rev Gastroenterol Hepatol. 2024;21(3):164–83. 10.1038/s41575-023-00867-z 38066102

[26] Cani PD. Crosstalk between the gut microbiota and the endocannabinoid system: impact on the gut barrier function and the adipose tissue [J]. Clin Microbiol Infect. 2012;18(Suppl 4):50–3. 22647050 10.1111/j.1469-0691.2012.03866.x

[27] Yamaguchi M, Nishimura F, Naruishi H, et al. Thiazolidinedione (pioglitazone) blocks P. gingivalis- and F. nucleatum, but not E. coli, lipopolysaccharide (LPS)-induced interleukin-6 (IL-6) production in adipocytes [J]. J Dent Res. 2005;84(3):240–4. 10.1177/154405910508400306 15723863

[28] Liu SC, Tsang NM Leepj, et al. Epstein-Barr virus induces adipocyte dedifferentiation to modulate the tumor microenvironment [J]. Cancer Res. 2021;81(12):3283–94. 10.1158/0008-5472.CAN-20-3121 33824135

[29] Planellas P, Cornejo L, Farrés R et al. Prognostic significance of the microbiome-adipose tissue axis in rectal cancer: protocol of a prospective observational study [J]. BJS Open. 2022;6(2):zrac009. 10.1093/bjsopen/zrac009 PMC8902345 35257139

[30] Chua DT, Sham JS, Kwong DL, et al. Evaluation of cervical nodal necrosis in nasopharyngeal carcinoma by computed tomography: incidence and prognostic significance [J]. Head Neck. 1997;19(4):266–75. 9213104 10.1002/(sici)1097-0347(199707)19:4<266::aid-hed4>3.0.co;2-z

[31] Liang SB, Chen LS, Yang XL, et al. Influence of tumor necrosis on treatment sensitivity and long-term survival in nasopharyngeal carcinoma [J]. Radiother Oncol. 2021;155:219–25. 10.1016/j.radonc.2020.11.011 33217495

[32] Fu JY, Yue XH, Dong M J, et al. Assessment of neoadjuvant chemotherapy with docetaxel, cisplatin, and fluorouracil in patients with oral cavity cancer [J]. Cancer Med. 2023;12(3):2417–26. 10.1002/cam4.5075 PMC9939210 35880556

[33] Jiang Y, Liang Z, Chen K, et al. A dynamic nomogram combining tumor stage and magnetic resonance imaging features to predict the response to induction chemotherapy in locally advanced nasopharyngeal carcinoma [J]. Eur Radiol. 2023;33(3):2171–84. 36355201 10.1007/s00330-022-09201-8

[34] Zapletal E, Vasiljevic T, Busson P et al. Dialog beyond the grave: necrosis in the tumor microenvironment and its contribution to tumor growth [J]. Int J Mol Sci. 2023;24(6):5278. 10.3390/ijms24065278 PMC10049335 36982351

[35] Karsch-Bluman A, Feiglin A, Arbib E, et al. Tissue necrosis and its role in cancer progression [J]. Oncogene. 2019;38(11):1920–35. 10.1038/s41388-018-0555-y 30390074

[36] Rajasekaran K, Carey RM, Lin X, et al. The Microbiome of HPV-positive tonsil squamous cell carcinoma and neck metastasis [J]. Oral Oncol. 2021;117:105305. 10.1016/j.oraloncology.2021.105305 33905914

[37] Fu A, Yao B, Dong T, et al. Tumor-resident intracellular microbiota promotes metastatic colonization in breast cancer [J]. Cell. 2022;185(8):1356–e7226. 10.1016/j.cell.2022.02.027 35395179

[38] Iwamoto H, Izumi K, Nakagawa R et al. Serum CCL2 is a prognostic biomarker for Non-Metastatic Castration-Sensitive prostate cancer [J]. Biomedicines. 2022;10(10):2369. 10.3390/biomedicines10102369 PMC9598117 36289628

[39] Dong Y, Zhang S, ZHAO S, et al. CCL2 promotes lymphatic metastasis via activating RhoA and Rac1 pathway and predict prognosis to some extent in tongue cancer [J]. Cancer Biol Ther. 2023;24(1):2205342. 10.1080/15384047.2023.2205342 PMC10158538 37132640

[40] Qian Y, DING P, Xu J, et al. CCL2 activates AKT signaling to promote Glycolysis and chemoresistance in glioma cells [J]. Cell Biol Int. 2022;46(5):819–28. 10.1002/cbin.11778 35178826

[41] Sun W, Wang X, Wang D et al. CD40×HER2 bispecific antibody overcomes the CCL2-induced trastuzumab resistance in HER2-positive gastric cancer [J]. J Immunother Cancer. 2022;10(7):e005063. 10.1136/jitc-2022-005063 PMC9295658 35851310

[42] Yang H, Zhang Q, Xu M, et al. CCL2-CCR2 axis recruits tumor associated macrophages to induce immune evasion through PD-1 signaling in esophageal carcinogenesis [J]. Mol Cancer. 2020;19(1):41. 32103760 10.1186/s12943-020-01165-x PMC7045401

[43] Nywening TM, Wang-Gillam A, Sanford D E, et al. Targeting tumour-associated macrophages with CCR2 Inhibition in combination with FOLFIRINOX in patients with borderline resectable and locally advanced pancreatic cancer: a single-centre, open-label, dose-finding, non-randomised, phase 1b trial [J]. Lancet Oncol. 2016;17(5):651–62. 10.1016/S1470-2045(16)00078-4 PMC5407285 27055731

[44] Pervushin NV, Yapryntseva MA, Panteleev MA, et al. Cisplatin resistance and metabolism: simplification of complexity [J]. Cancers (Basel). 2024;16(17):3082. 10.3390/cancers16173082 PMC11394643 39272940

[45] Guo J, Satoh K, Tabata S, et al. Reprogramming of glutamine metabolism via glutamine synthetase Silencing induces cisplatin resistance in A2780 ovarian cancer cells [J]. BMC Cancer. 2021;21(1):174. 10.1186/s12885-021-07879-5 PMC7891143 33596851

[46] Liu WJ, Pan PY, Sun Y, et al. Deferoxamine counteracts cisplatin resistance in A549 lung adenocarcinoma cells by increasing vulnerability to glutamine Deprivation-Induced cell death [J]. Front Oncol. 2021;11:794735. 10.3389/fonc.2021.794735 PMC8810525 35127502

[47] Shen X, Wang G, He H, et al. SLC38A5 promotes glutamine metabolism and inhibits cisplatin chemosensitivity in breast cancer [J]. Breast Cancer (Tokyo Japan). 2024;31(1):96–104. 10.1007/s12282-023-01516-8 37914960

