# Supplementary figures and images for: Fusobacterium nucleatum-reprogrammed adipocytes promote tumor cisplatin resistance through the CCL2-CCR2 axis in the necrotic metastatic neck nodes of head and neck carcinoma
Source: Cell Commun Signal. 2025 Nov 24;23:546. doi: 10.1186/s12964-025-02550-z (PMC12750780; doi:10.1186/s12964-025-02550-z)

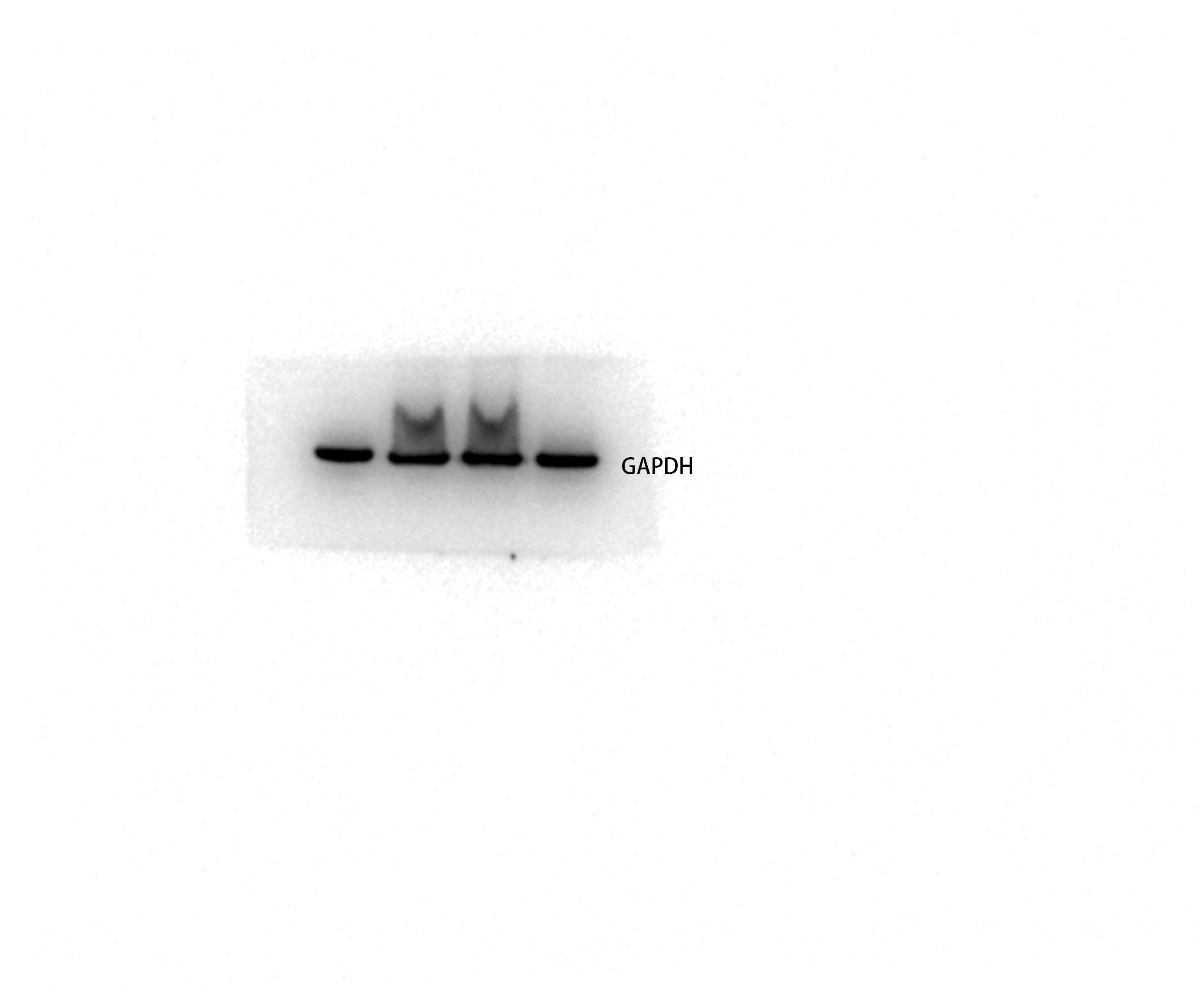

Supplement: Supplementary file 1 — Supplementary Material 1. [file 12964_2025_2550_MOESM1_ESM.zip › Figure6E_HN8_GAPDH.tif]

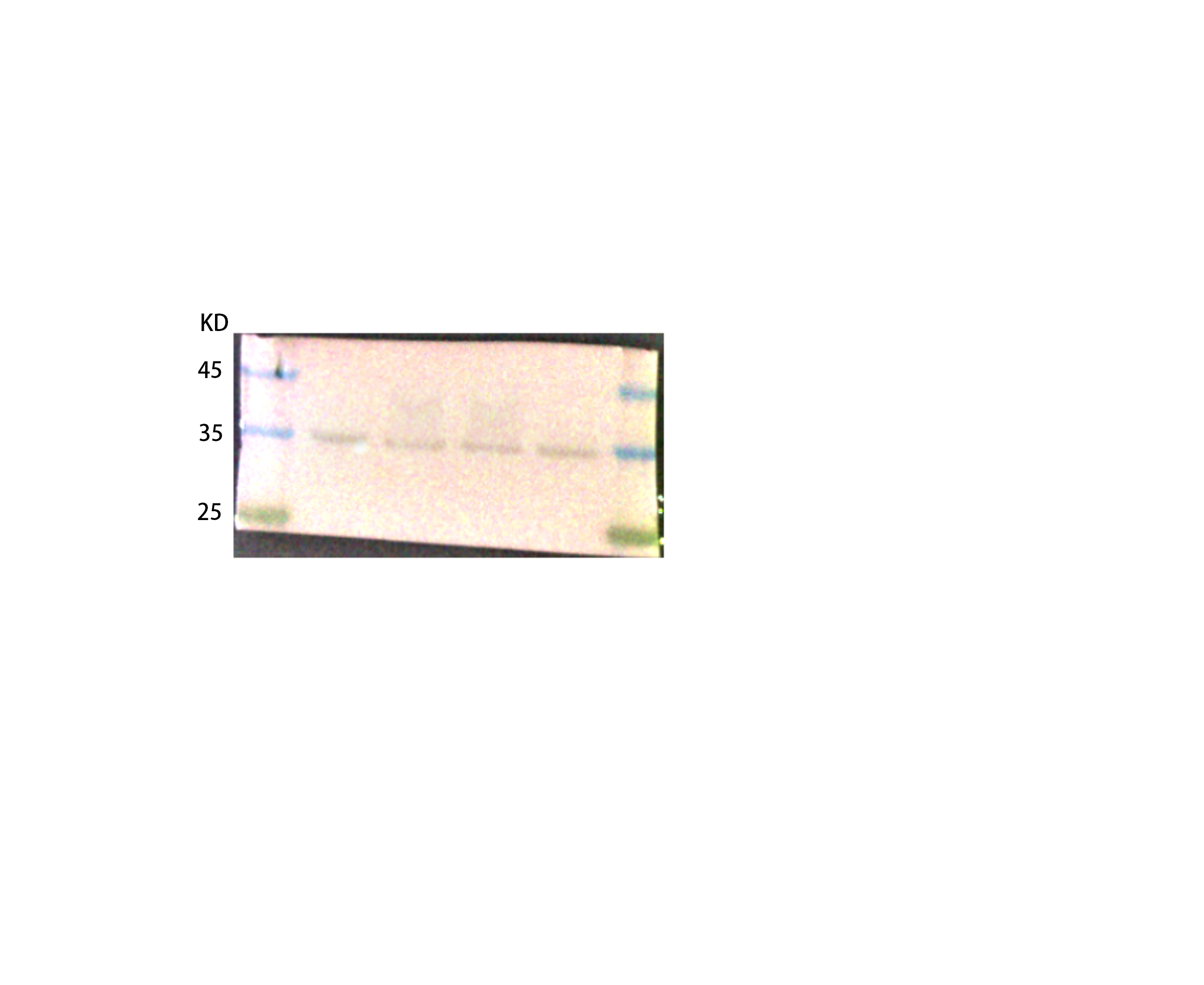

Supplement: Supplementary file 1 — Supplementary Material 1. [file 12964_2025_2550_MOESM1_ESM.zip › Figure6E_HN8_GAPDH+Marker.tif]

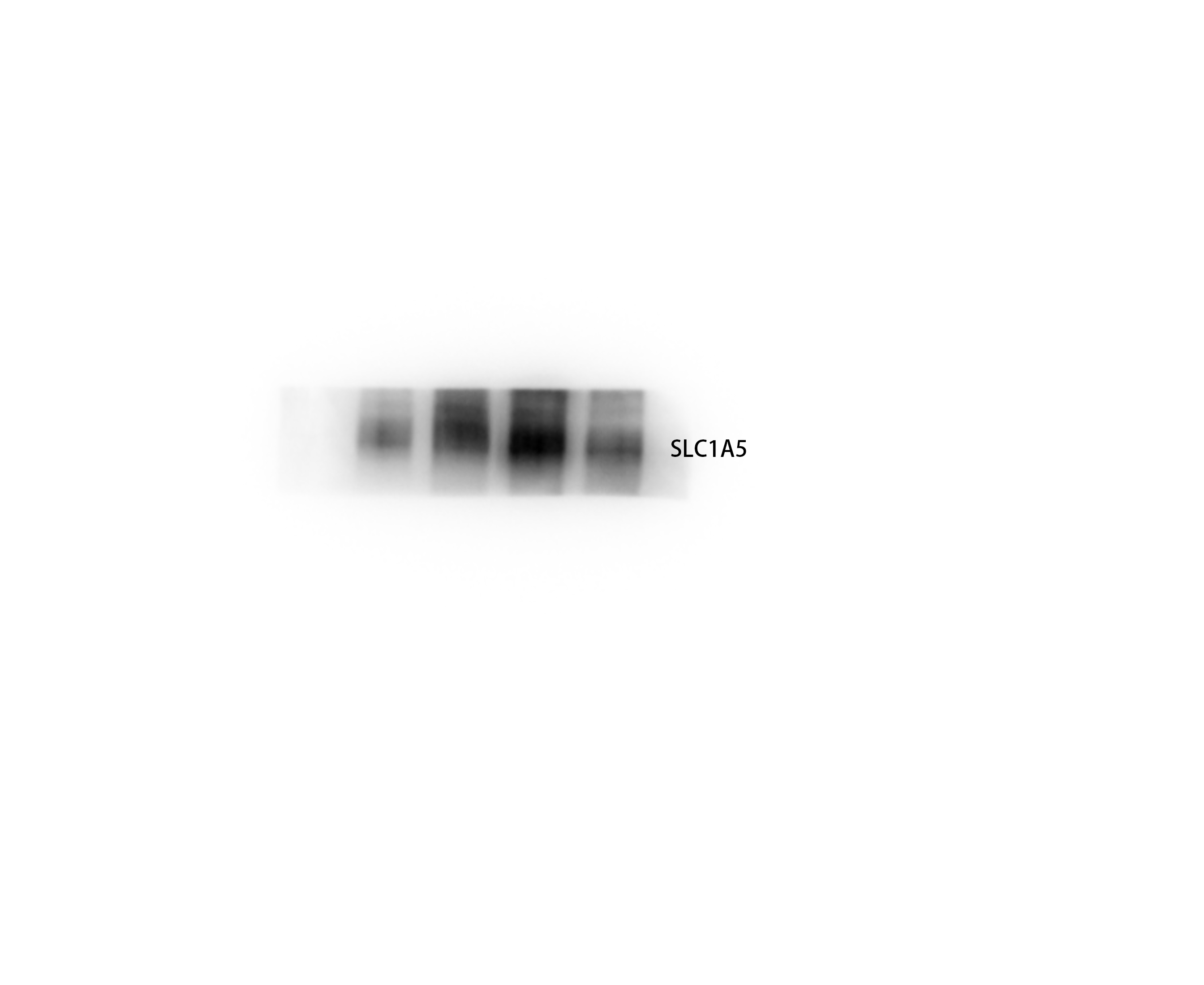

Supplement: Supplementary file 1 — Supplementary Material 1. [file 12964_2025_2550_MOESM1_ESM.zip › Figure6E_HN8_SLC1A5.tif]

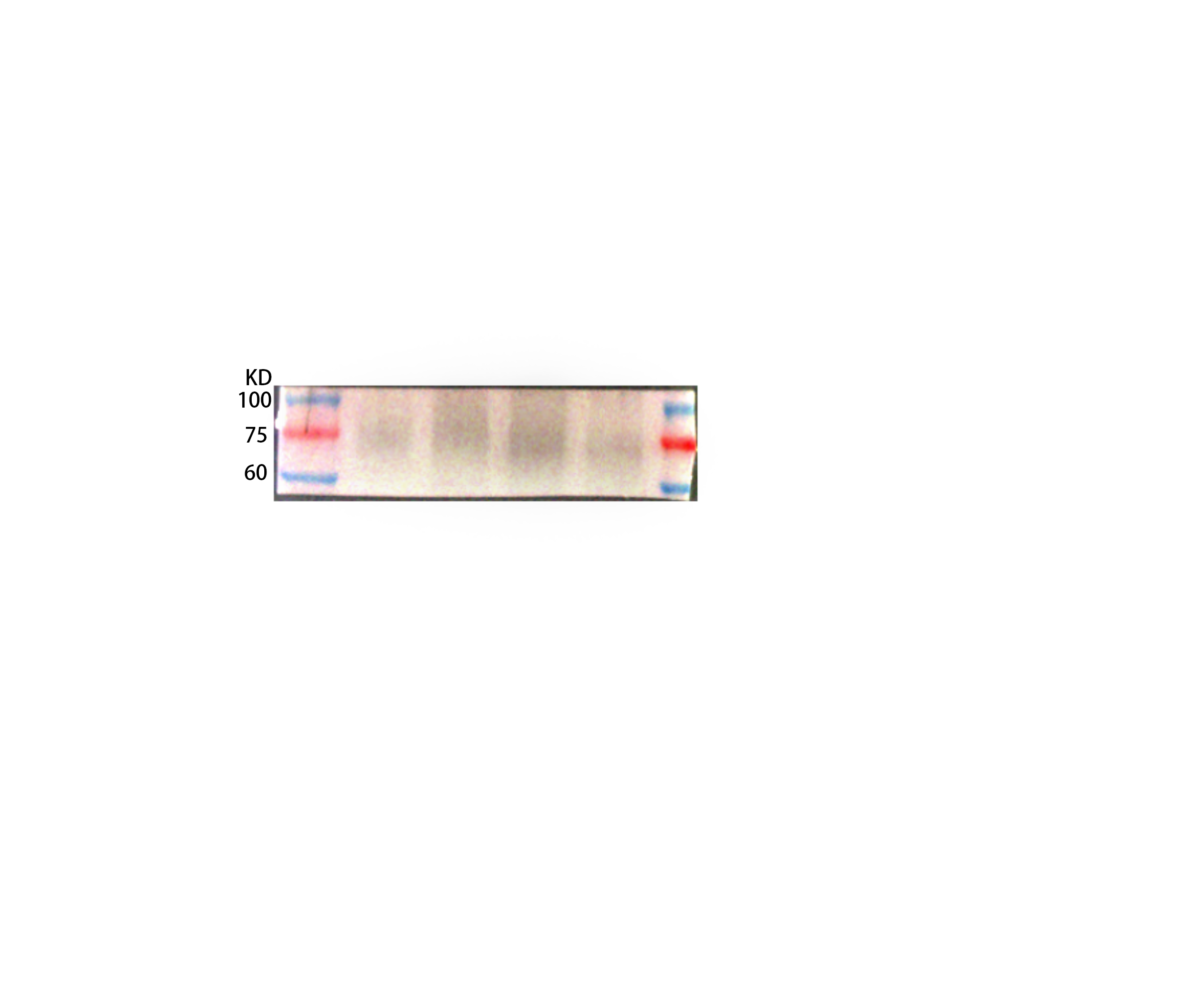

Supplement: Supplementary file 1 — Supplementary Material 1. [file 12964_2025_2550_MOESM1_ESM.zip › Figure6E_HN8_SLC1A5+Marker.tif]

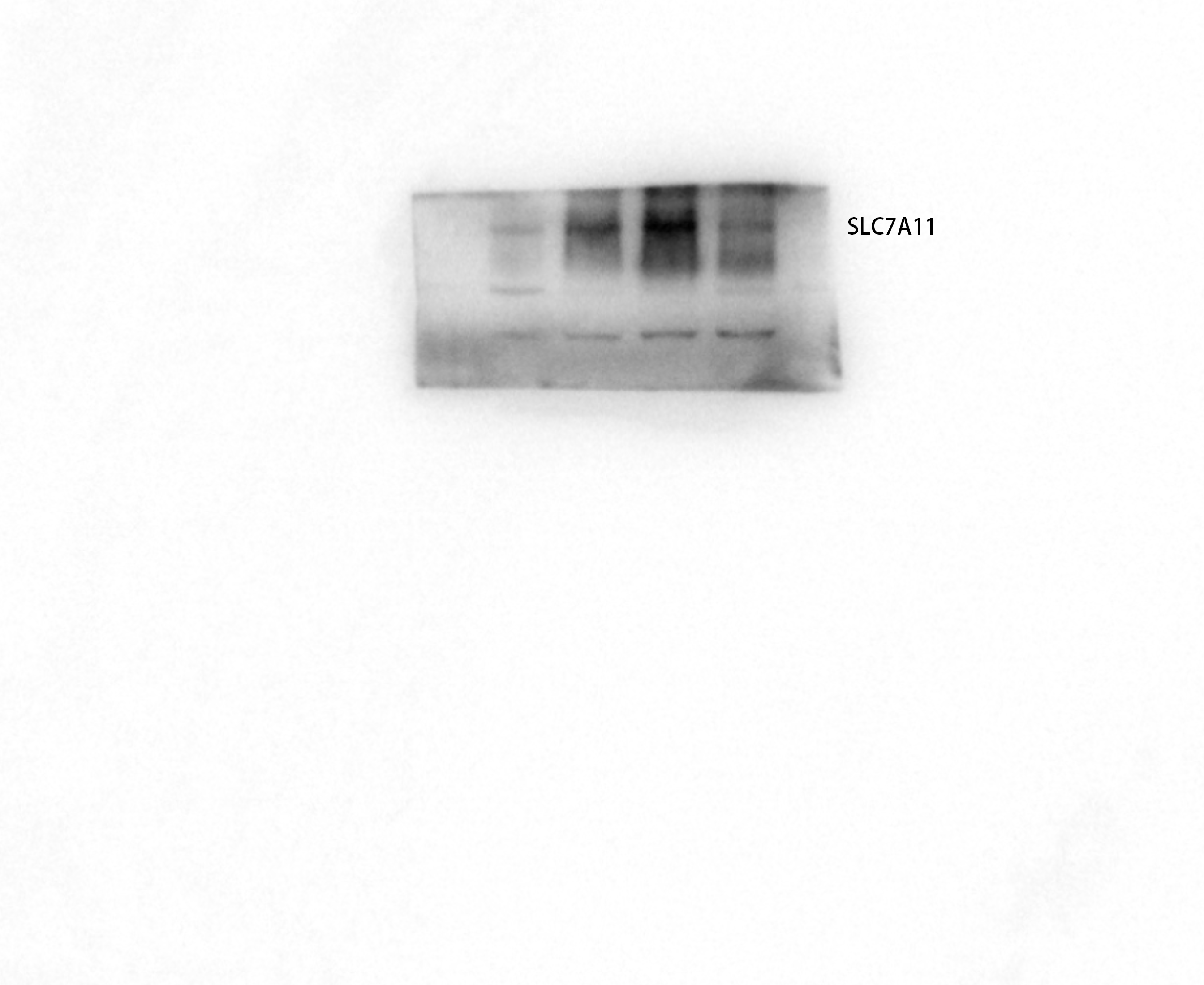

Supplement: Supplementary file 1 — Supplementary Material 1. [file 12964_2025_2550_MOESM1_ESM.zip › Figure6E_HN8_SLC7A11.tif]

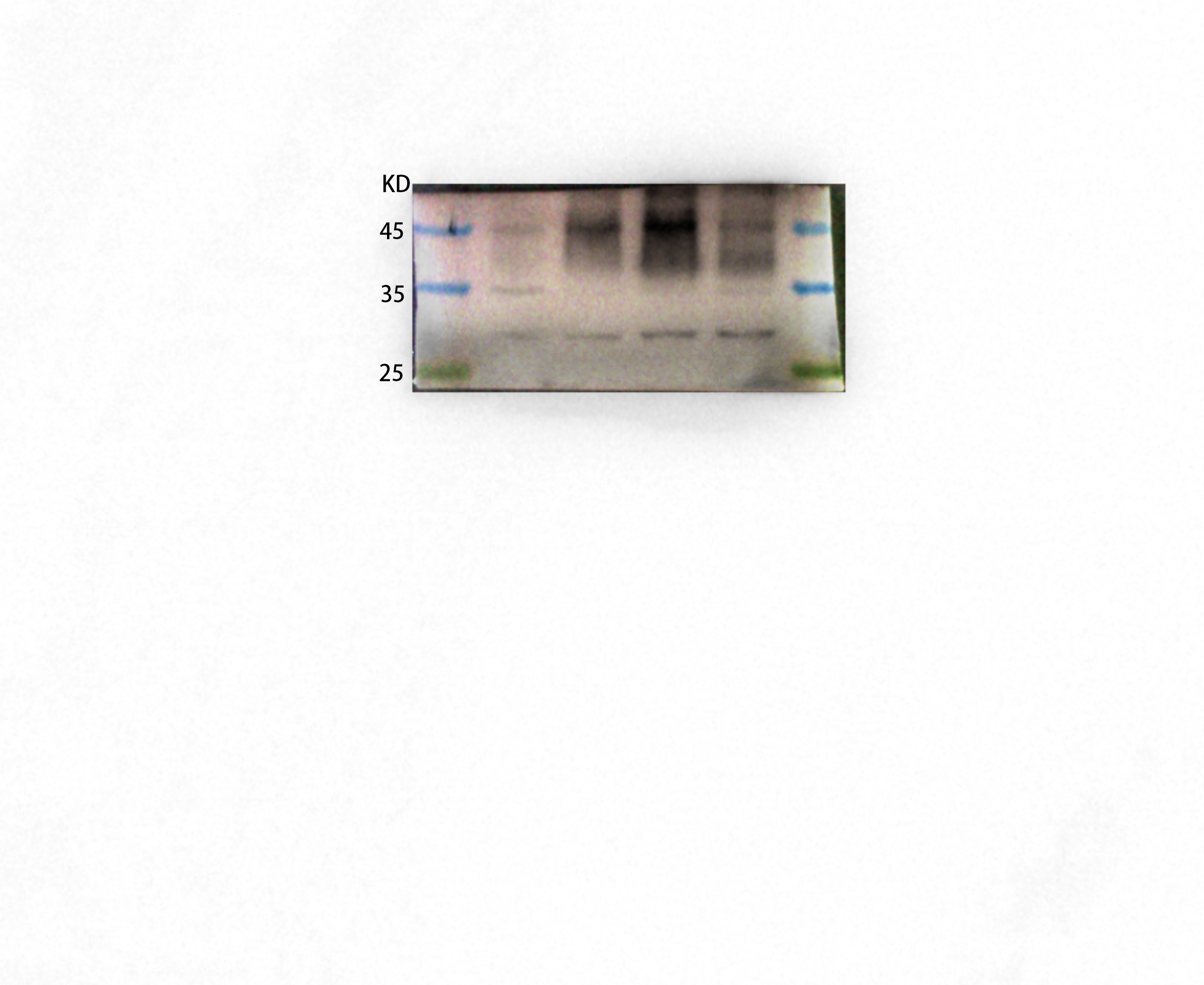

Supplement: Supplementary file 1 — Supplementary Material 1. [file 12964_2025_2550_MOESM1_ESM.zip › Figure6E_HN8_SLC7A11+Marker.tif]

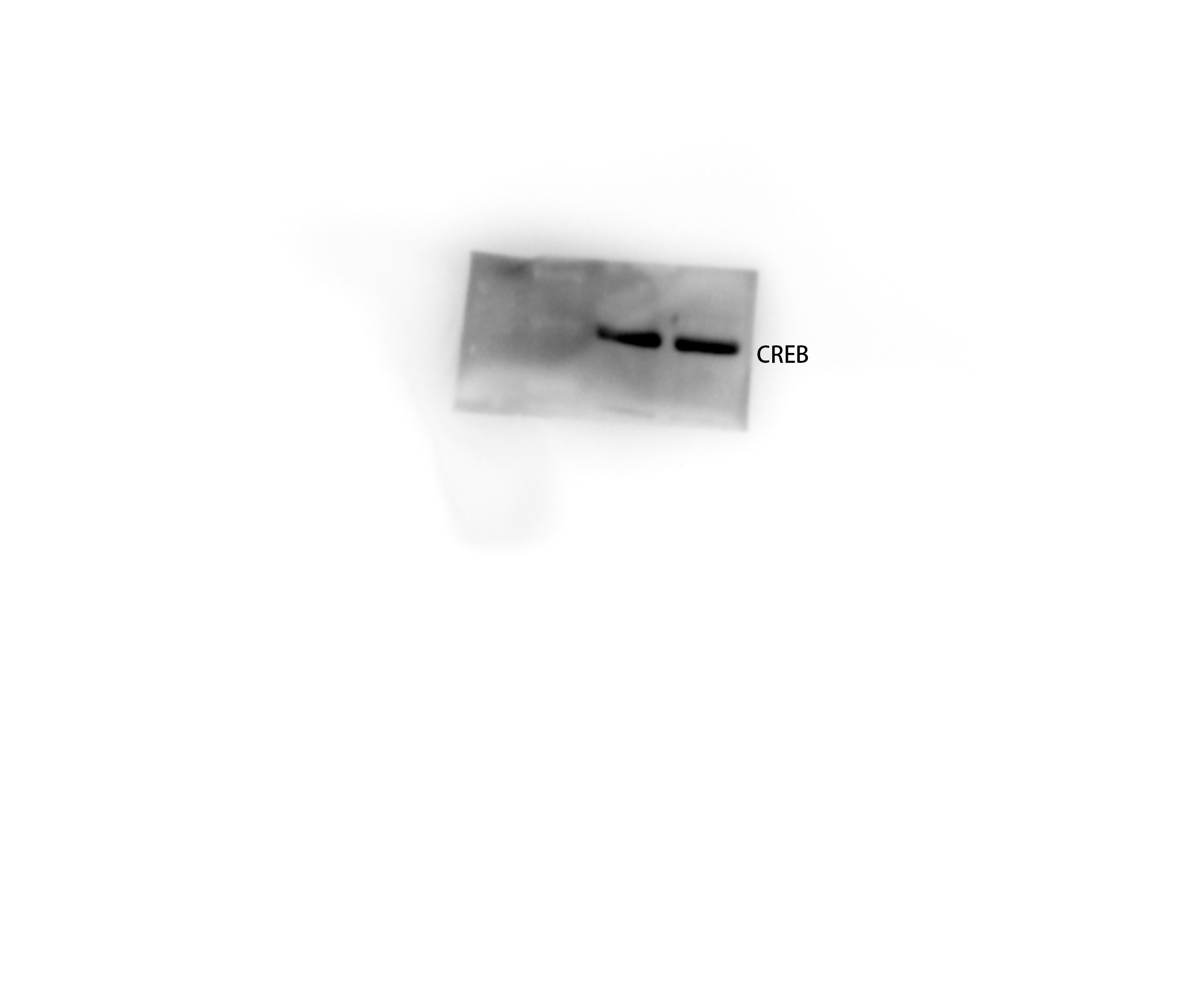

Supplement: Supplementary file 1 — Supplementary Material 1. [file 12964_2025_2550_MOESM1_ESM.zip › Sup_Figure 4A_CREB.tif]

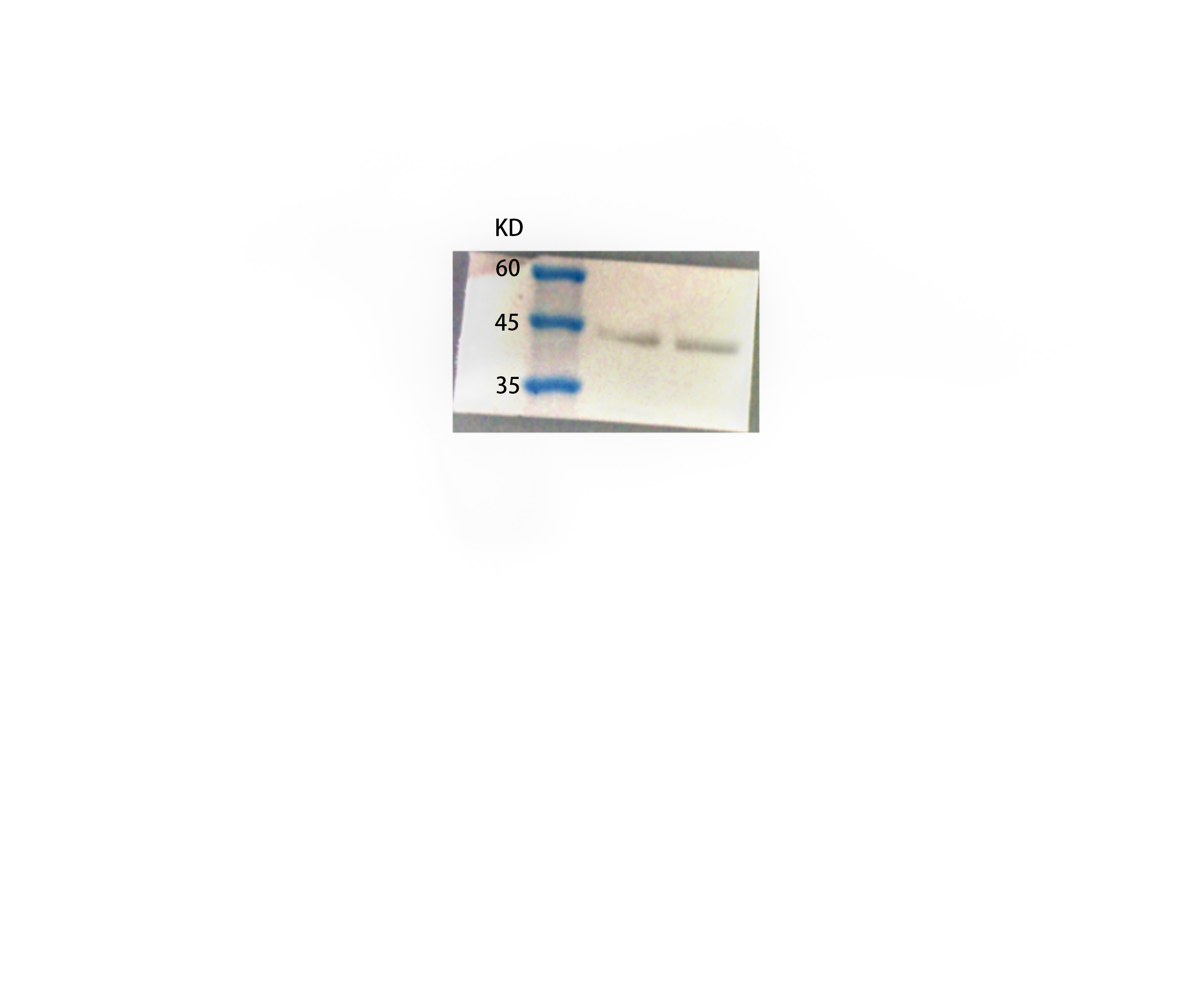

Supplement: Supplementary file 1 — Supplementary Material 1. [file 12964_2025_2550_MOESM1_ESM.zip › Sup_Figure 4A_CREB+Marker.tif]

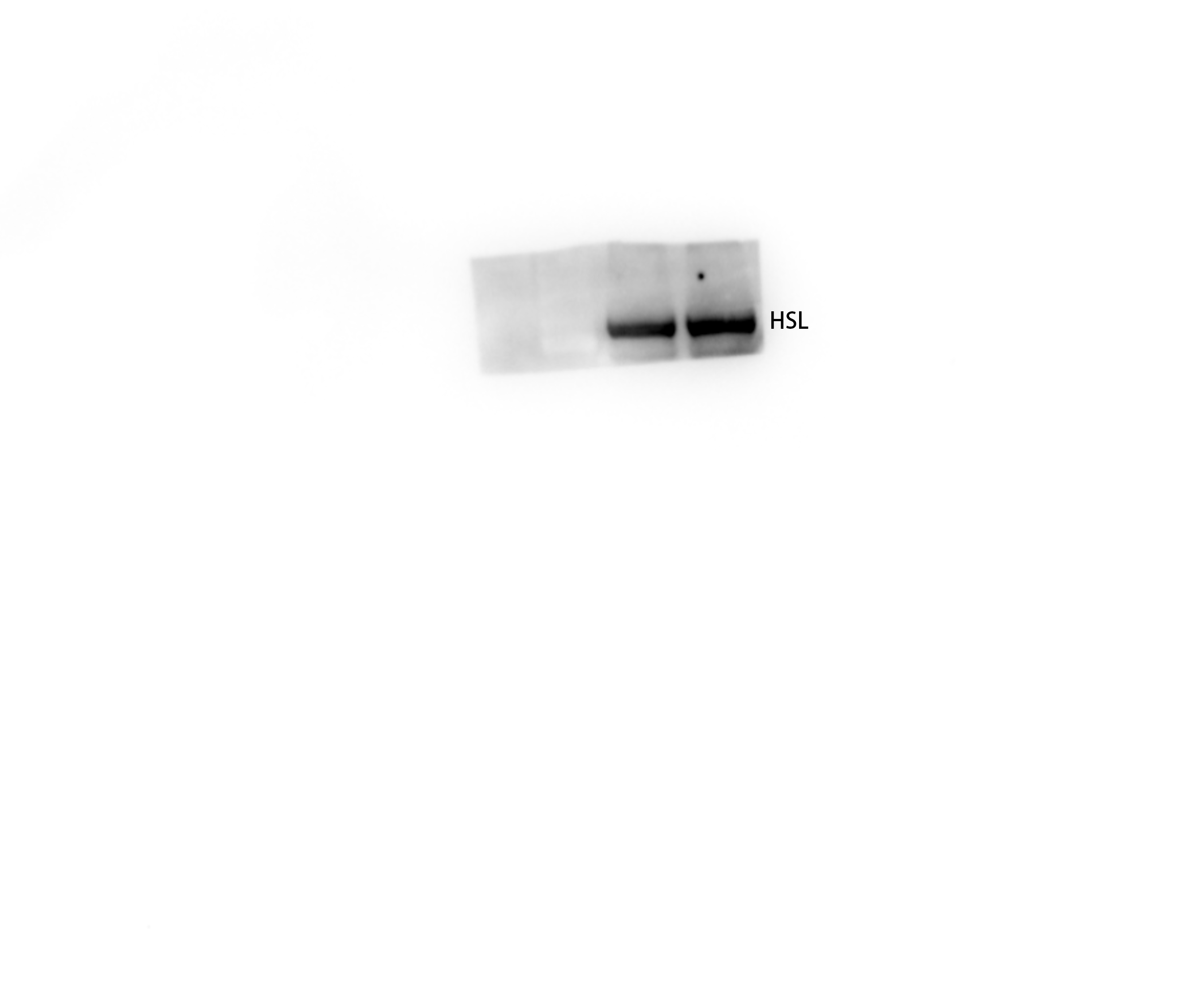

Supplement: Supplementary file 1 — Supplementary Material 1. [file 12964_2025_2550_MOESM1_ESM.zip › Sup_Figure 4A_hsl.tif]

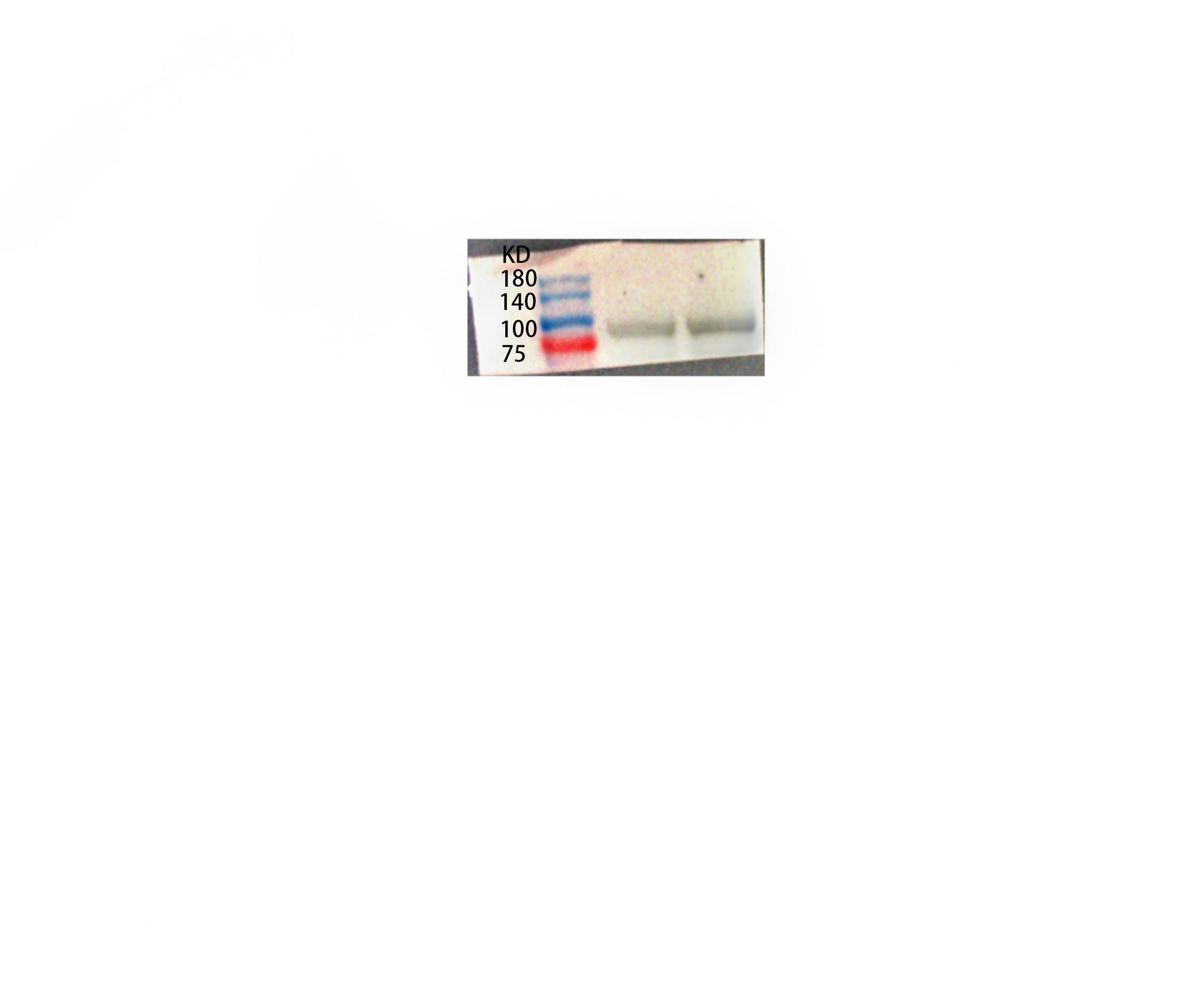

Supplement: Supplementary file 1 — Supplementary Material 1. [file 12964_2025_2550_MOESM1_ESM.zip › Sup_Figure 4A_hsl+Marker.tif]

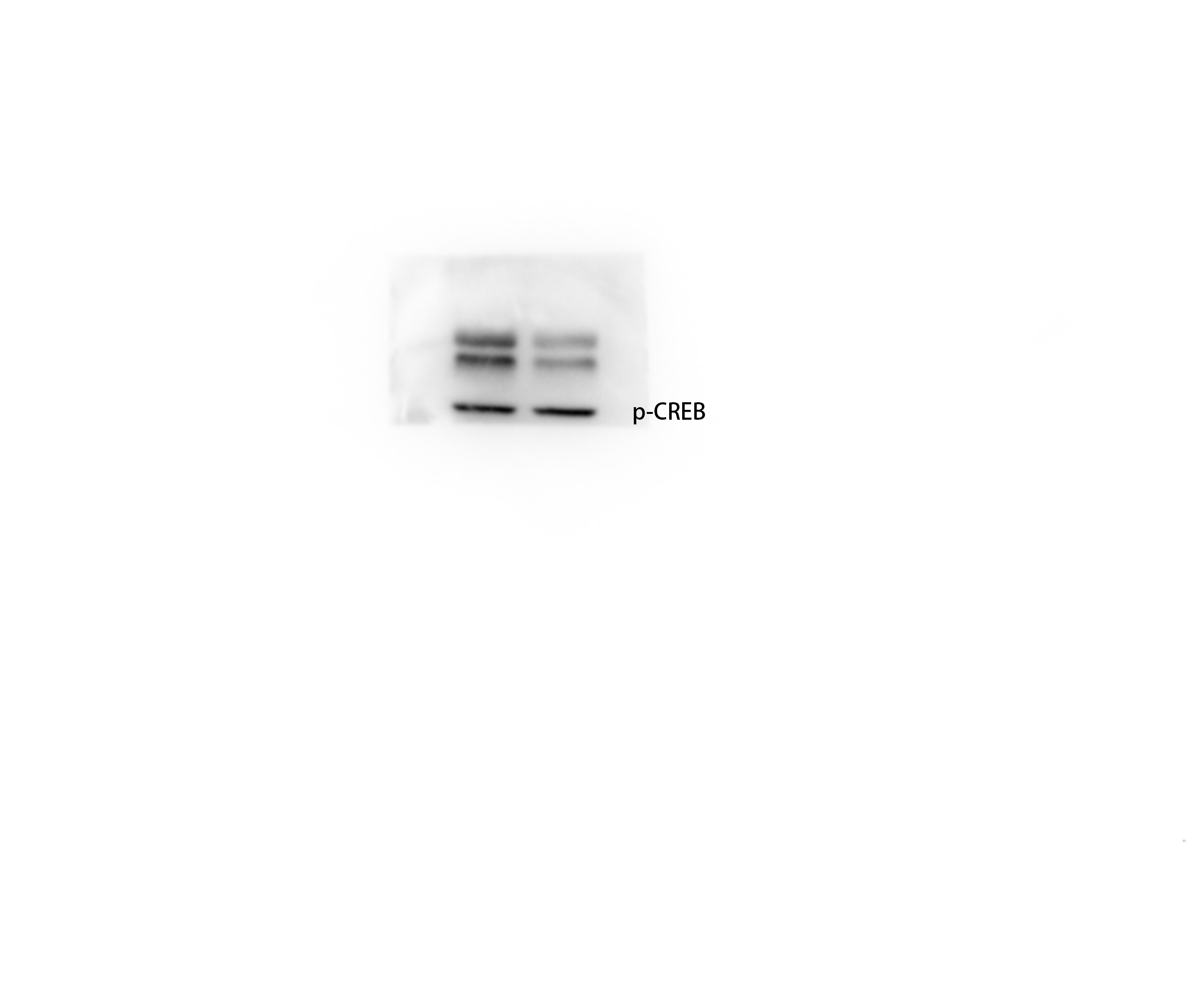

Supplement: Supplementary file 1 — Supplementary Material 1. [file 12964_2025_2550_MOESM1_ESM.zip › Sup_Figure 4A_pCREB.tif]

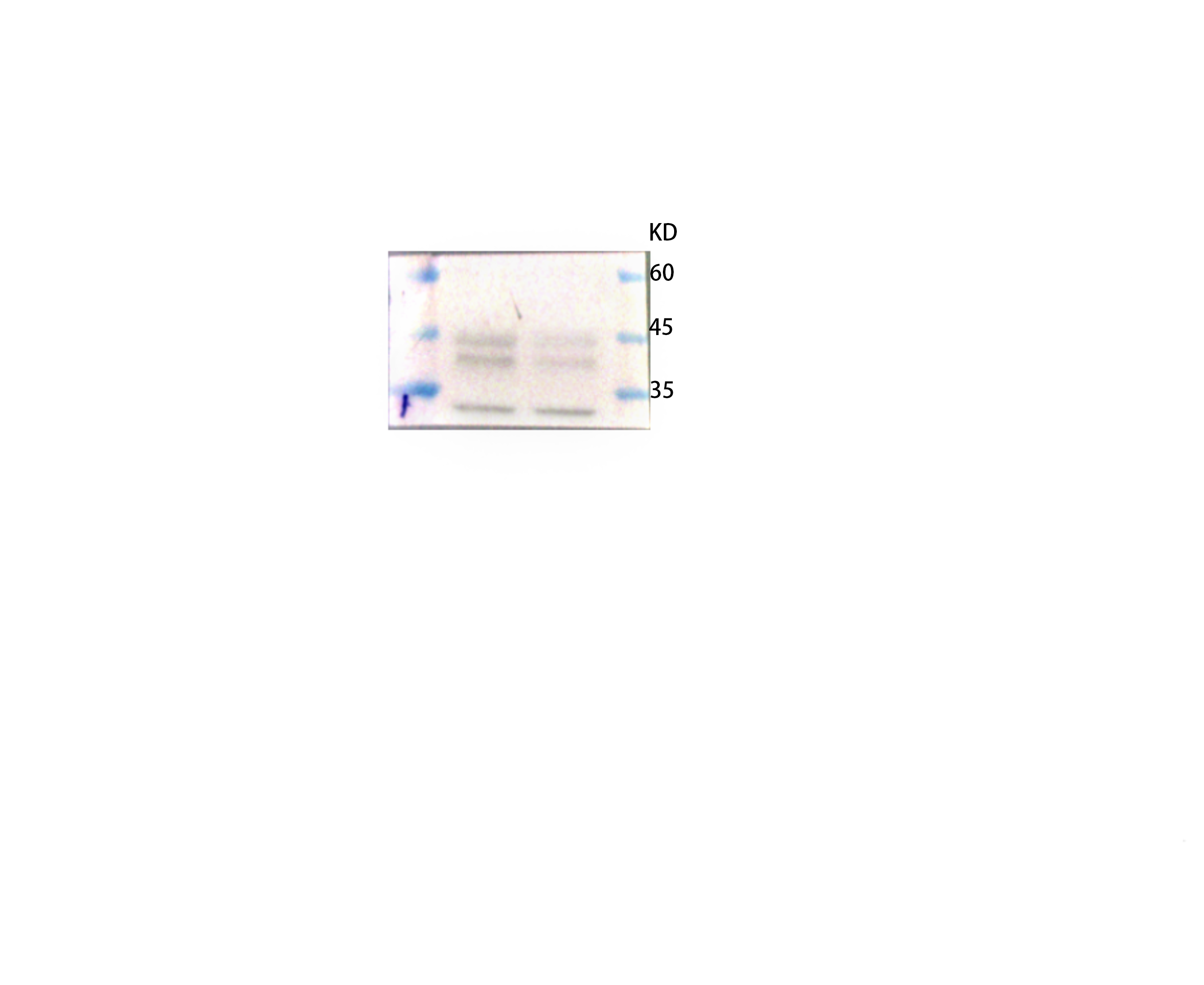

Supplement: Supplementary file 1 — Supplementary Material 1. [file 12964_2025_2550_MOESM1_ESM.zip › Sup_Figure 4A_pCREB+Marker.tif]

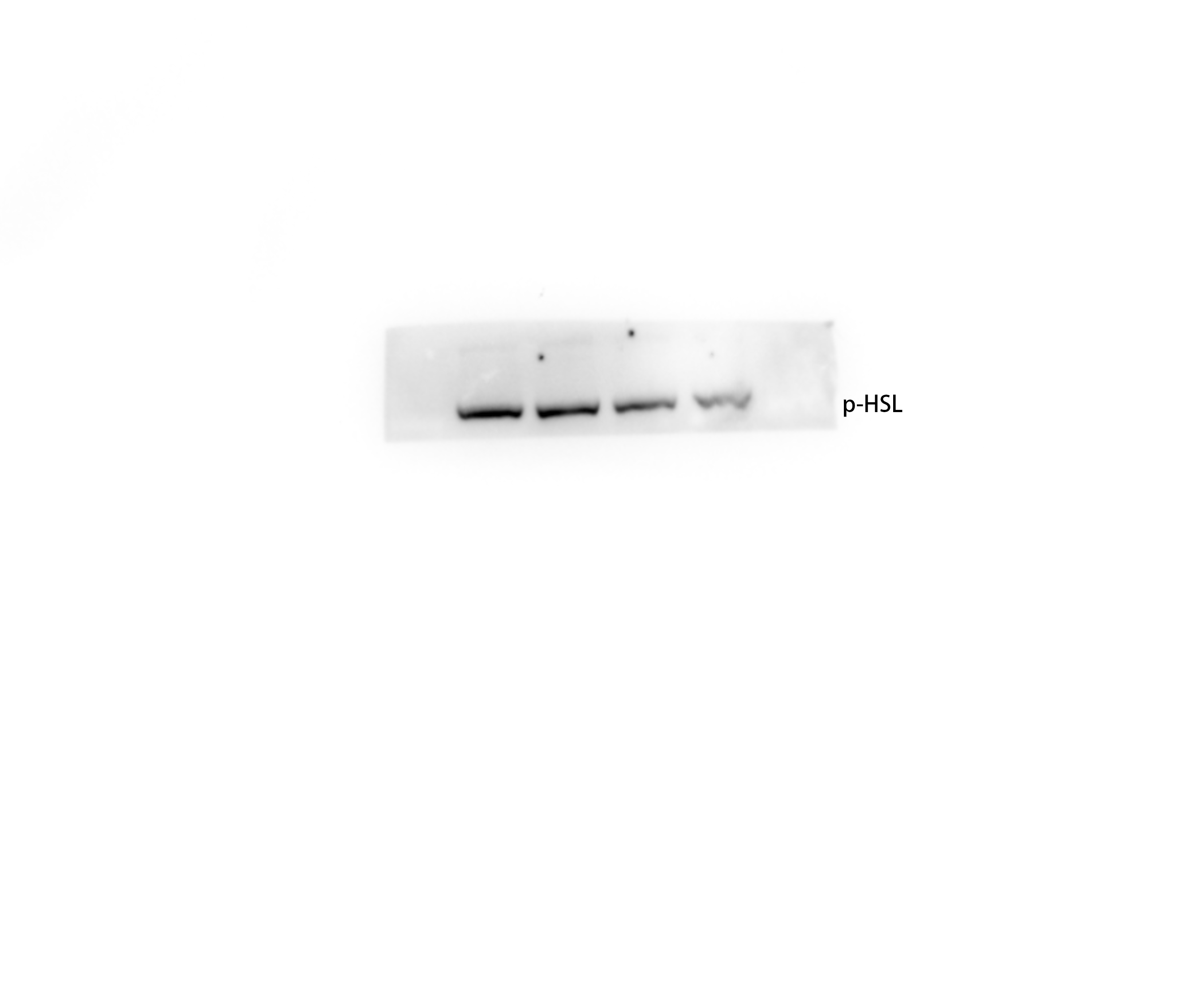

Supplement: Supplementary file 1 — Supplementary Material 1. [file 12964_2025_2550_MOESM1_ESM.zip › Sup_Figure 4A_pHSL.tif]

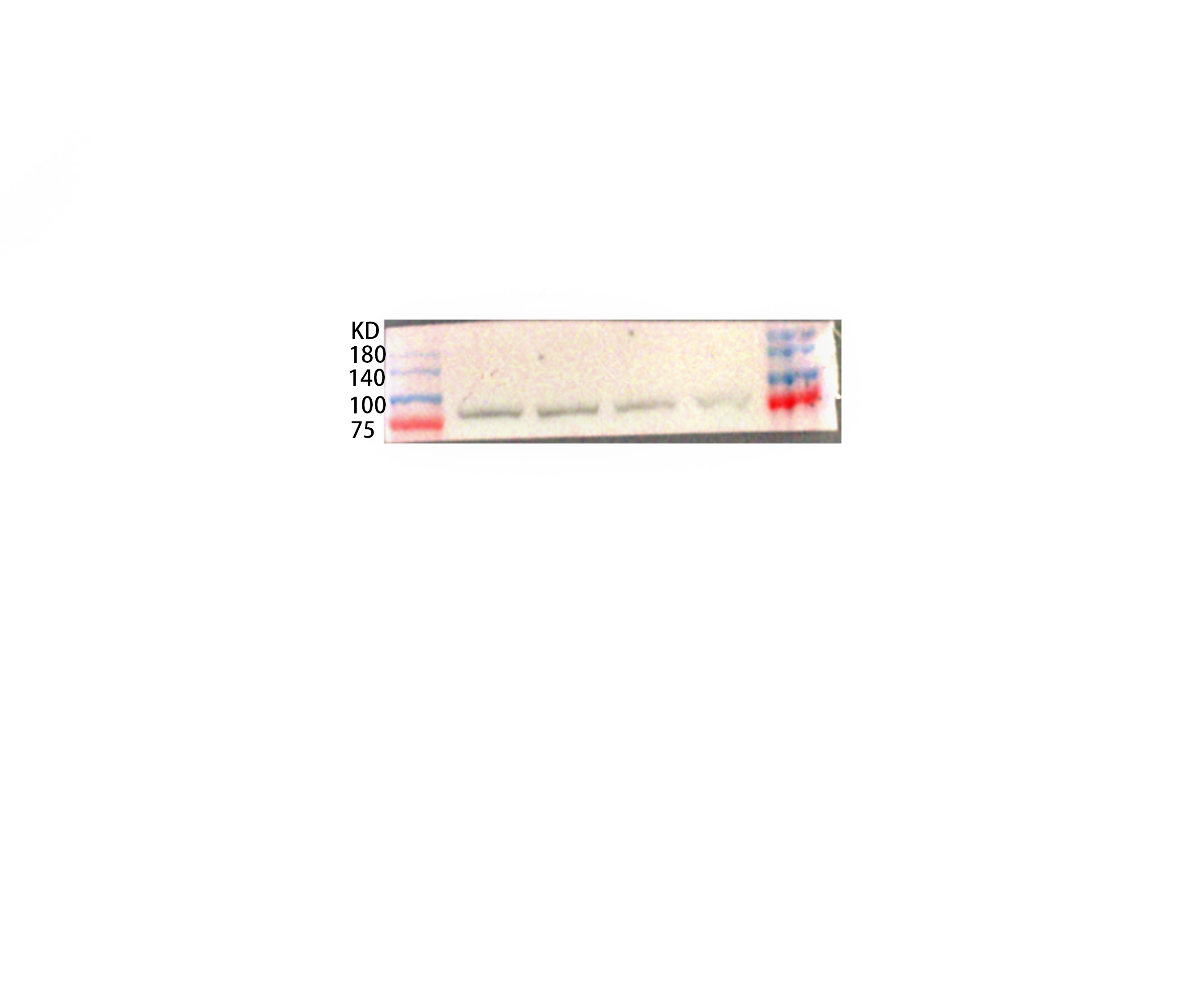

Supplement: Supplementary file 1 — Supplementary Material 1. [file 12964_2025_2550_MOESM1_ESM.zip › Sup_Figure 4A_pHSL+Marker.tif]

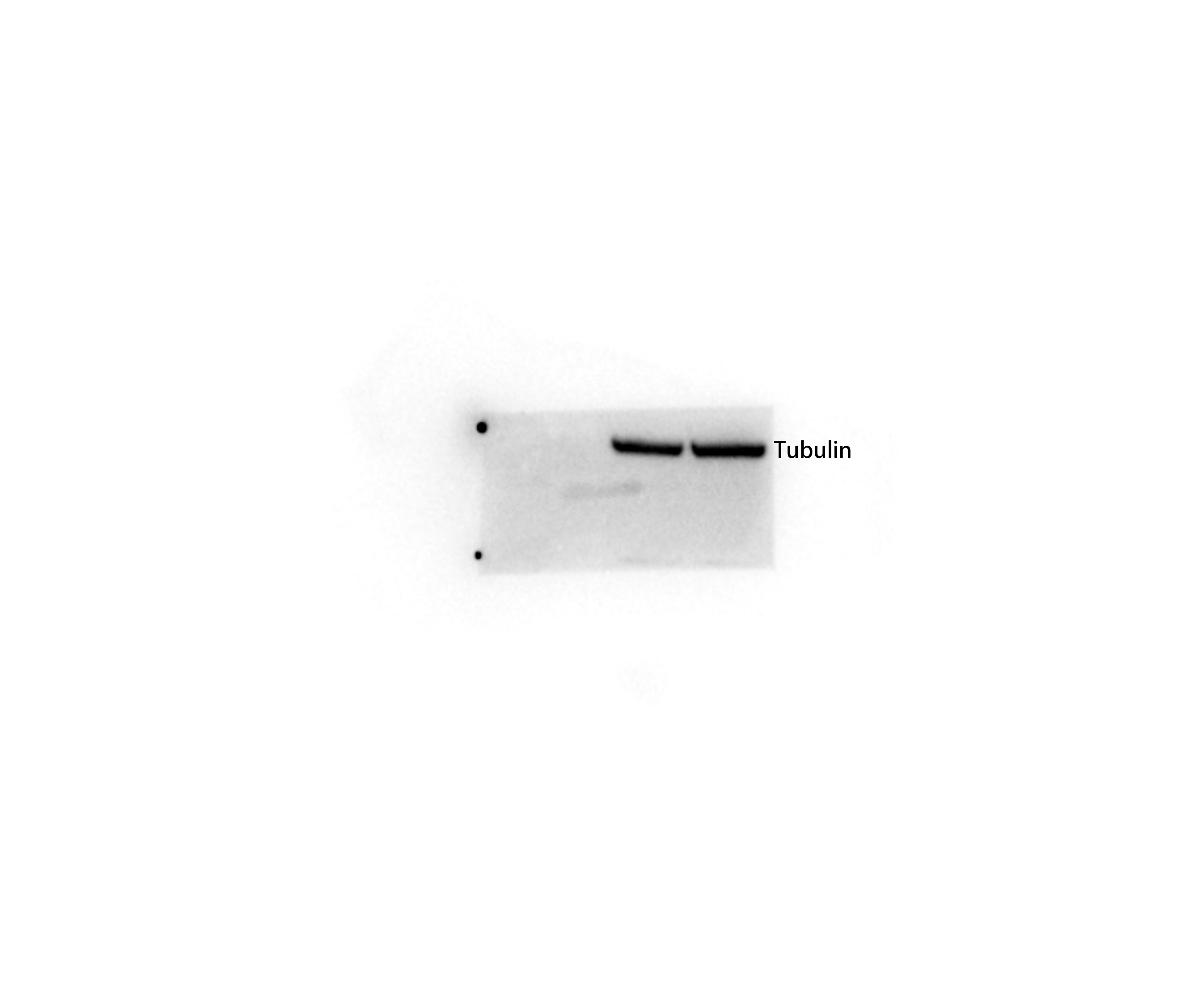

Supplement: Supplementary file 1 — Supplementary Material 1. [file 12964_2025_2550_MOESM1_ESM.zip › Sup_Figure 4A_tubulin.tif]

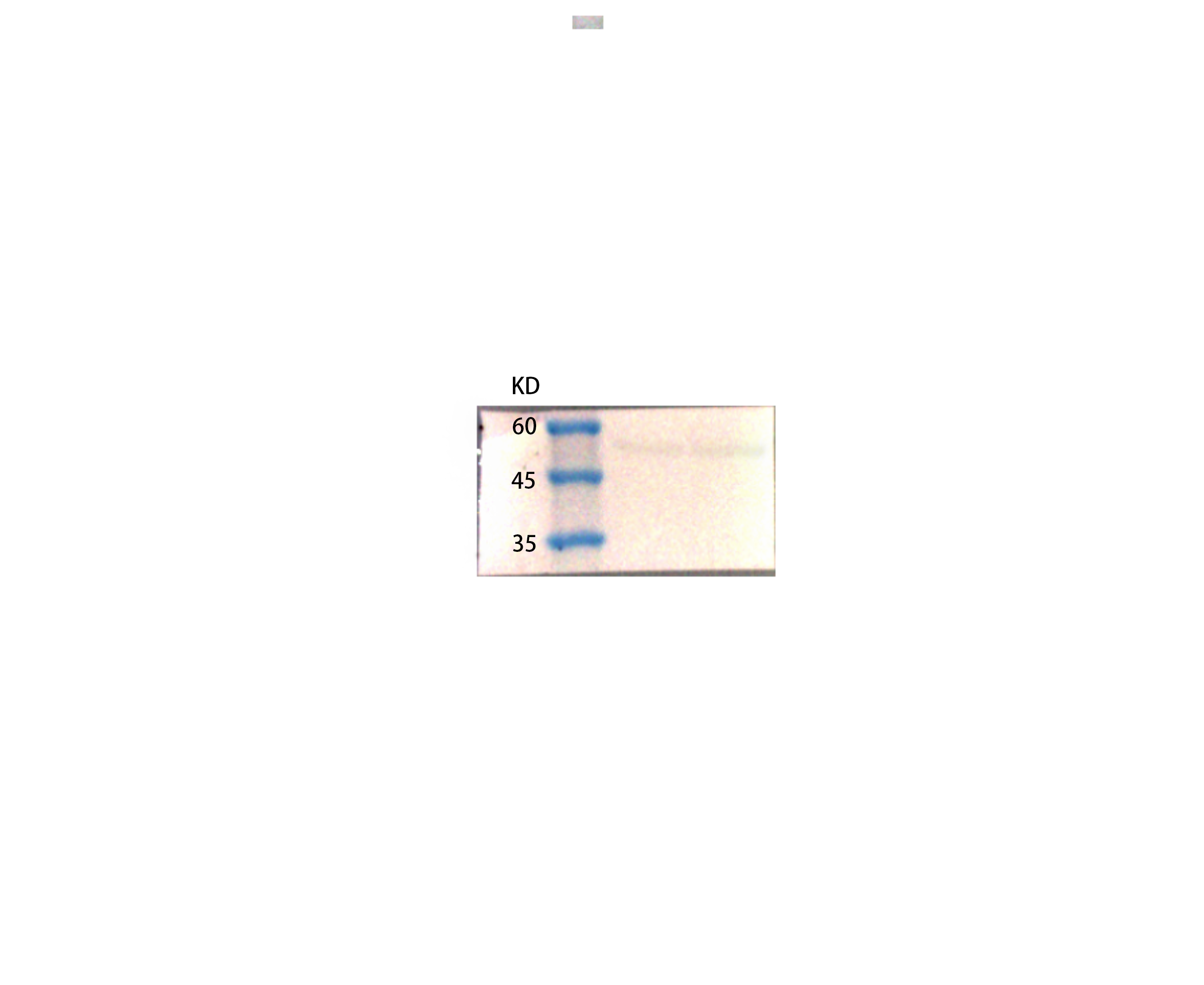

Supplement: Supplementary file 1 — Supplementary Material 1. [file 12964_2025_2550_MOESM1_ESM.zip › Sup_Figure 4A_tubulin+Marker.tif]

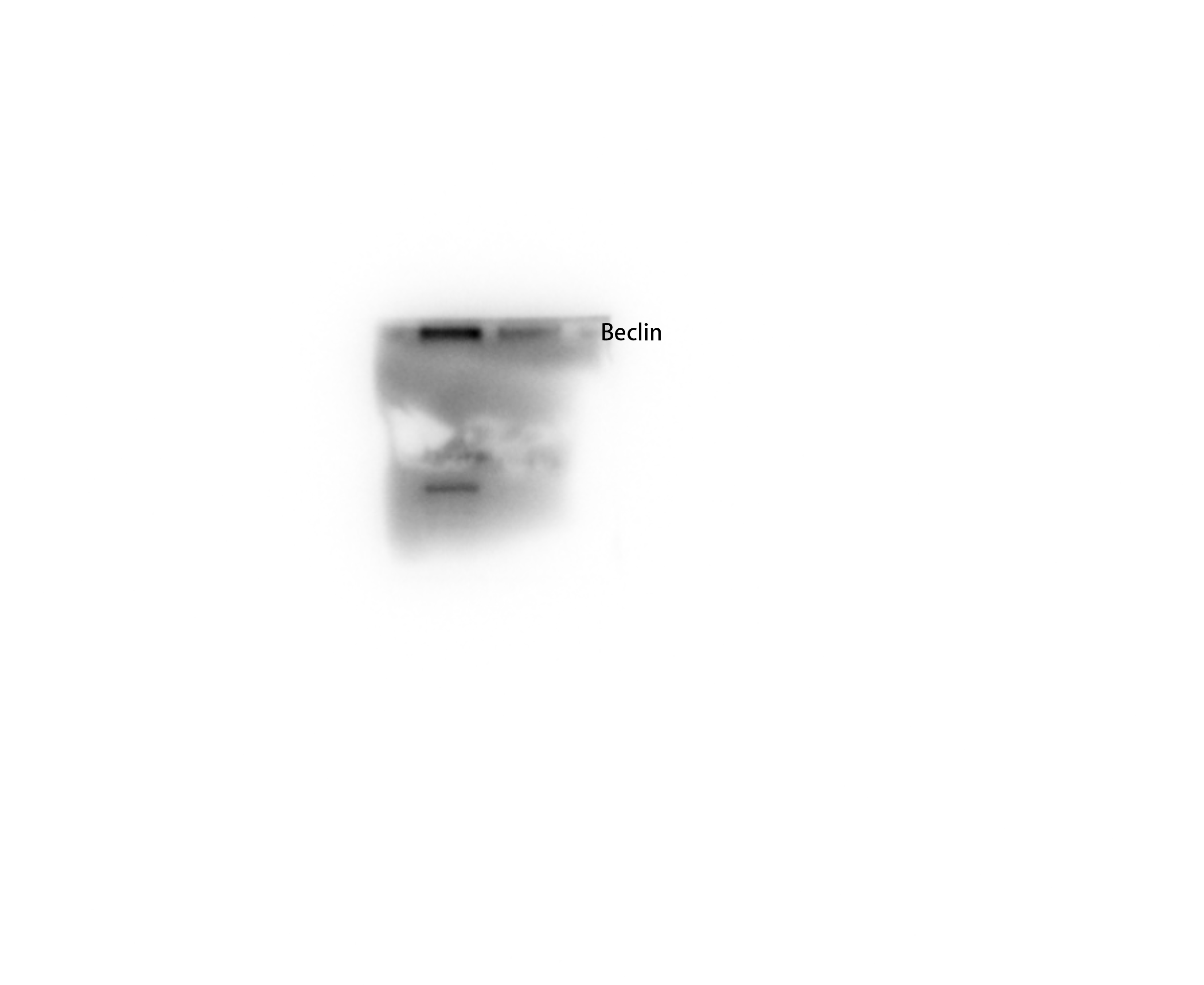

Supplement: Supplementary file 1 — Supplementary Material 1. [file 12964_2025_2550_MOESM1_ESM.zip › Sup_Figure 4B_Beclin.tif]

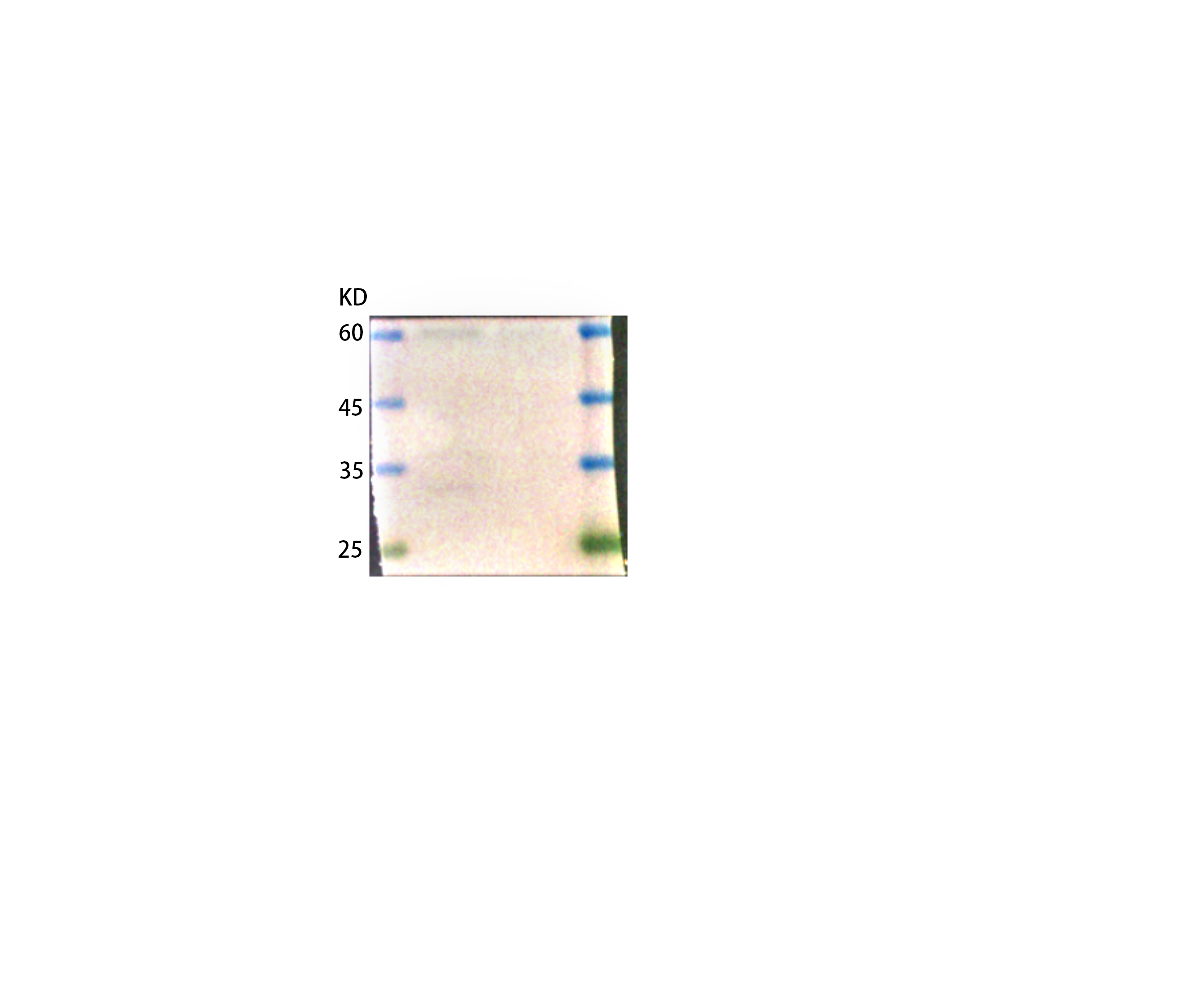

Supplement: Supplementary file 1 — Supplementary Material 1. [file 12964_2025_2550_MOESM1_ESM.zip › Sup_Figure 4B_Beclin+Marker.tif]

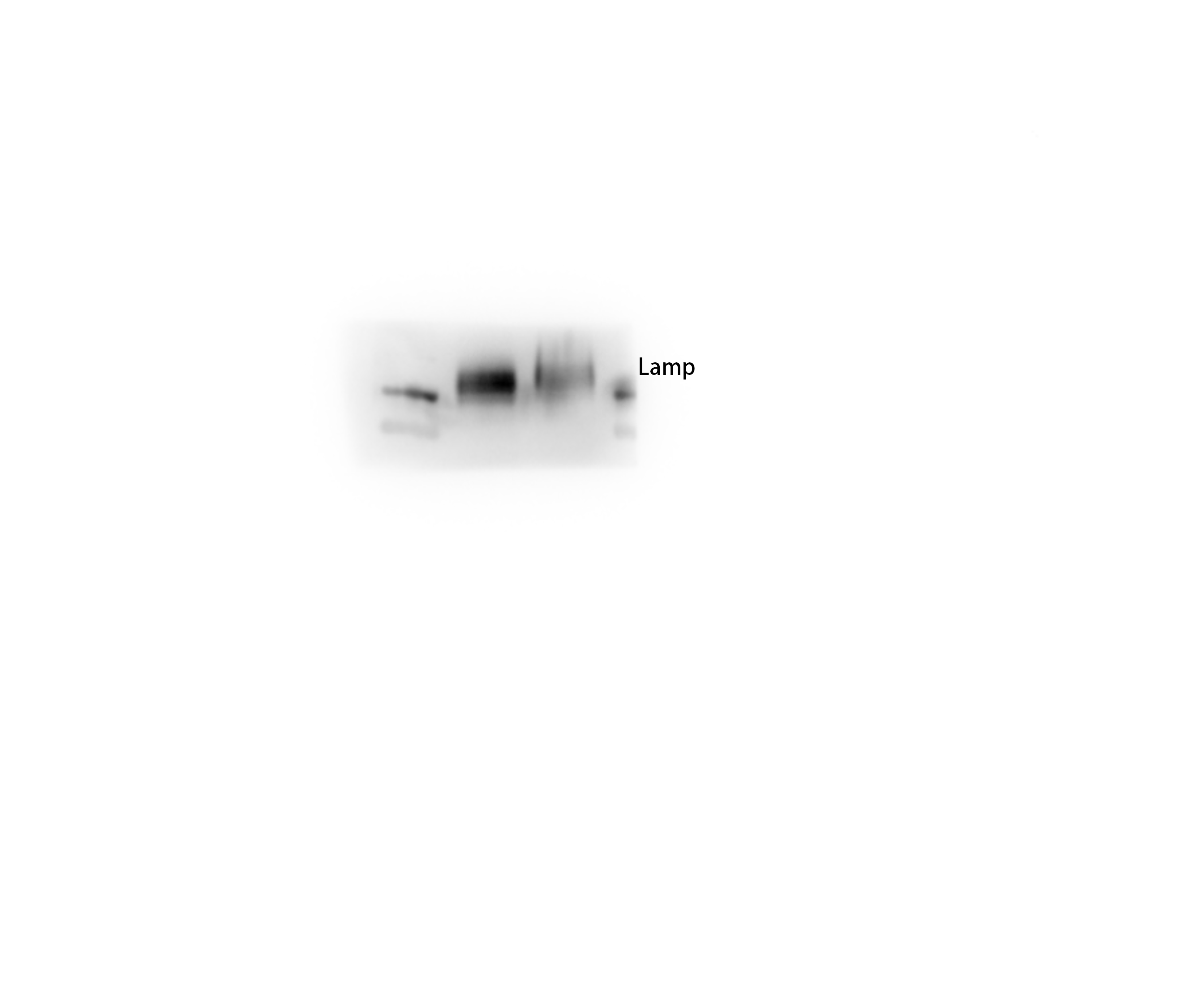

Supplement: Supplementary file 1 — Supplementary Material 1. [file 12964_2025_2550_MOESM1_ESM.zip › Sup_Figure 4B_Lamp.tif]

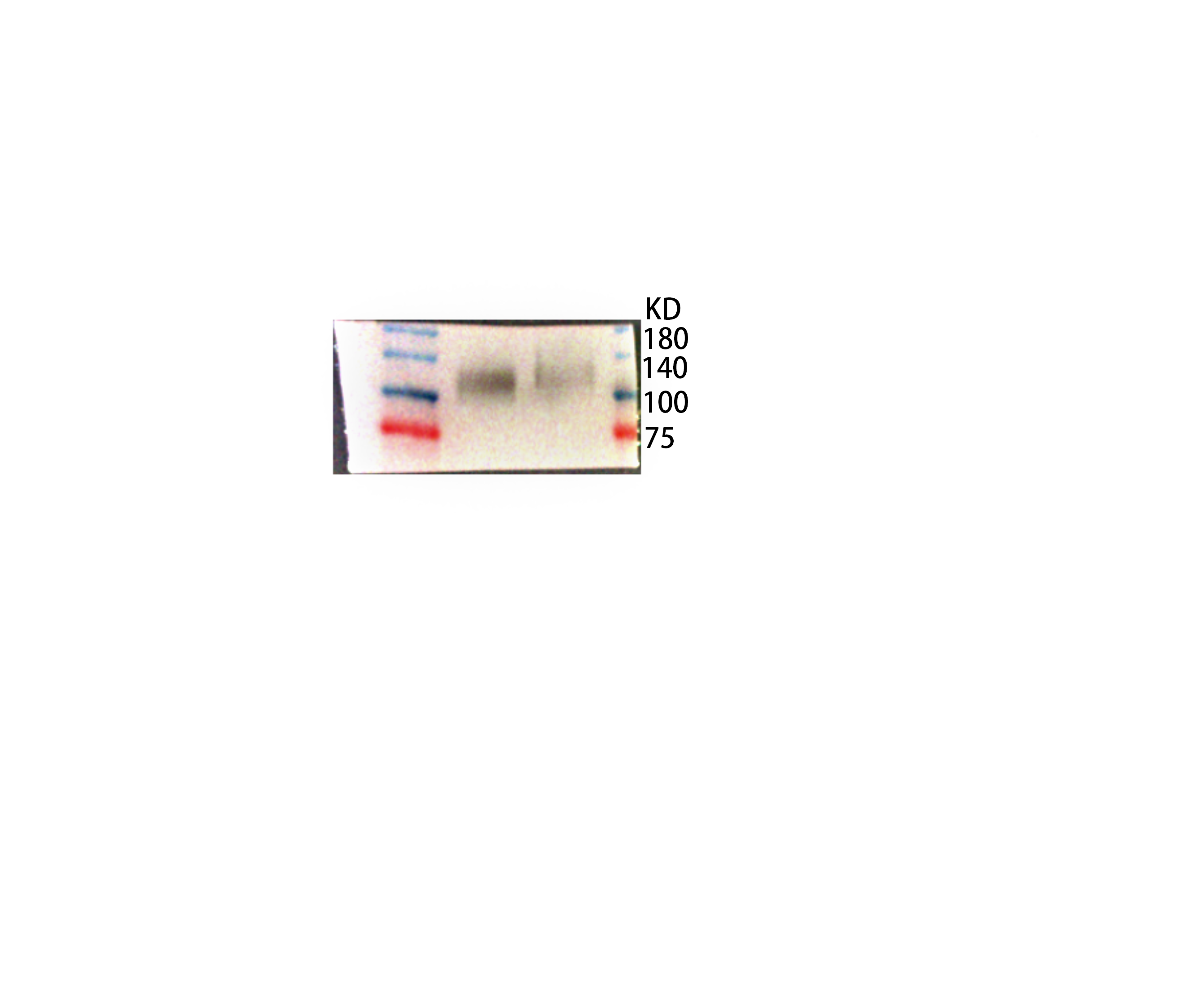

Supplement: Supplementary file 1 — Supplementary Material 1. [file 12964_2025_2550_MOESM1_ESM.zip › Sup_Figure 4B_Lamp+Marker.tif]

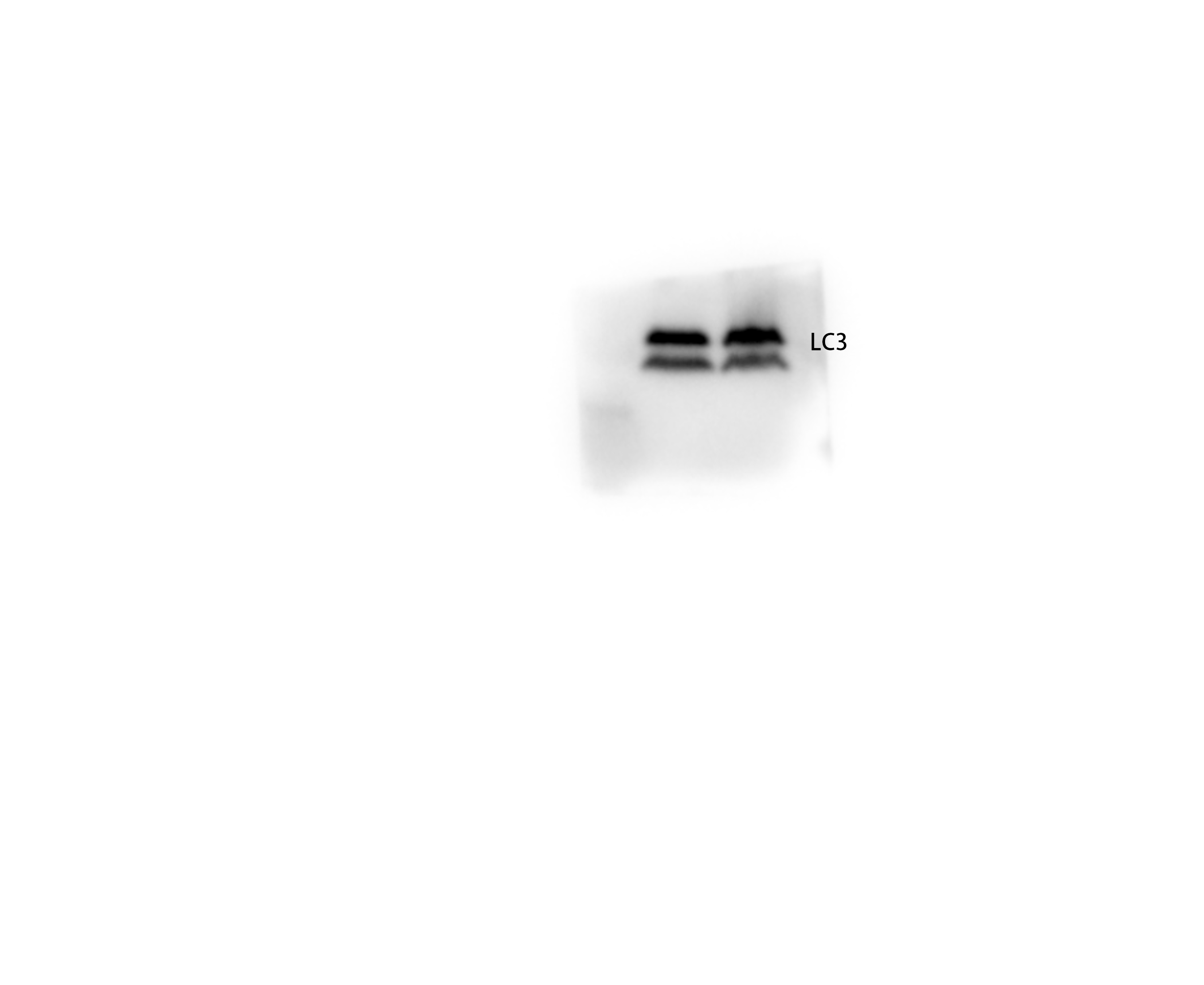

Supplement: Supplementary file 1 — Supplementary Material 1. [file 12964_2025_2550_MOESM1_ESM.zip › Sup_Figure 4B_LC3.tif]

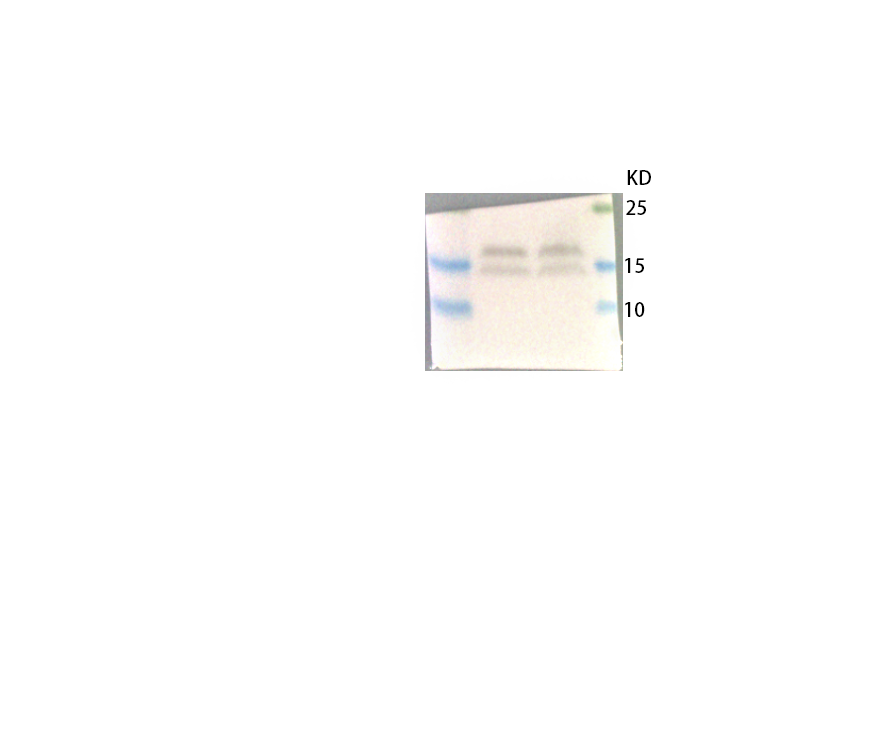

Supplement: Supplementary file 1 — Supplementary Material 1. [file 12964_2025_2550_MOESM1_ESM.zip › Sup_Figure 4B_LC3+Marker.tif]

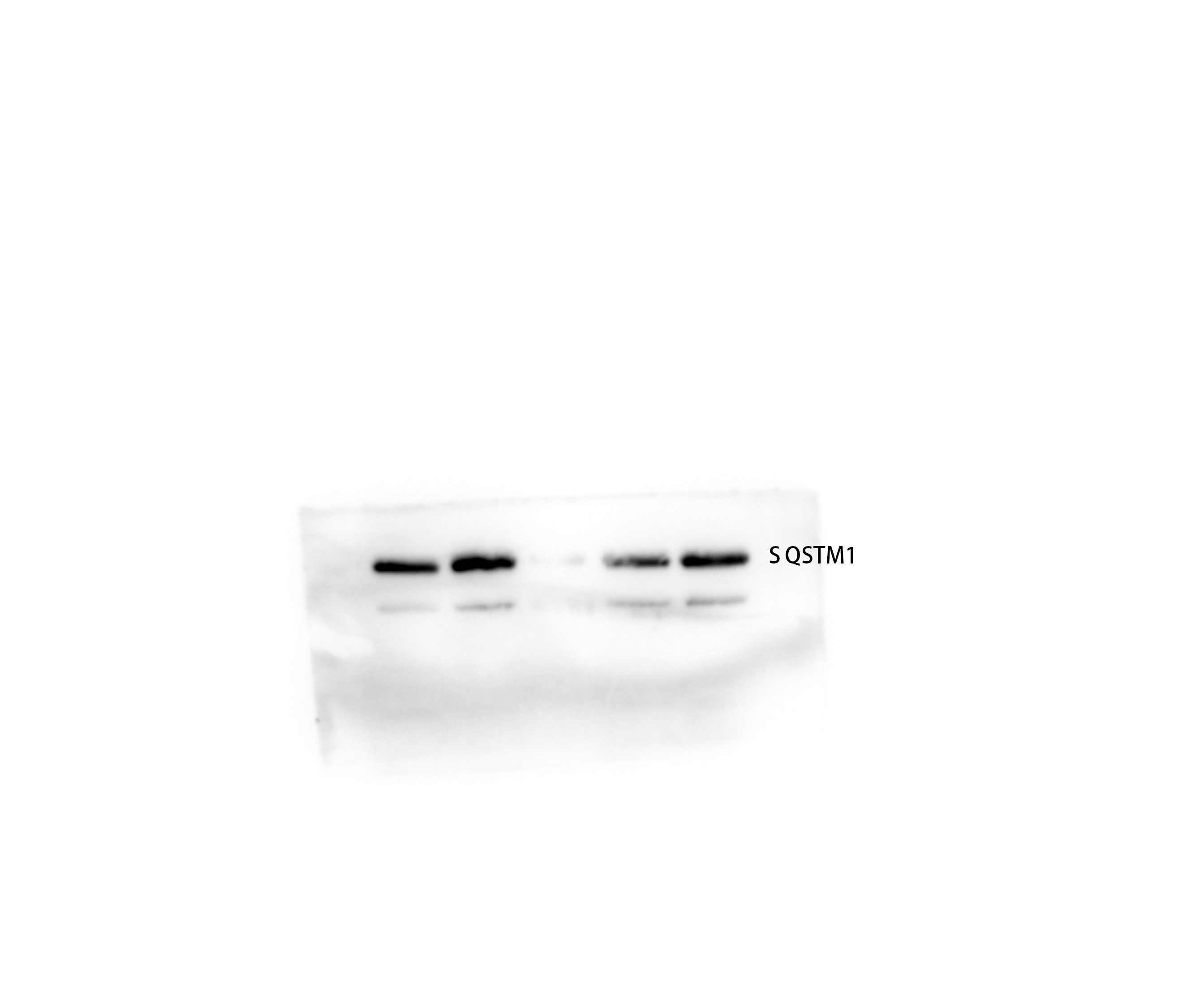

Supplement: Supplementary file 1 — Supplementary Material 1. [file 12964_2025_2550_MOESM1_ESM.zip › Sup_Figure 4B_SQSTM1.tif]

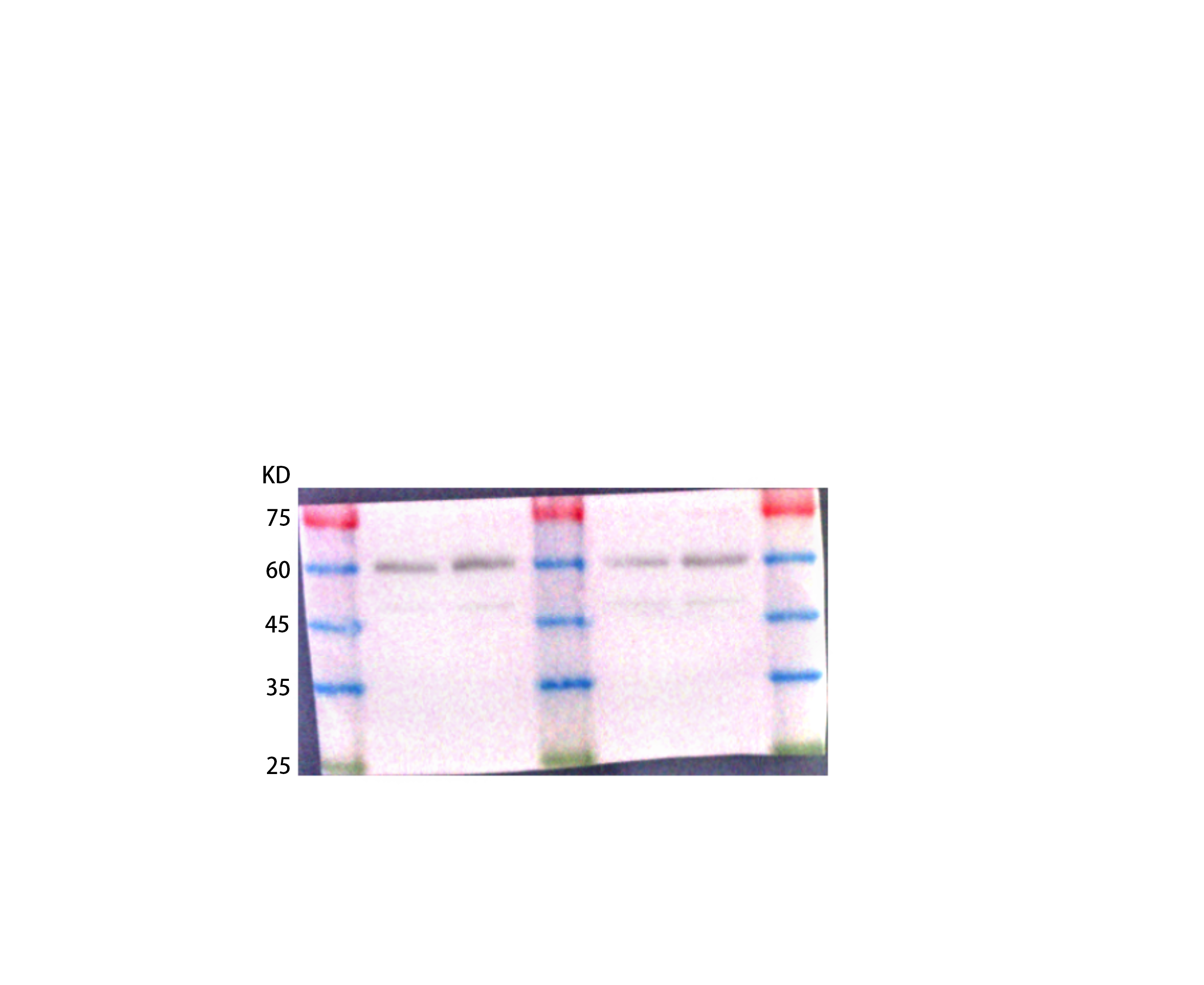

Supplement: Supplementary file 1 — Supplementary Material 1. [file 12964_2025_2550_MOESM1_ESM.zip › Sup_Figure 4B_SQSTM1+marker.tif]

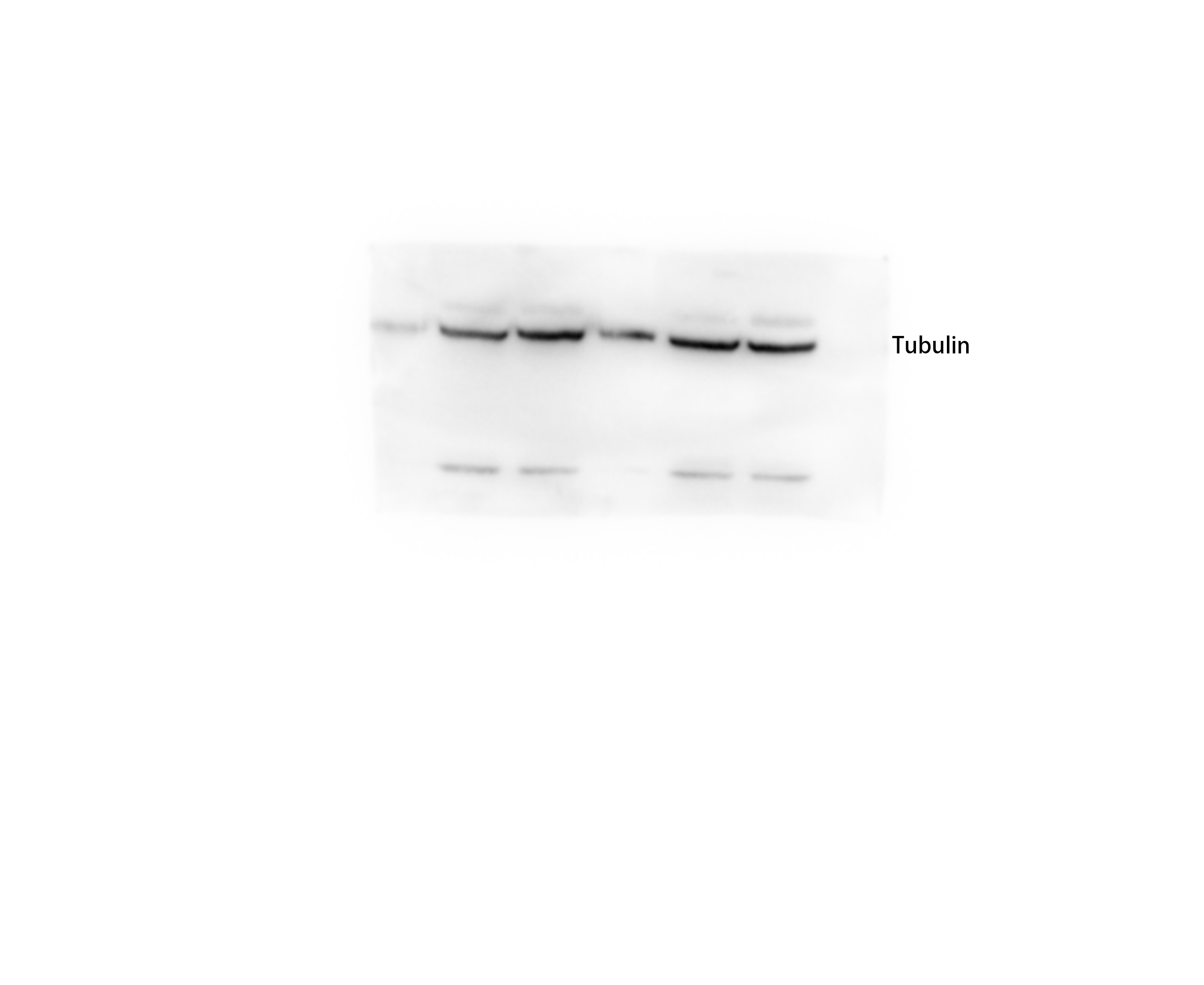

Supplement: Supplementary file 1 — Supplementary Material 1. [file 12964_2025_2550_MOESM1_ESM.zip › Sup_Figure 4B_Tubulin.tif]

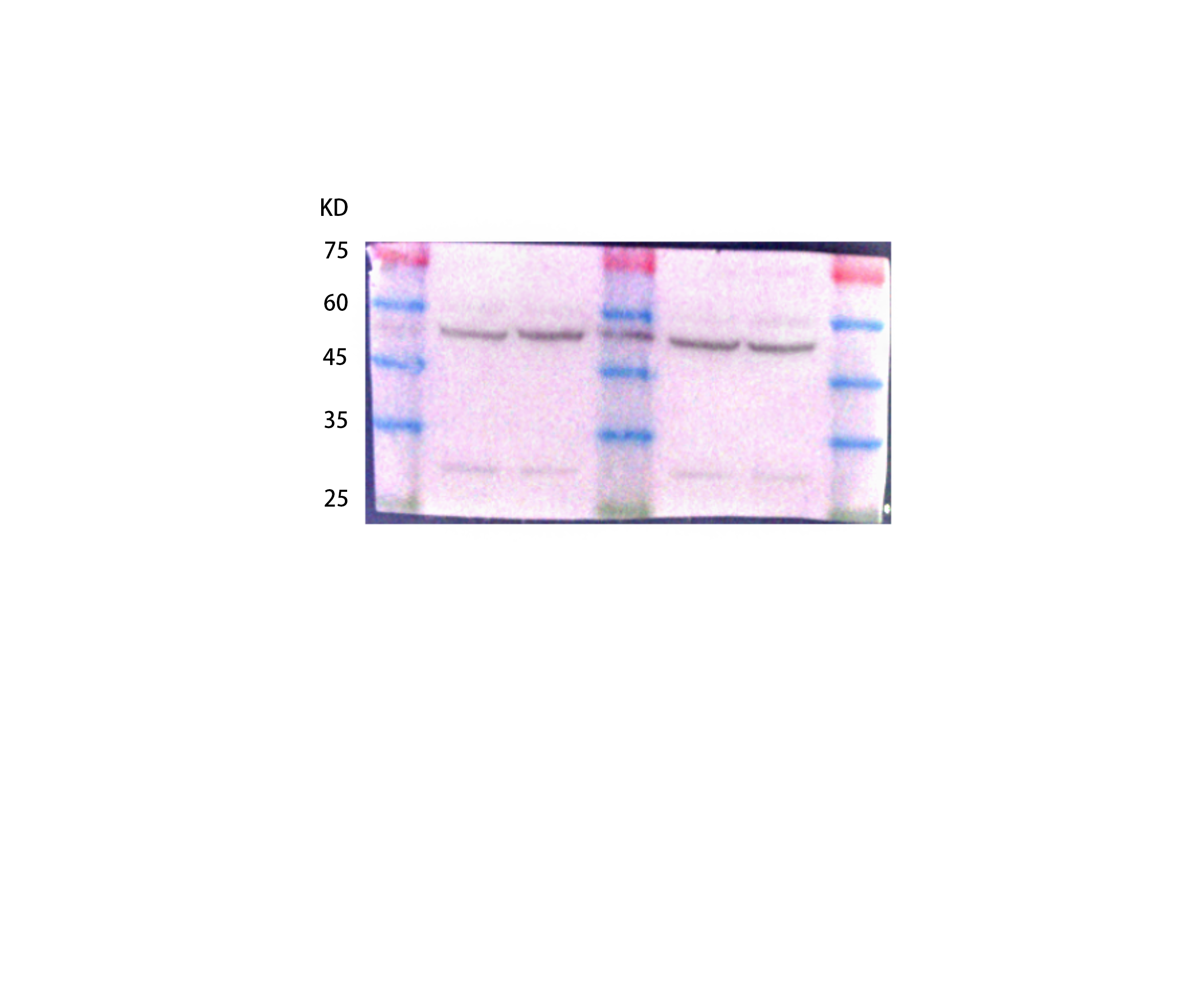

Supplement: Supplementary file 1 — Supplementary Material 1. [file 12964_2025_2550_MOESM1_ESM.zip › Sup_Figure 4B_Tubulin+marker.tif]

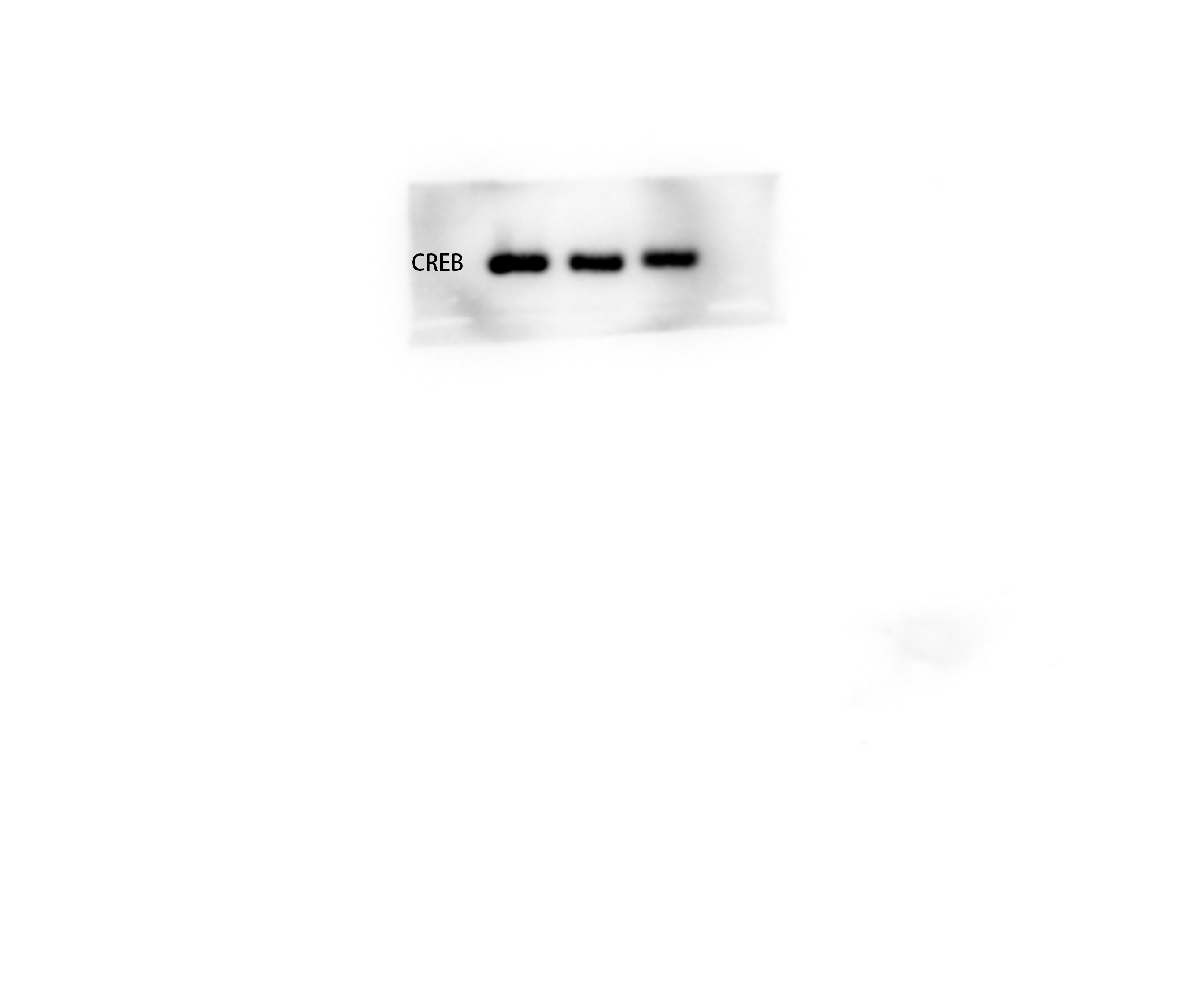

Supplement: Supplementary file 1 — Supplementary Material 1. [file 12964_2025_2550_MOESM1_ESM.zip › Sup_Figure 5G_CREB.tif]

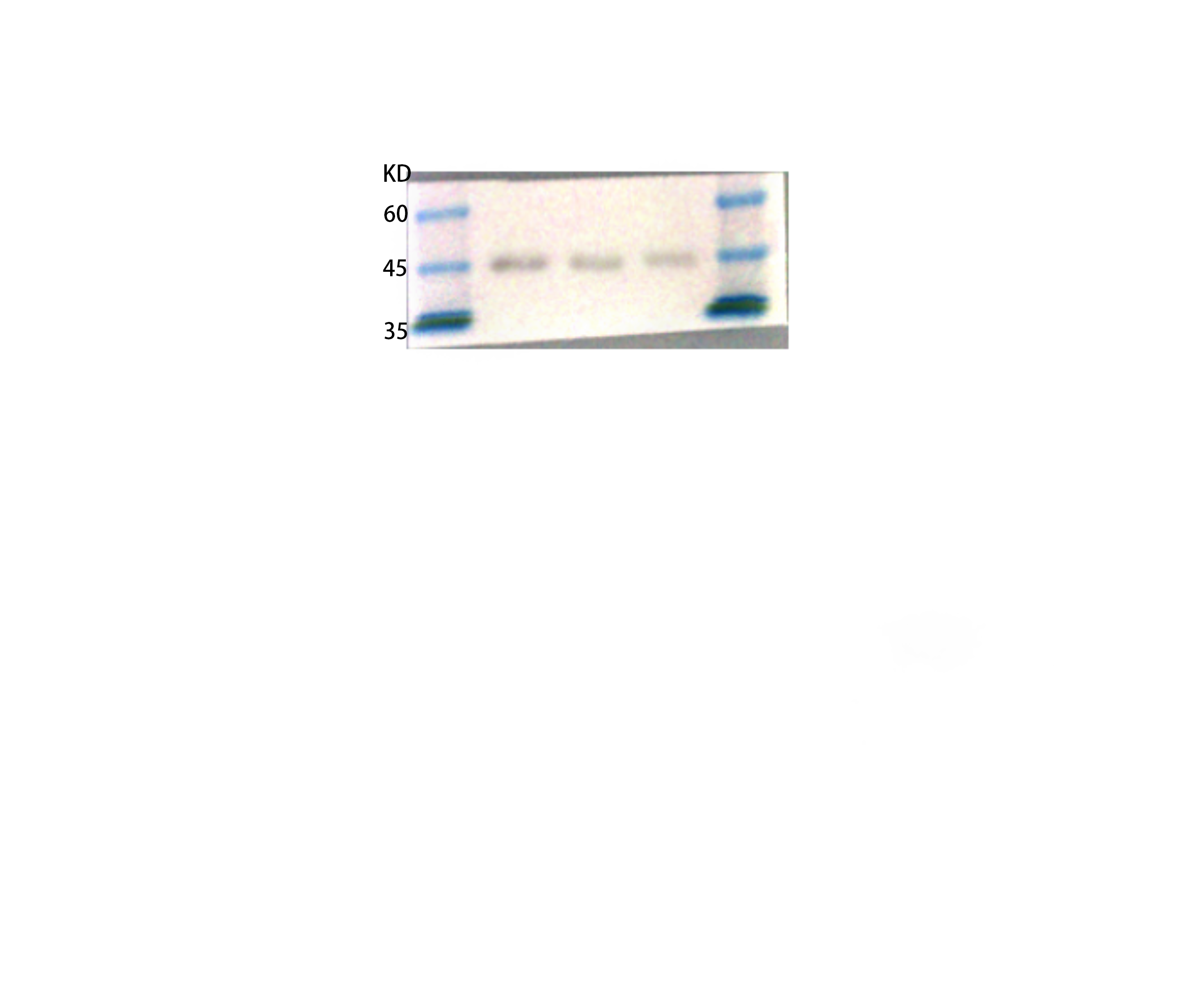

Supplement: Supplementary file 1 — Supplementary Material 1. [file 12964_2025_2550_MOESM1_ESM.zip › Sup_Figure 5G_CREB+Marker.tif]

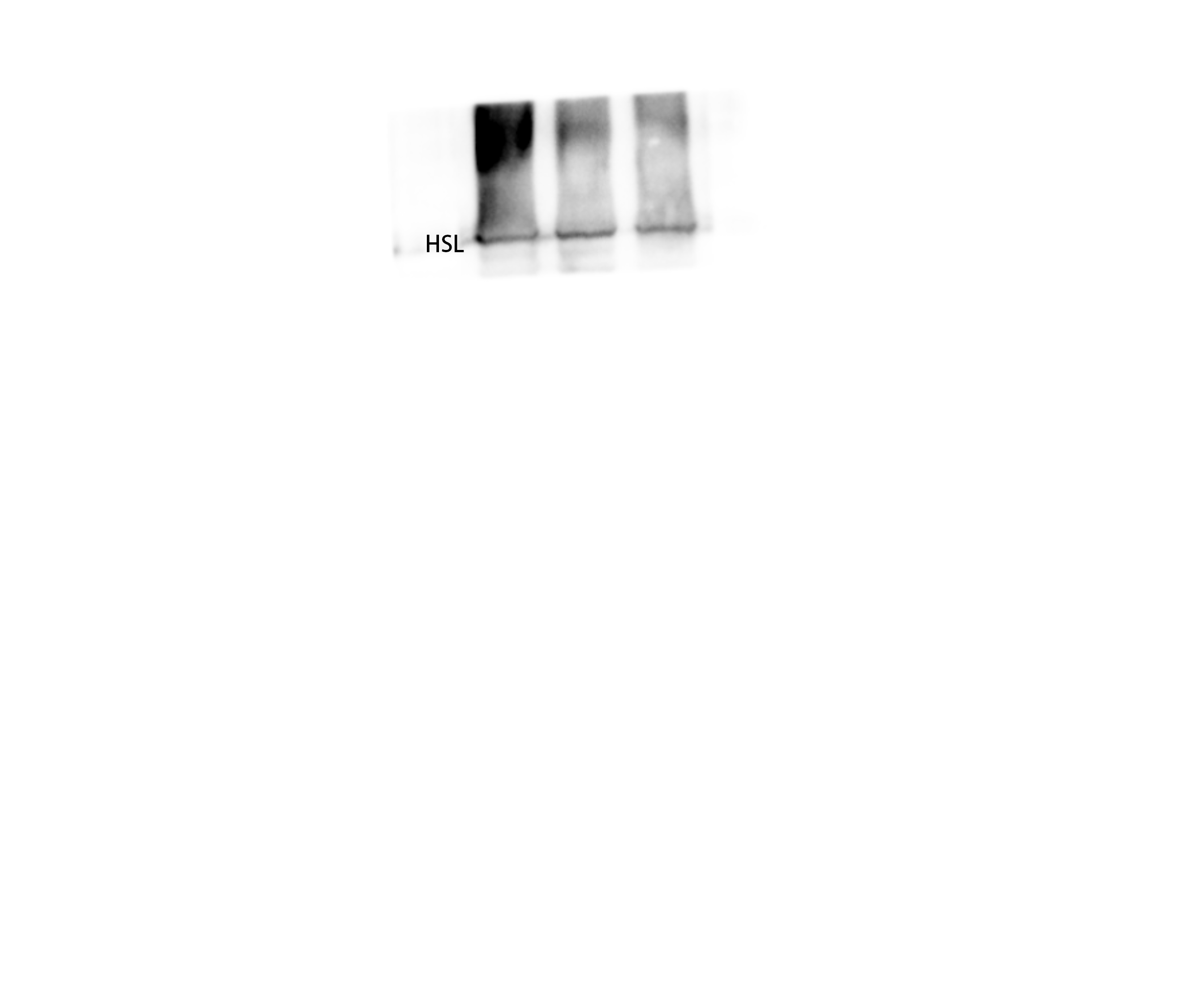

Supplement: Supplementary file 1 — Supplementary Material 1. [file 12964_2025_2550_MOESM1_ESM.zip › Sup_Figure 5G_HSL.tif]

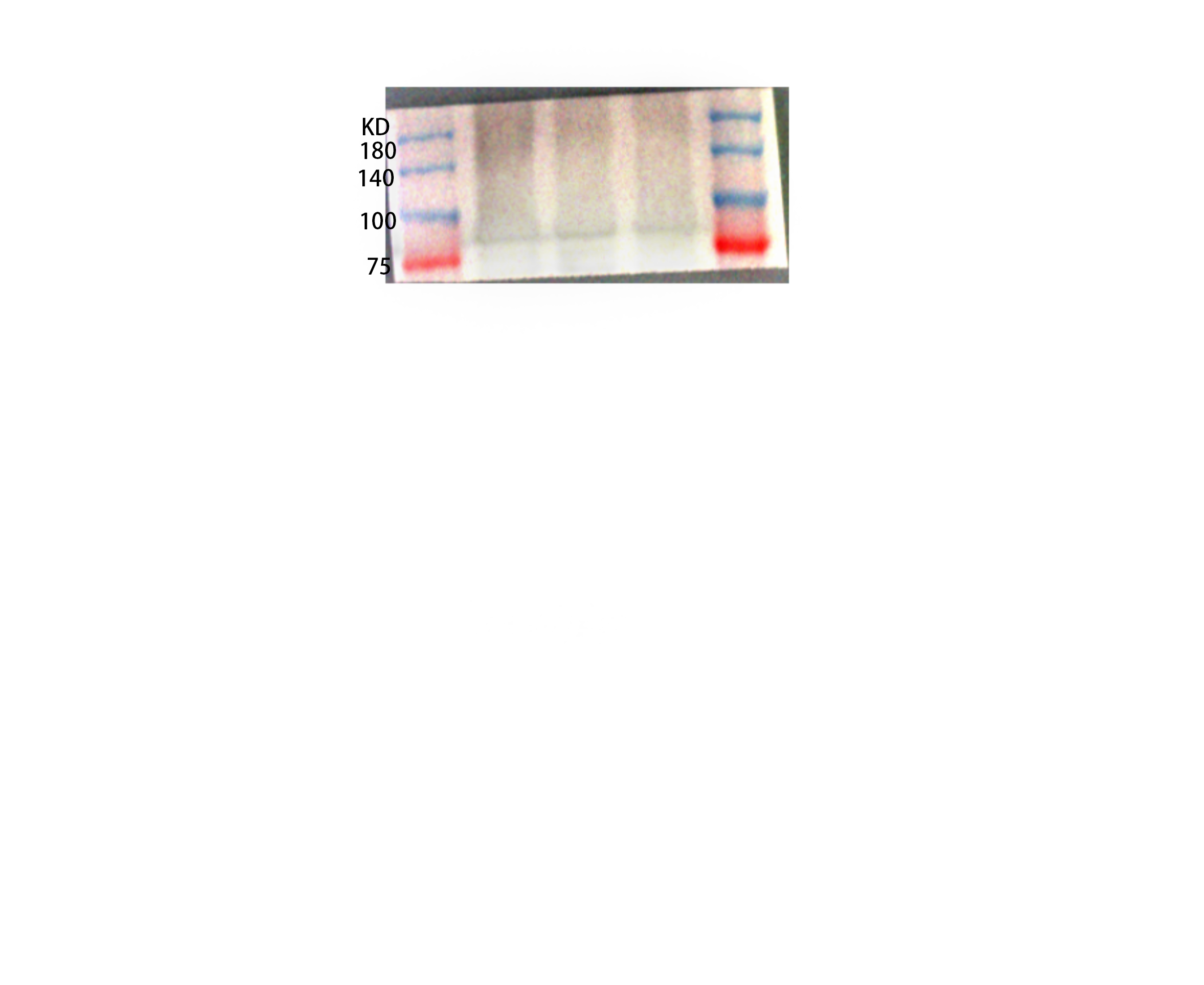

Supplement: Supplementary file 1 — Supplementary Material 1. [file 12964_2025_2550_MOESM1_ESM.zip › Sup_Figure 5G_HSL+Marker.tif]

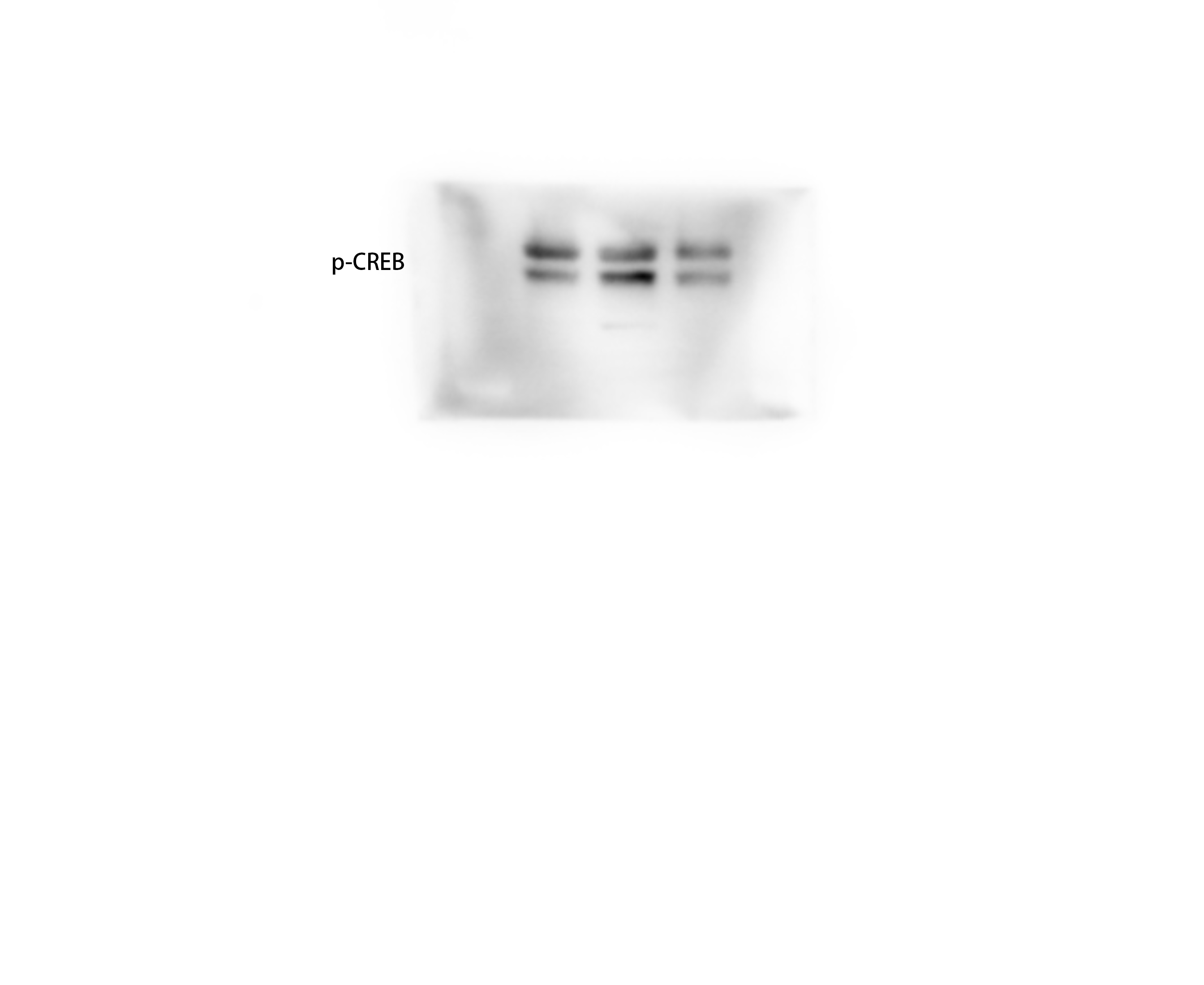

Supplement: Supplementary file 1 — Supplementary Material 1. [file 12964_2025_2550_MOESM1_ESM.zip › Sup_Figure 5G_p-CREB.tif]

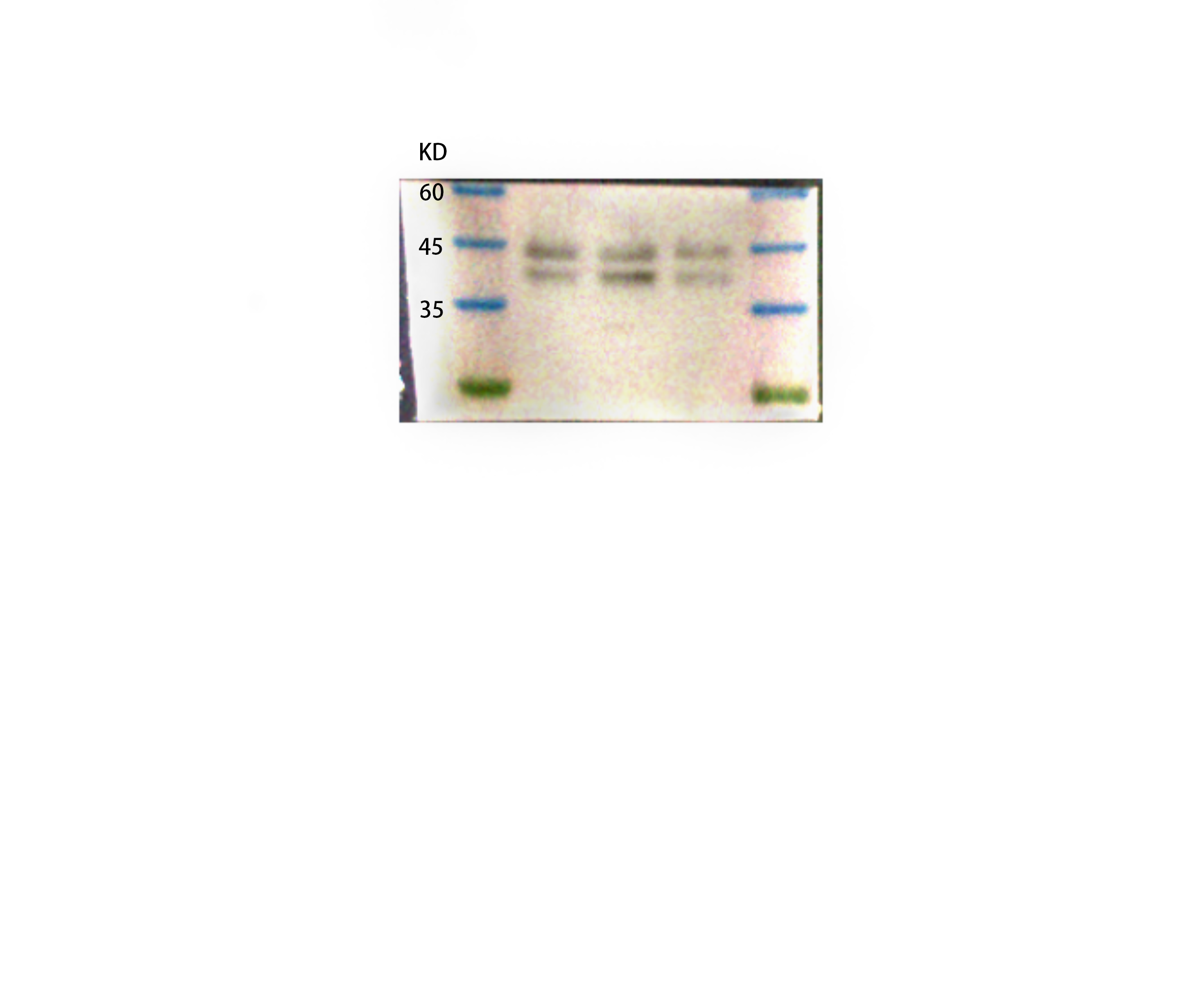

Supplement: Supplementary file 1 — Supplementary Material 1. [file 12964_2025_2550_MOESM1_ESM.zip › Sup_Figure 5G_p-CREB+Marker.tif]

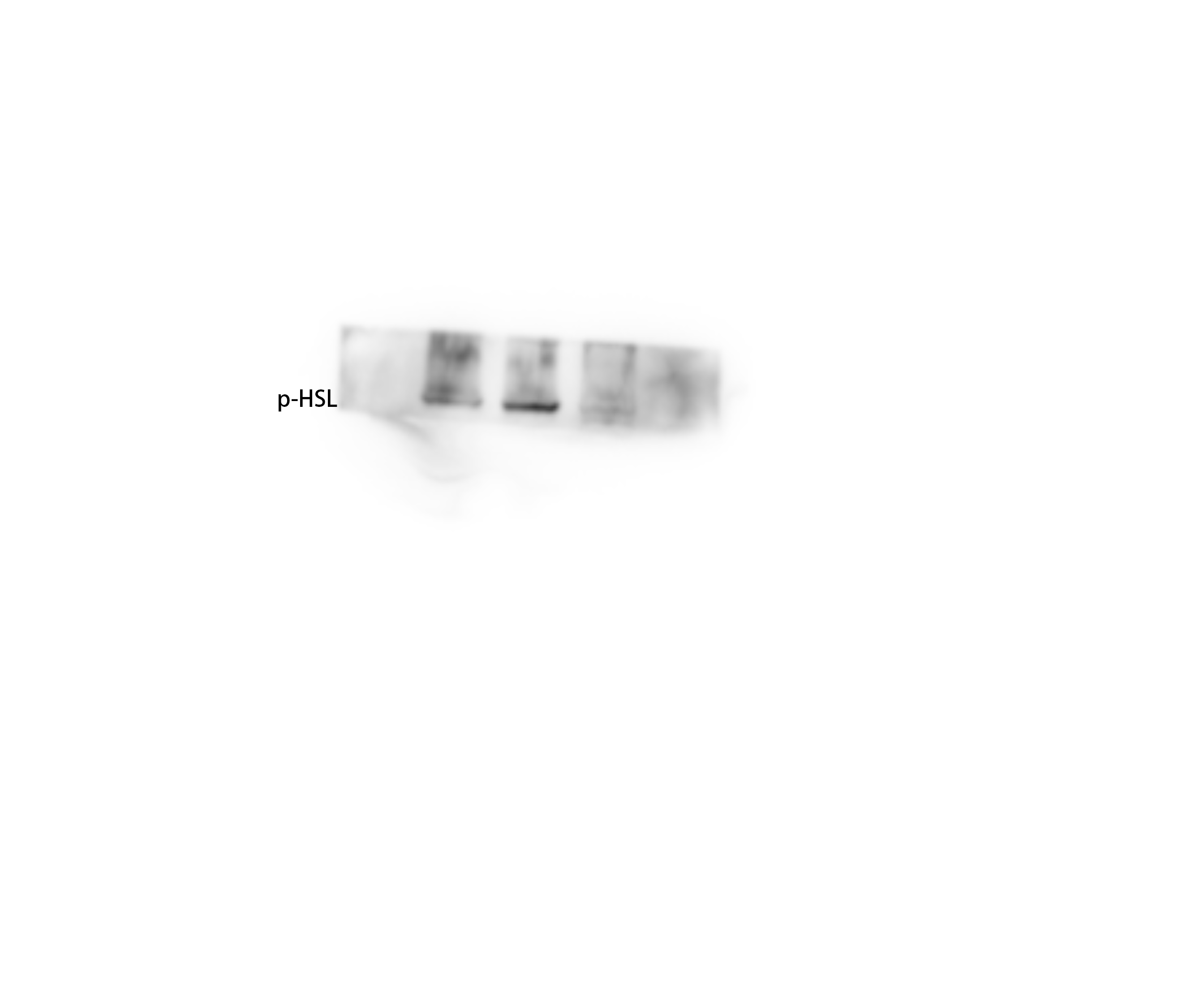

Supplement: Supplementary file 1 — Supplementary Material 1. [file 12964_2025_2550_MOESM1_ESM.zip › Sup_Figure 5G_p-HSL.tif]

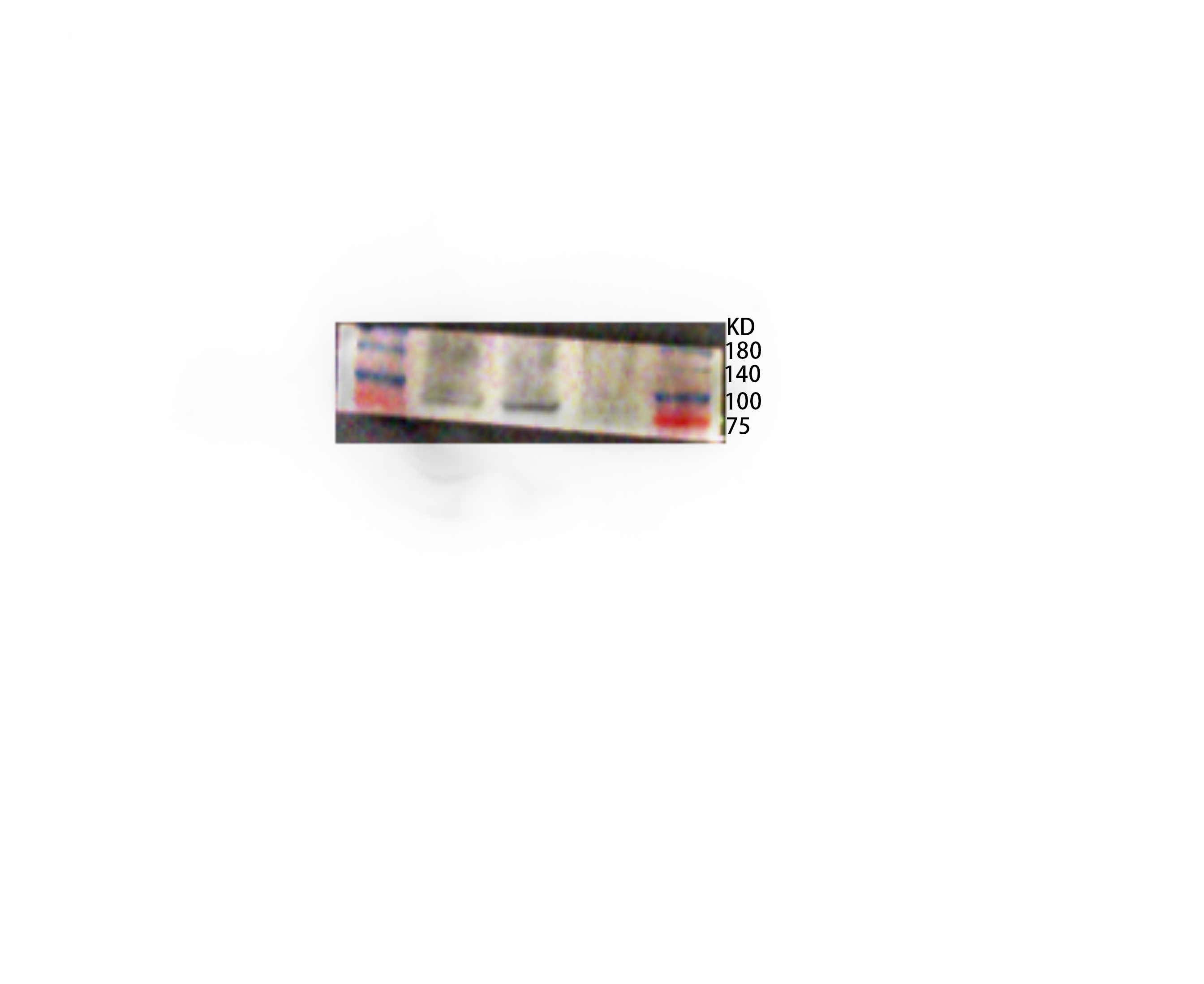

Supplement: Supplementary file 1 — Supplementary Material 1. [file 12964_2025_2550_MOESM1_ESM.zip › Sup_Figure 5G_p-HSL+Marker.tif]

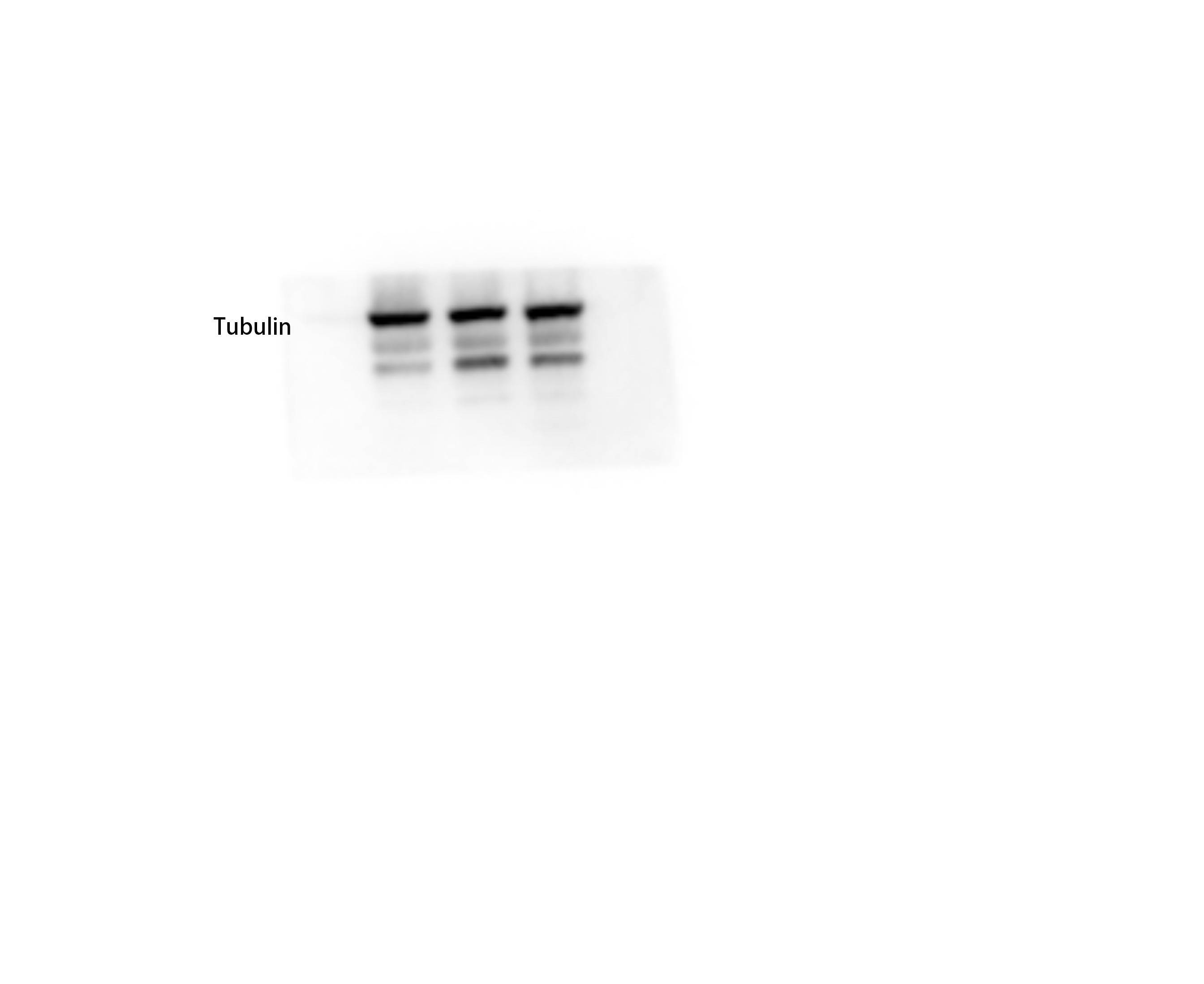

Supplement: Supplementary file 1 — Supplementary Material 1. [file 12964_2025_2550_MOESM1_ESM.zip › Sup_Figure 5G_tubulin.tif]

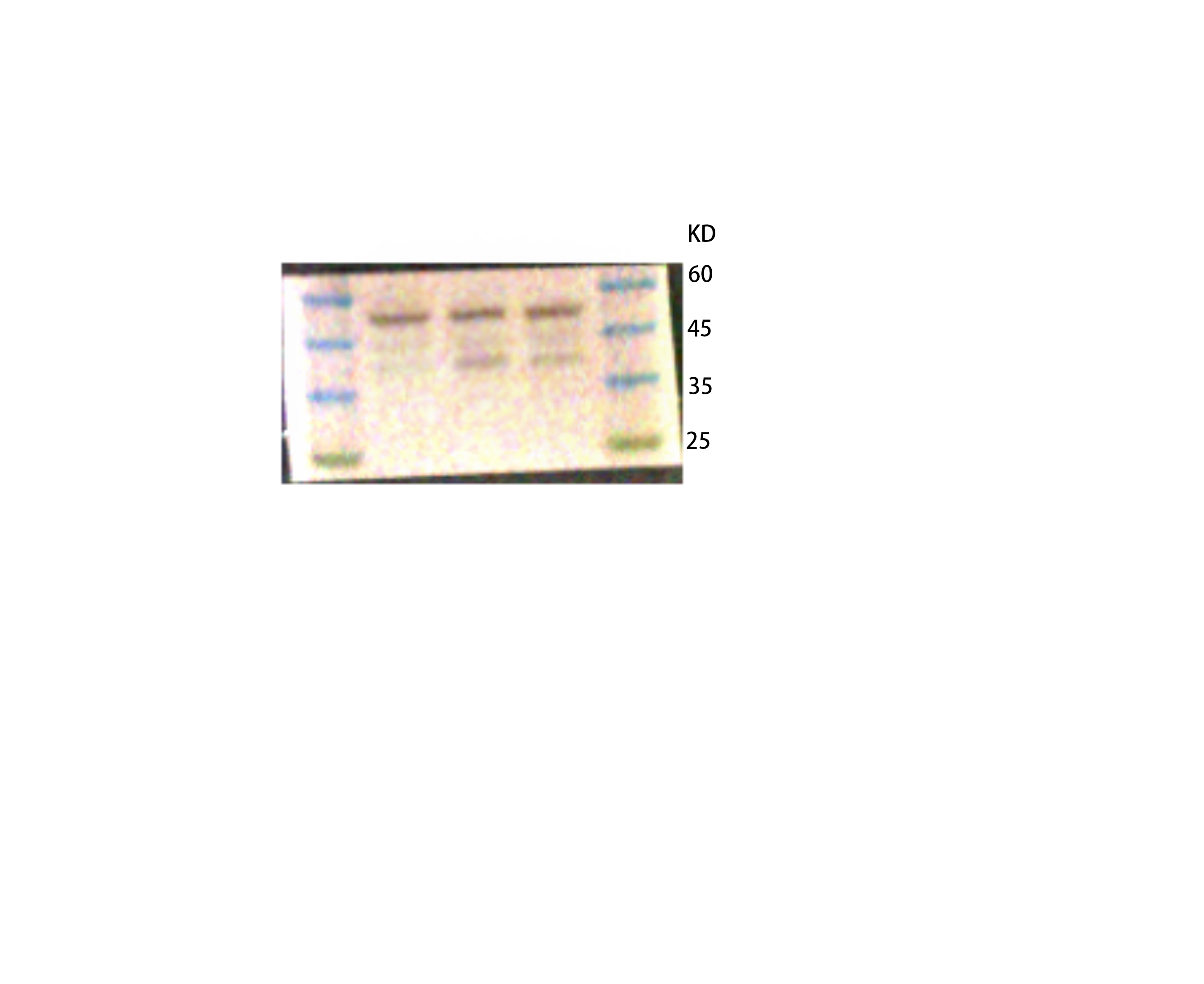

Supplement: Supplementary file 1 — Supplementary Material 1. [file 12964_2025_2550_MOESM1_ESM.zip › Sup_Figure 5G_tubulin+Marker.tif]

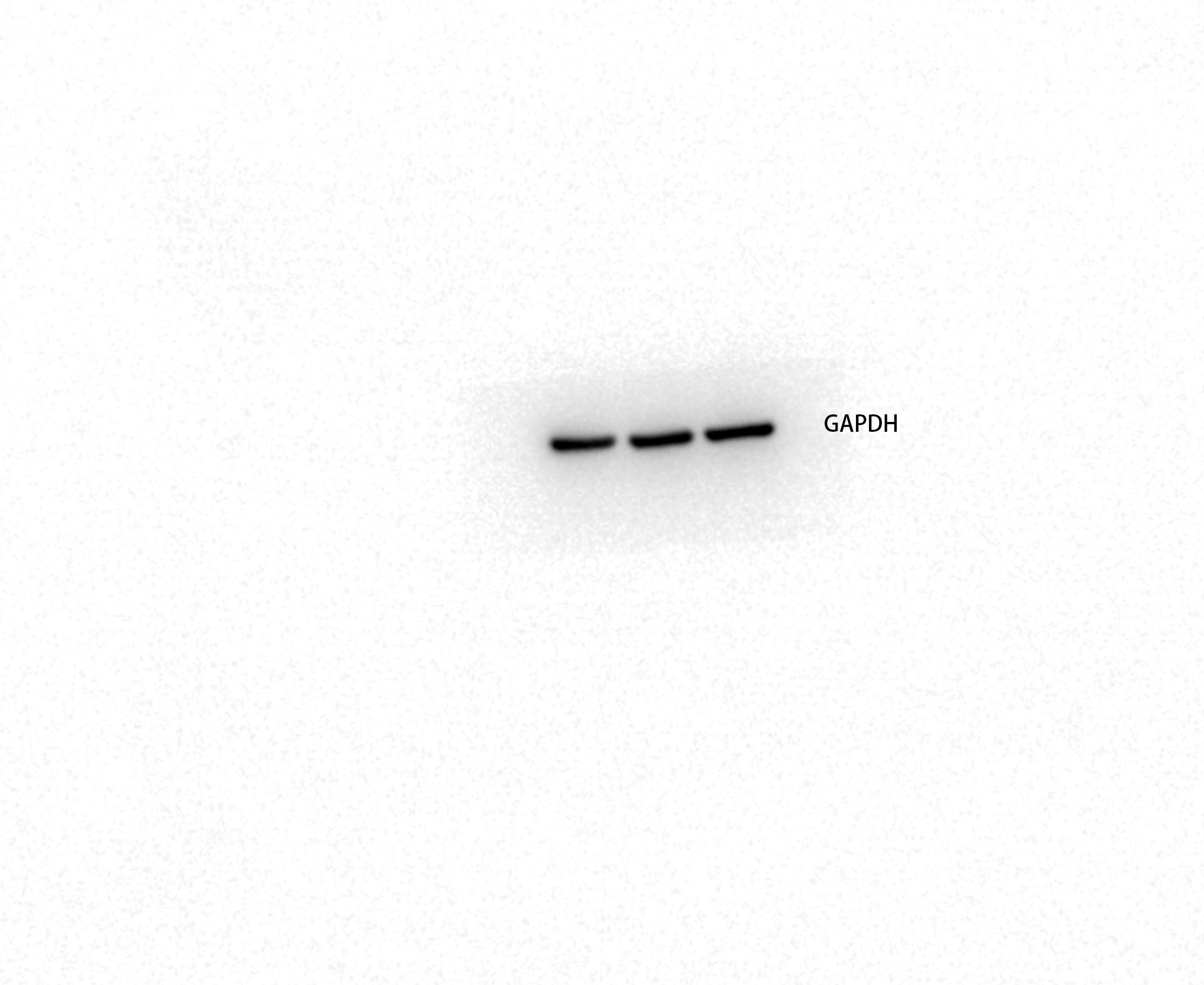

Supplement: Supplementary file 1 — Supplementary Material 1. [file 12964_2025_2550_MOESM1_ESM.zip › Sup_Figure6D_Fadu_GAPDH.tif]

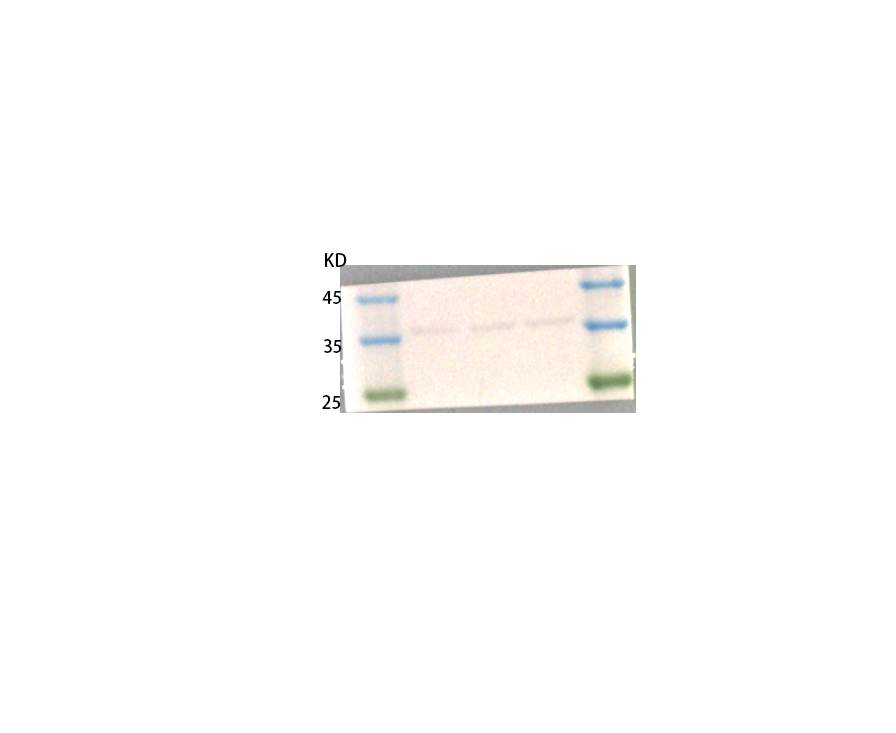

Supplement: Supplementary file 1 — Supplementary Material 1. [file 12964_2025_2550_MOESM1_ESM.zip › Sup_Figure6D_Fadu_GAPDH+Marker.tif]

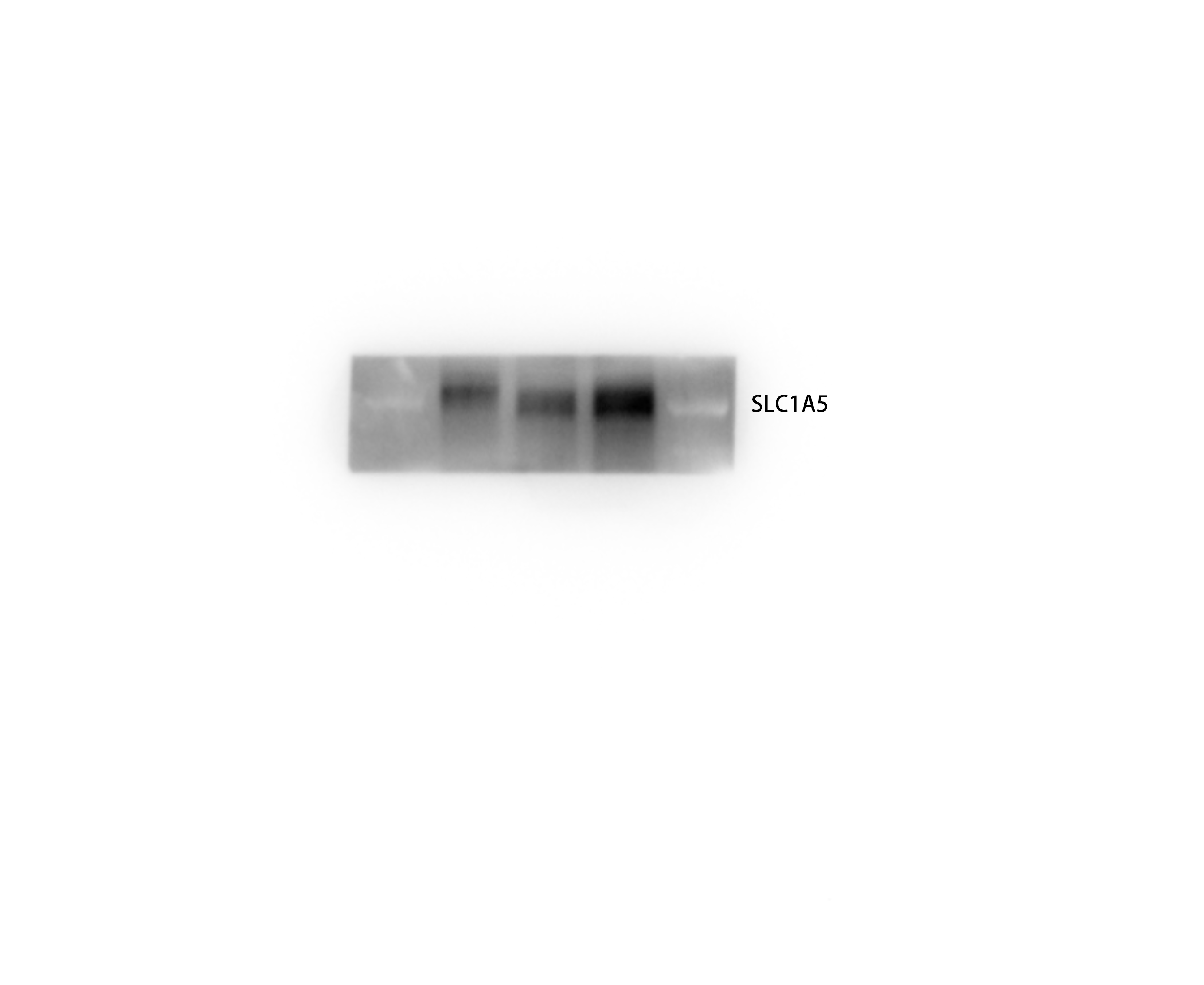

Supplement: Supplementary file 1 — Supplementary Material 1. [file 12964_2025_2550_MOESM1_ESM.zip › Sup_Figure6D_Fadu_SLC1A5.tif]

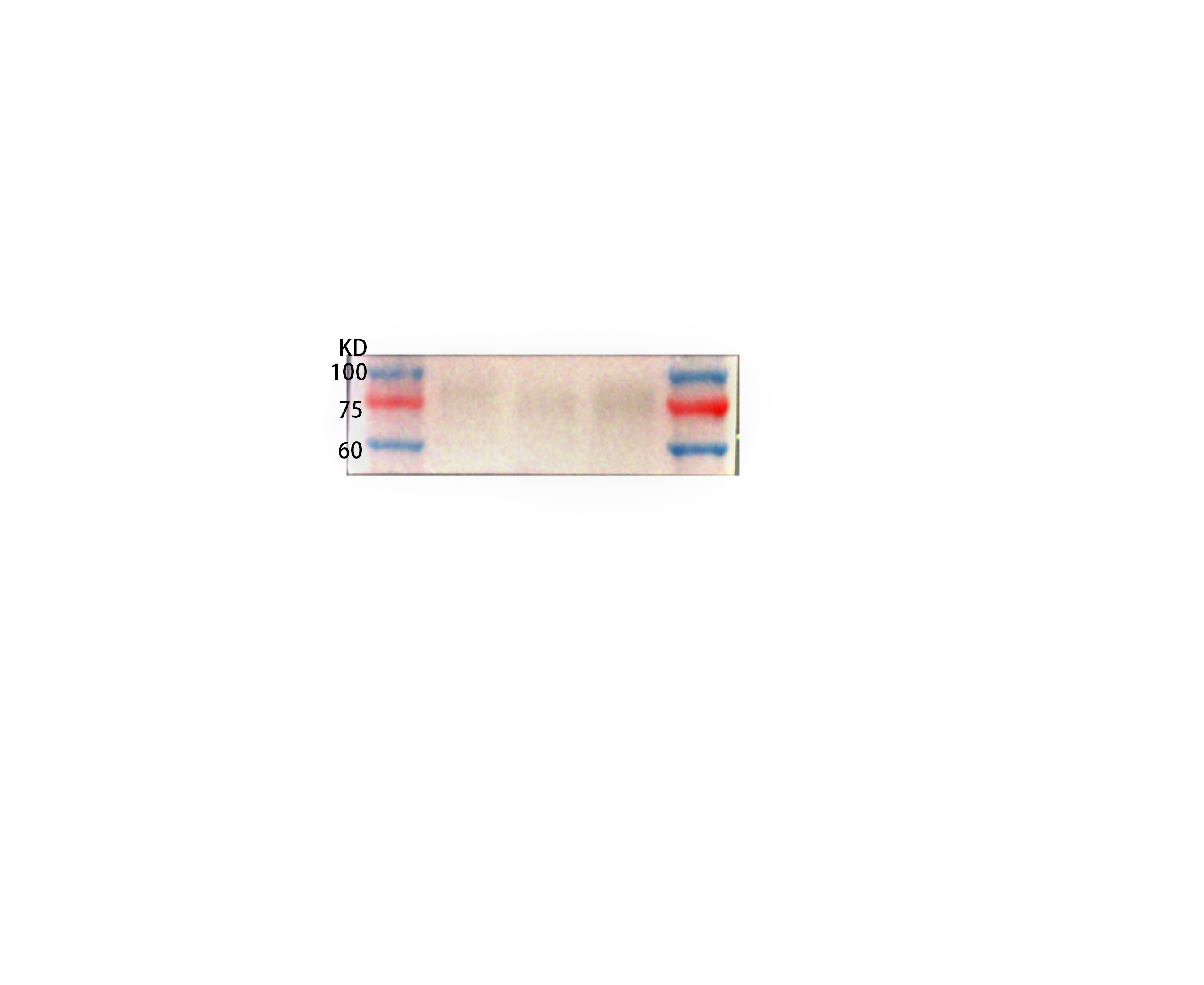

Supplement: Supplementary file 1 — Supplementary Material 1. [file 12964_2025_2550_MOESM1_ESM.zip › Sup_Figure6D_Fadu_SLC1A5+Marker.tif]

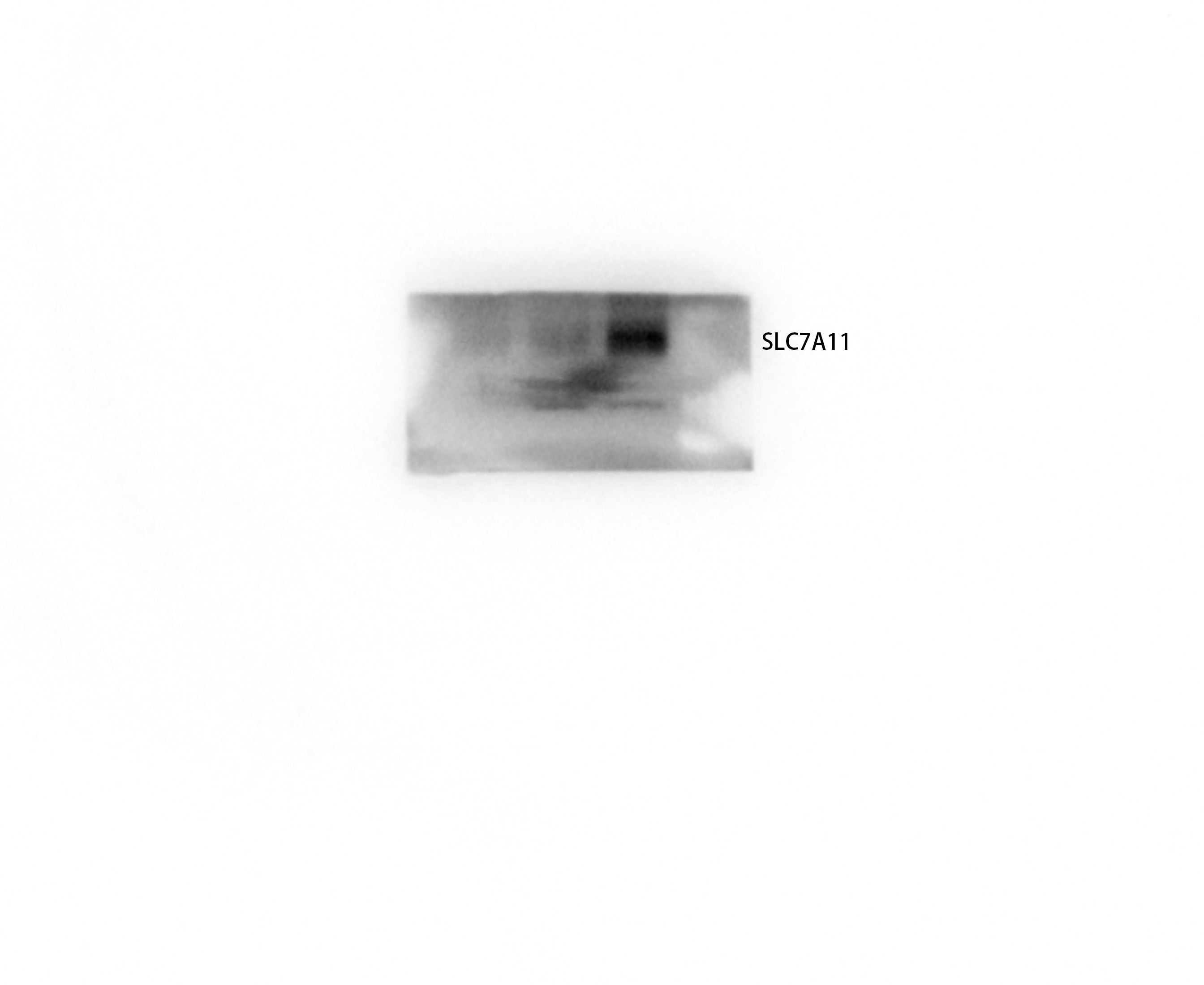

Supplement: Supplementary file 1 — Supplementary Material 1. [file 12964_2025_2550_MOESM1_ESM.zip › Sup_Figure6D_Fadu_SLC7A11.tif]

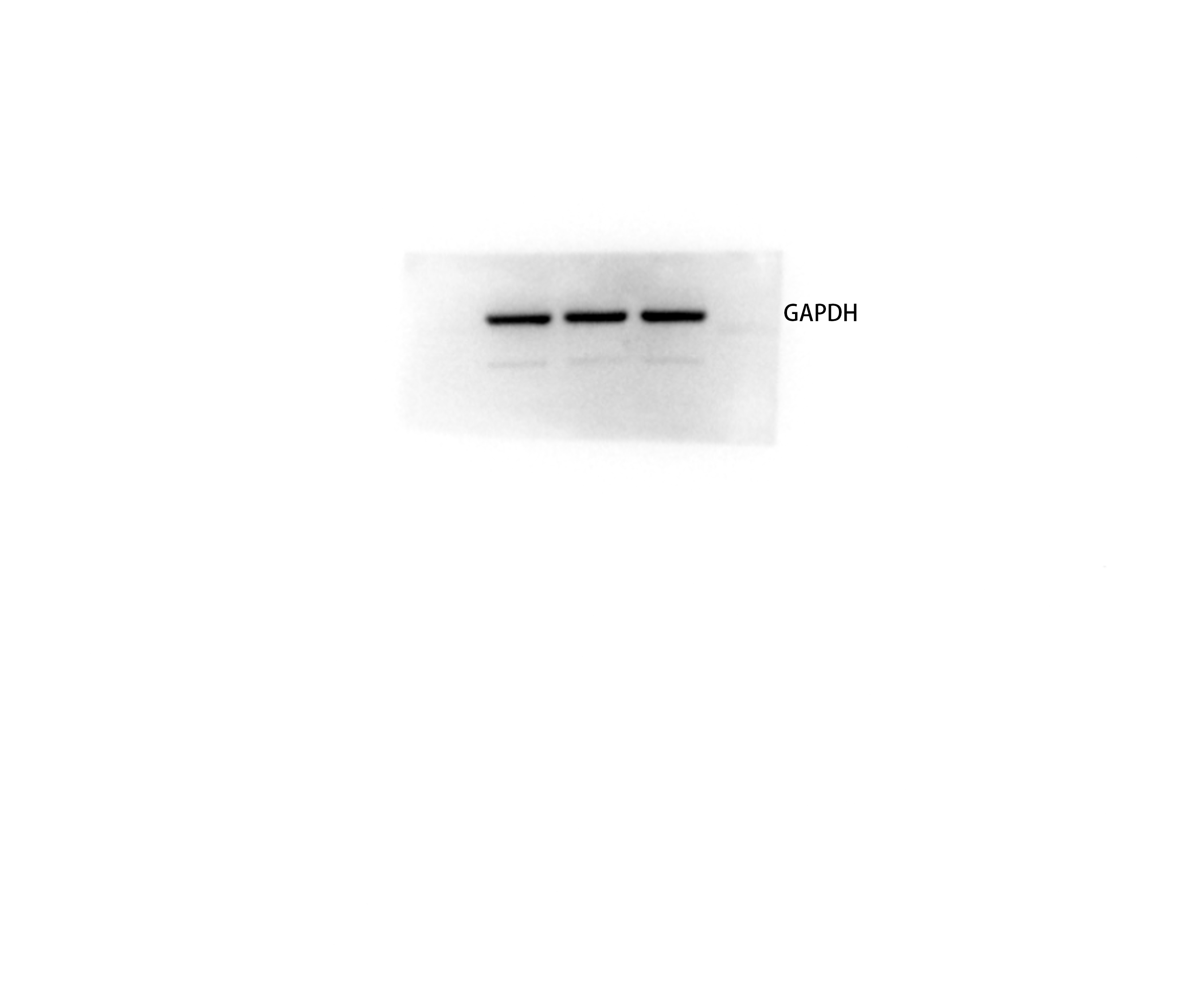

Supplement: Supplementary file 1 — Supplementary Material 1. [file 12964_2025_2550_MOESM1_ESM.zip › Sup_Figure6D_HN8_GAPDH.tif]

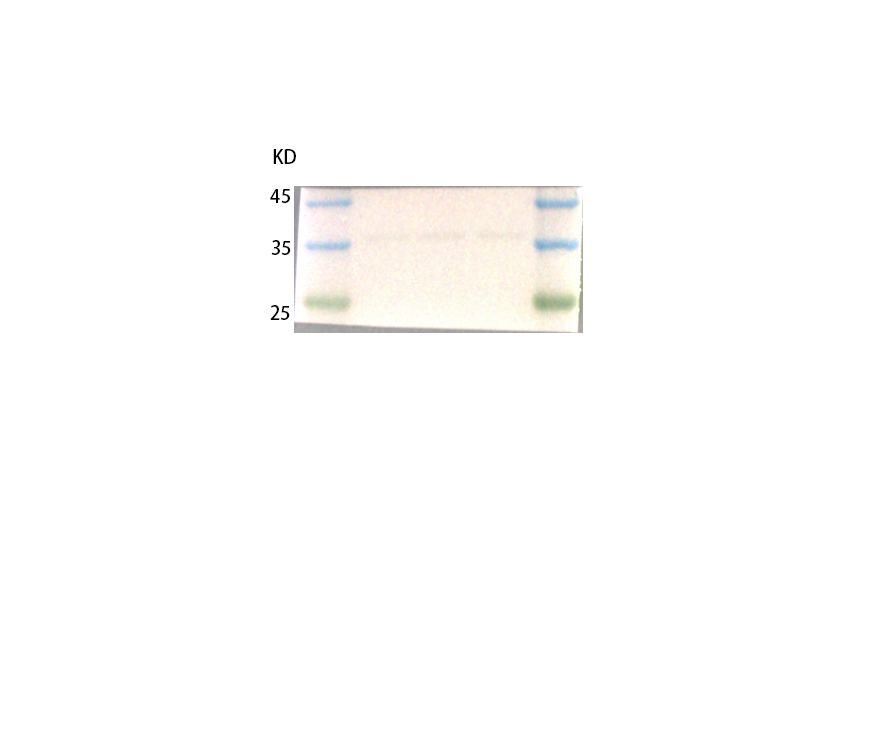

Supplement: Supplementary file 1 — Supplementary Material 1. [file 12964_2025_2550_MOESM1_ESM.zip › Sup_Figure6D_HN8_GAPDH+Marker.tif]

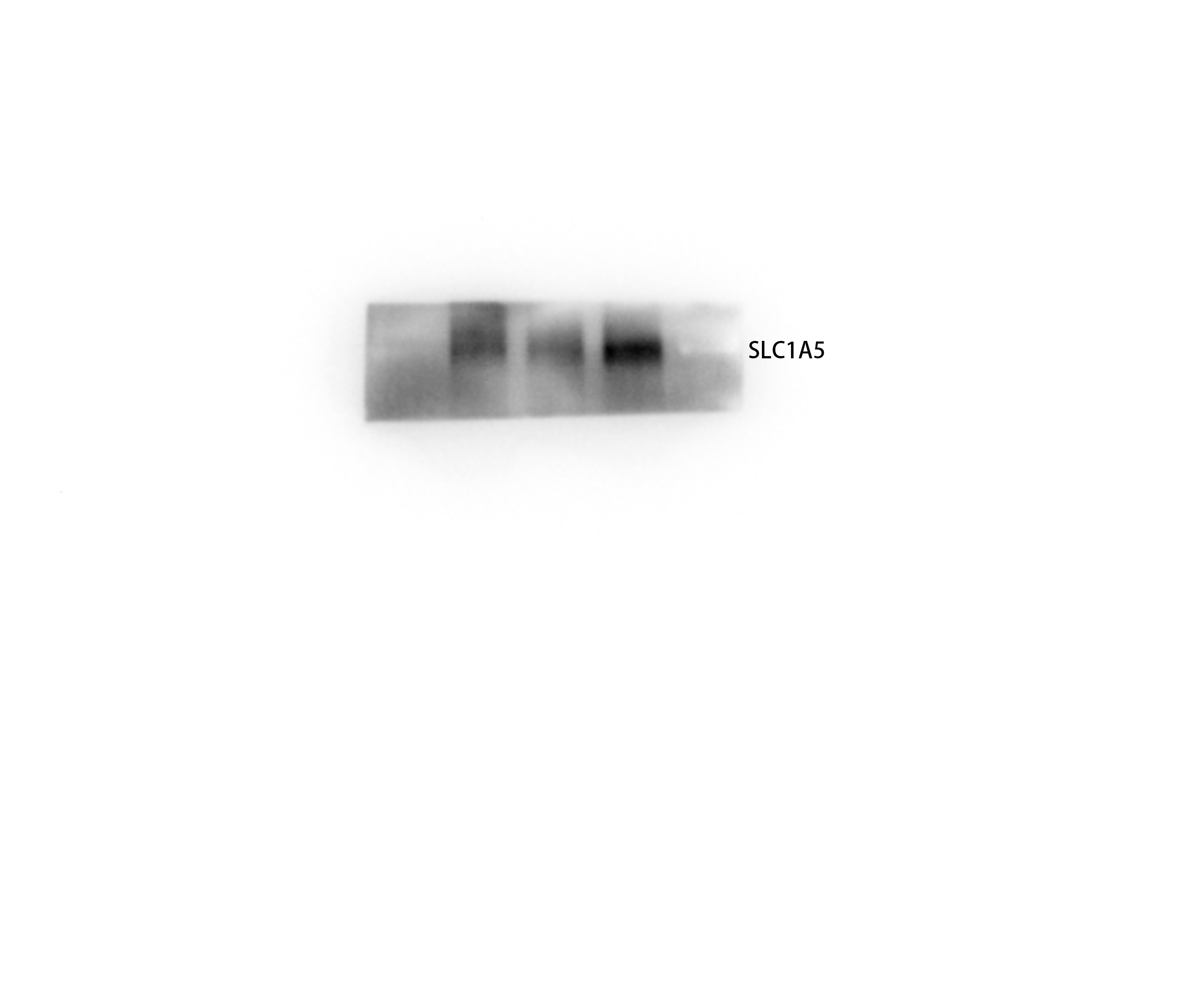

Supplement: Supplementary file 1 — Supplementary Material 1. [file 12964_2025_2550_MOESM1_ESM.zip › Sup_Figure6D_HN8_SLC1A5.tif]

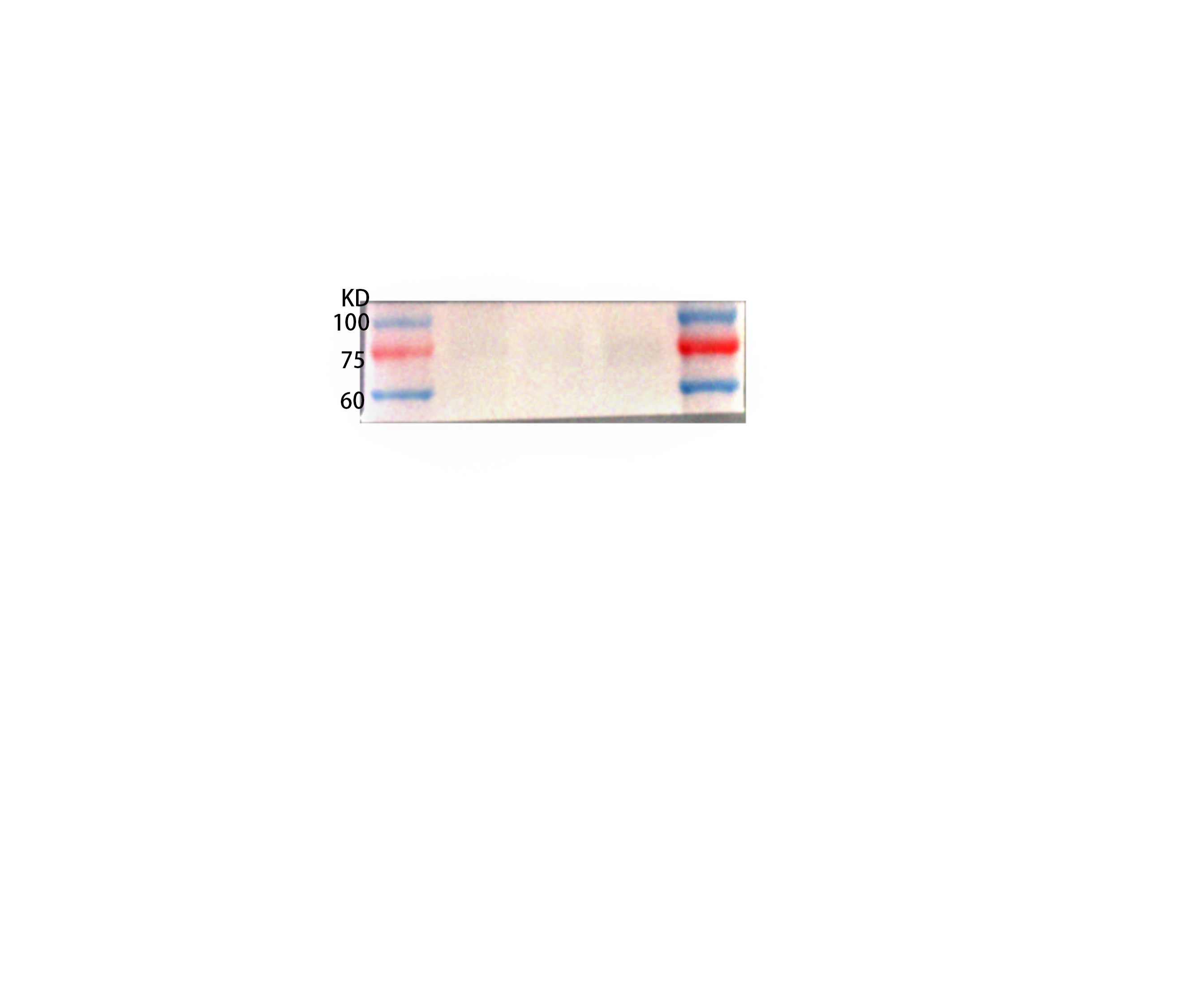

Supplement: Supplementary file 1 — Supplementary Material 1. [file 12964_2025_2550_MOESM1_ESM.zip › Sup_Figure6D_HN8_SLC1A5+Marker.tif]

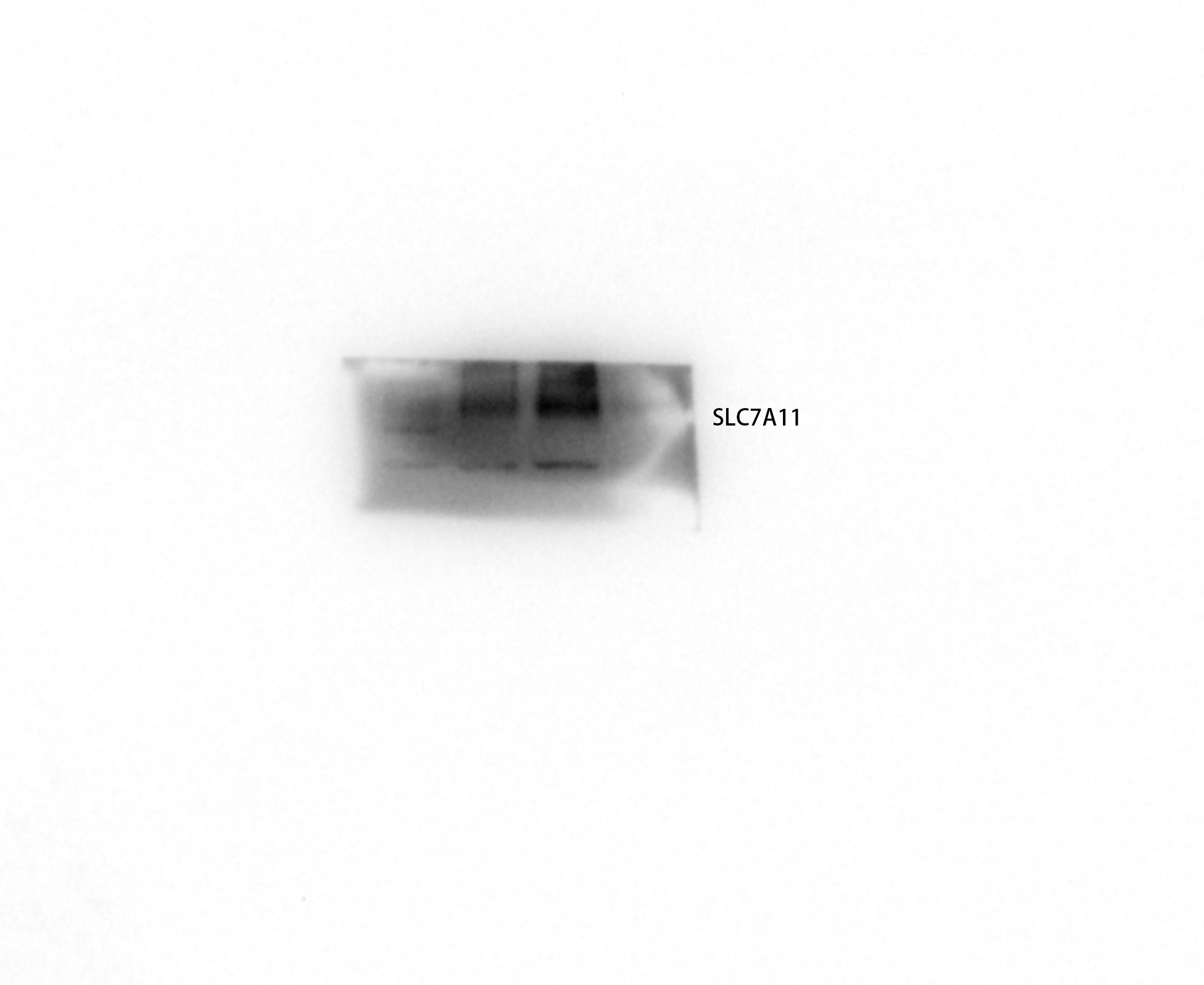

Supplement: Supplementary file 1 — Supplementary Material 1. [file 12964_2025_2550_MOESM1_ESM.zip › Sup_Figure6D_HN8_SLC7A11.tif]

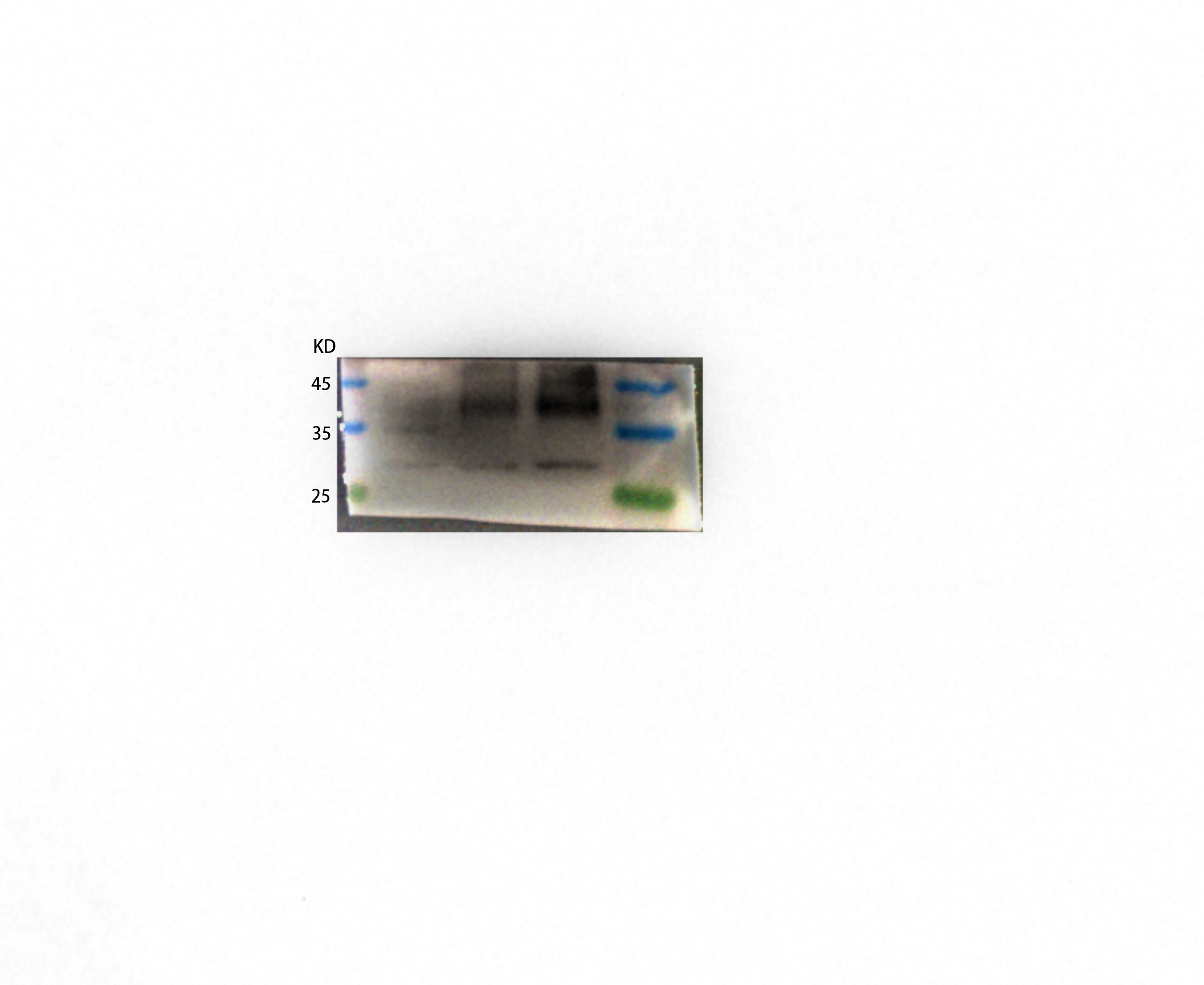

Supplement: Supplementary file 1 — Supplementary Material 1. [file 12964_2025_2550_MOESM1_ESM.zip › Sup_Figure6D_HN8_SLC7A11+Marker.tif]

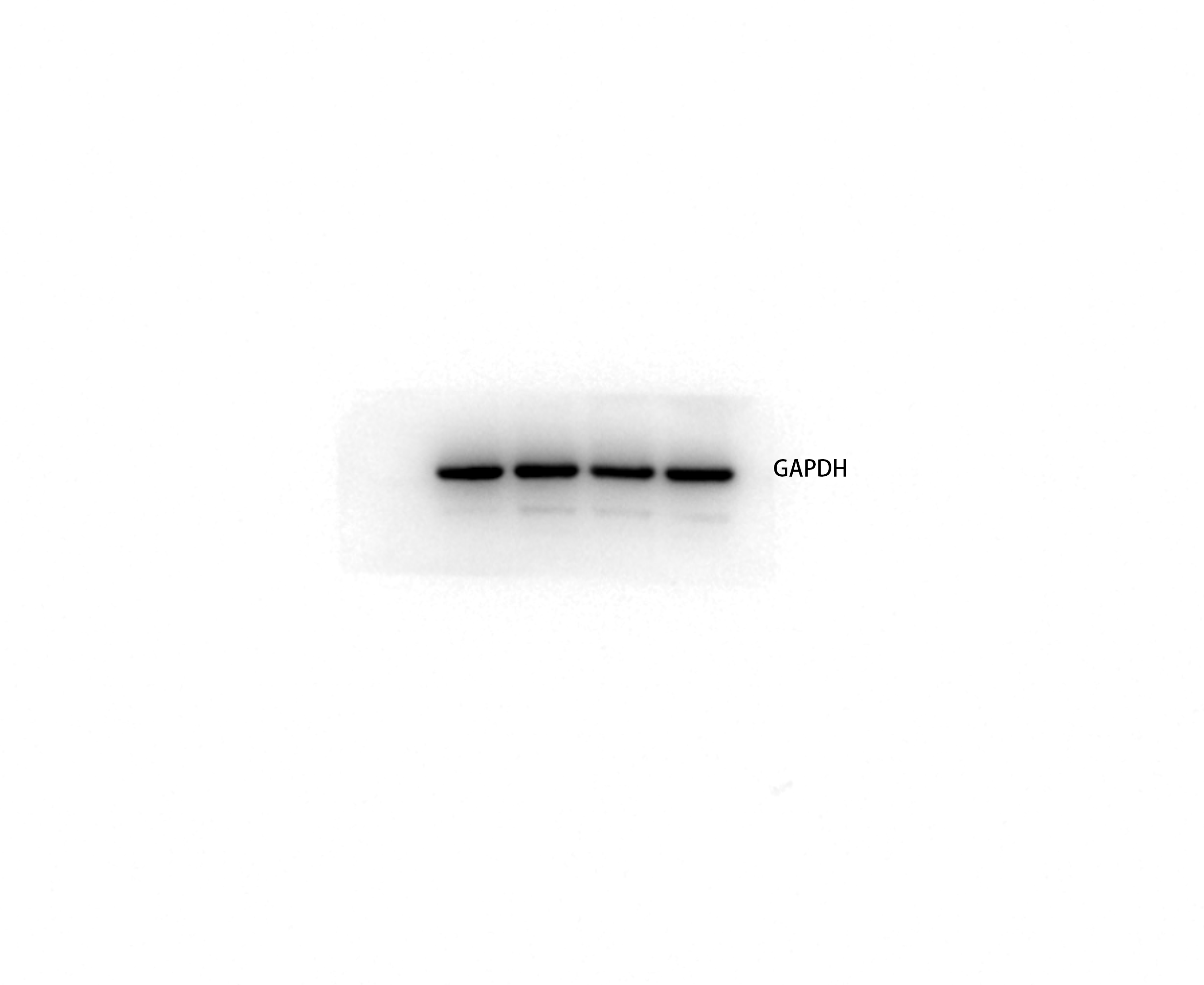

Supplement: Supplementary file 1 — Supplementary Material 1. [file 12964_2025_2550_MOESM1_ESM.zip › Sup_Figure6E_Fadu_GAPDH.tif]

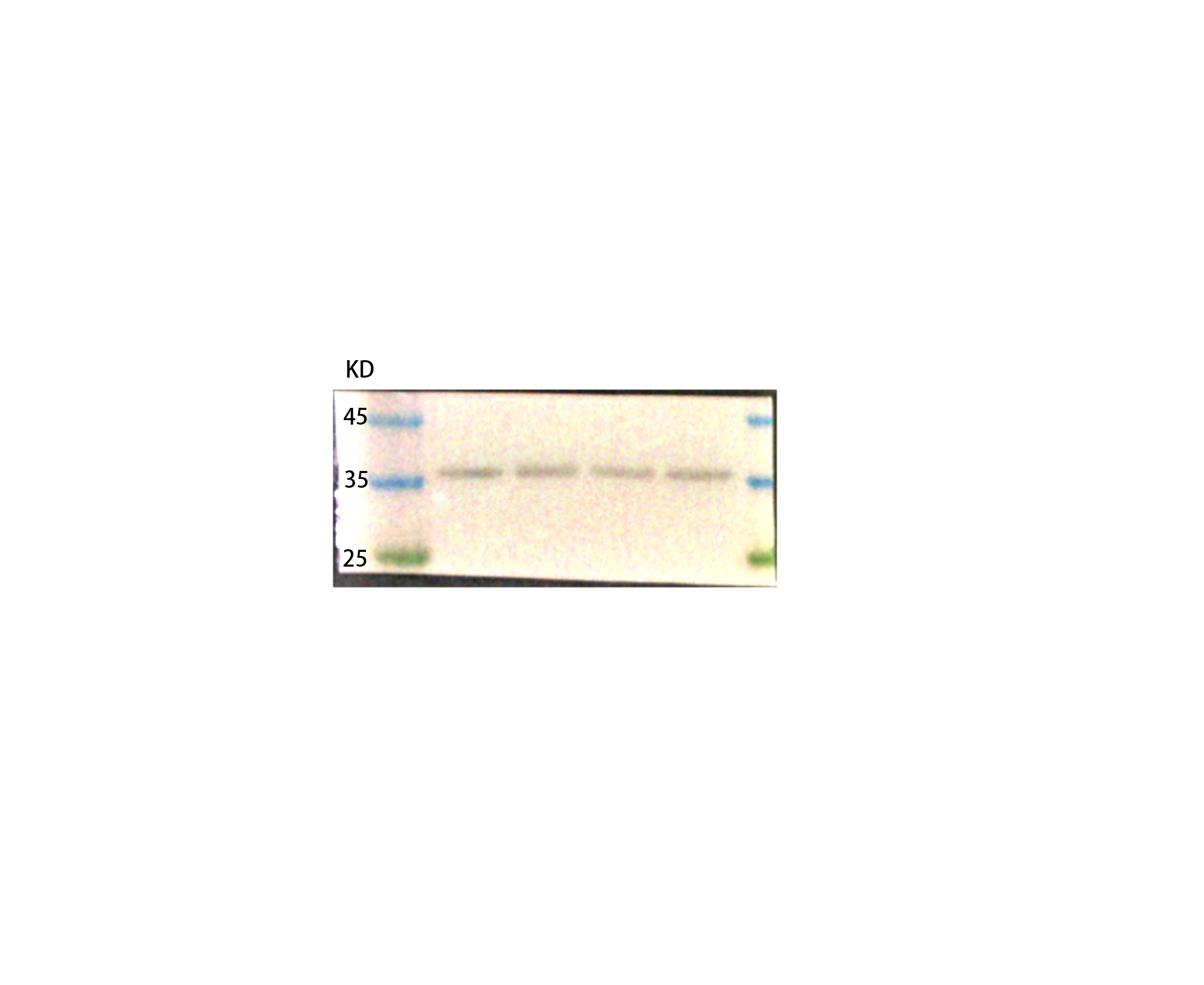

Supplement: Supplementary file 1 — Supplementary Material 1. [file 12964_2025_2550_MOESM1_ESM.zip › Sup_Figure6E_Fadu_GAPDH+Marker.tif]

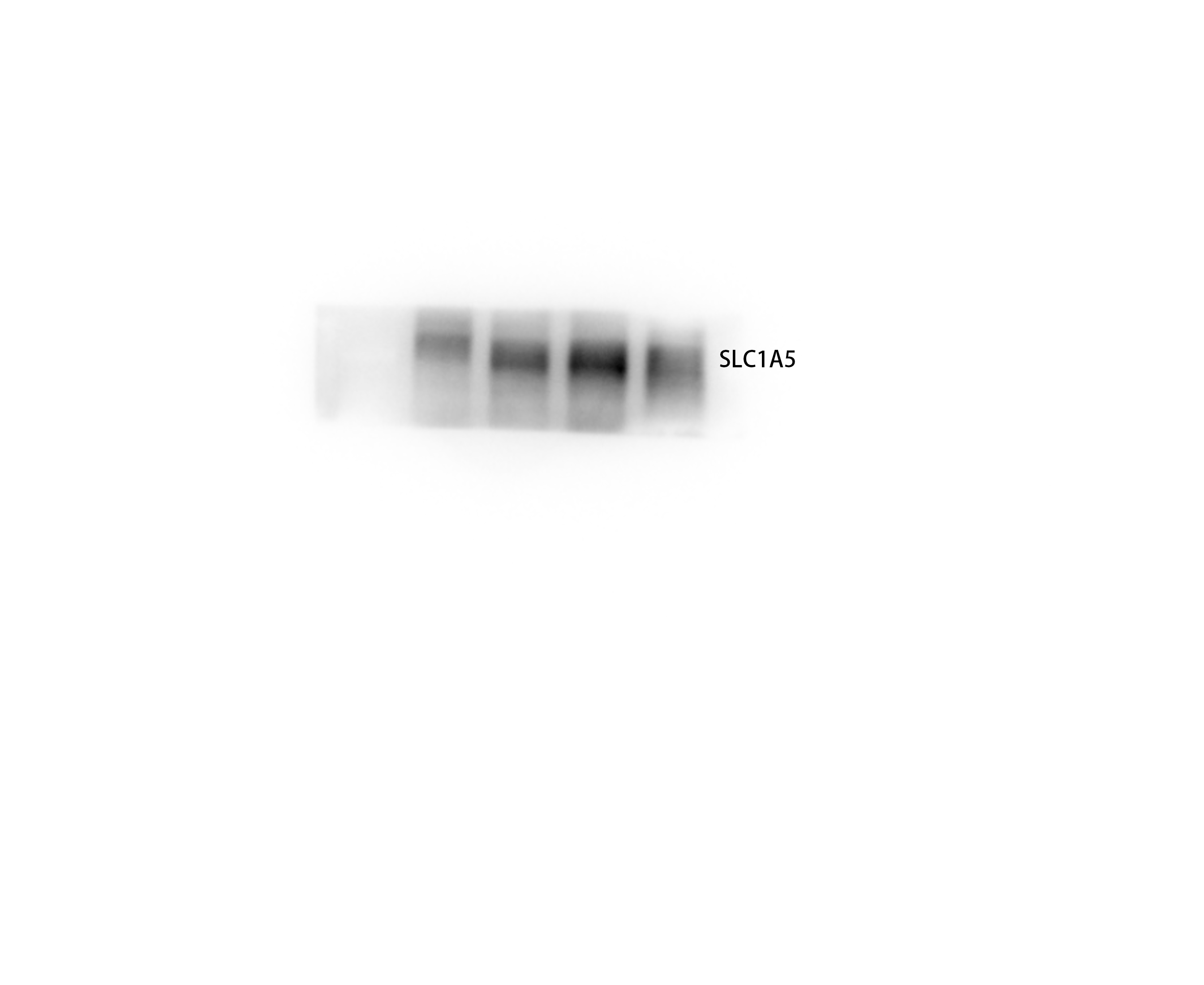

Supplement: Supplementary file 1 — Supplementary Material 1. [file 12964_2025_2550_MOESM1_ESM.zip › Sup_Figure6E_Fadu_SLC1A5.tif]

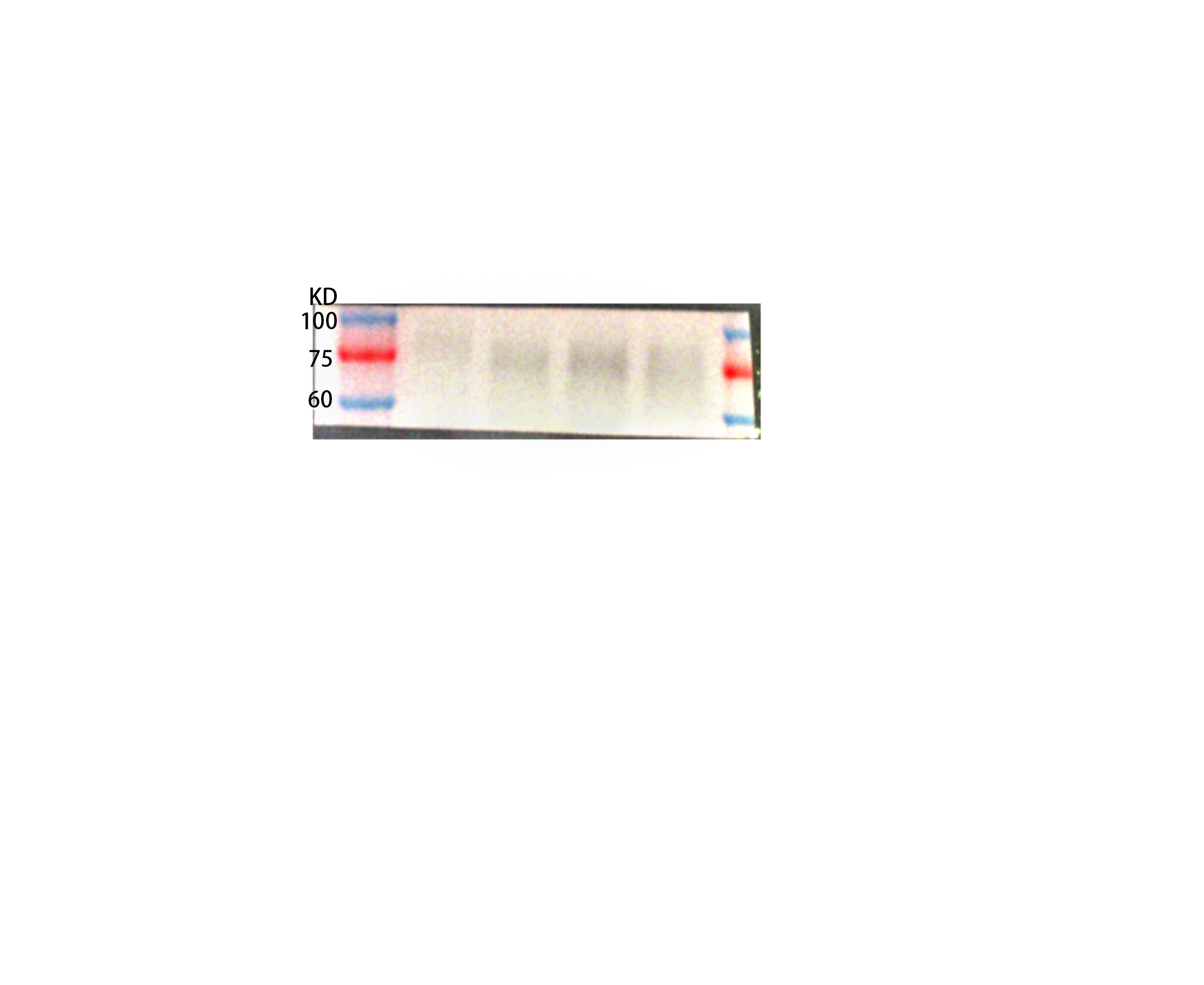

Supplement: Supplementary file 1 — Supplementary Material 1. [file 12964_2025_2550_MOESM1_ESM.zip › Sup_Figure6E_Fadu_SLC1A5+Marker.tif]

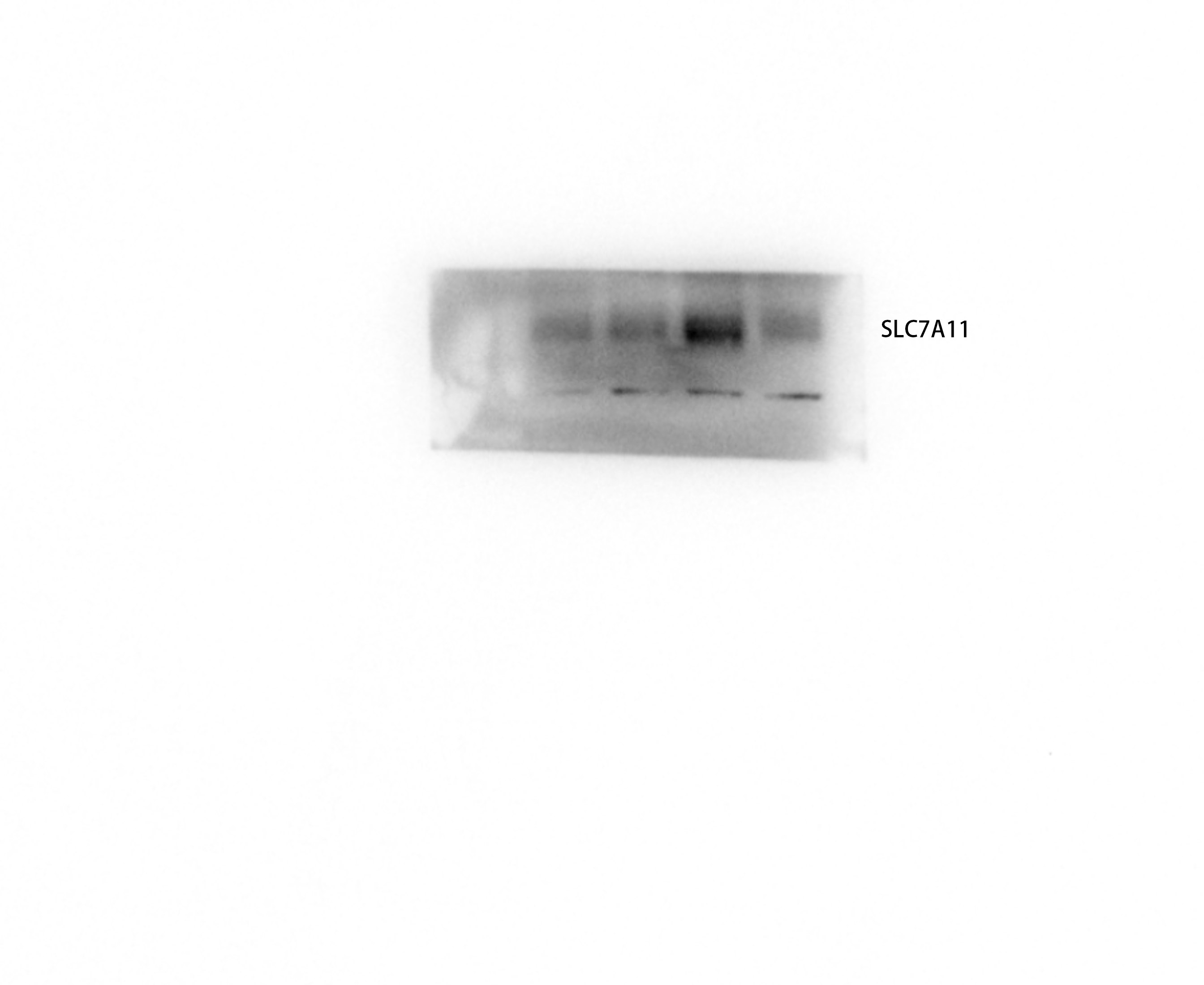

Supplement: Supplementary file 1 — Supplementary Material 1. [file 12964_2025_2550_MOESM1_ESM.zip › Sup_Figure6E_Fadu_SLC7A11.tif]

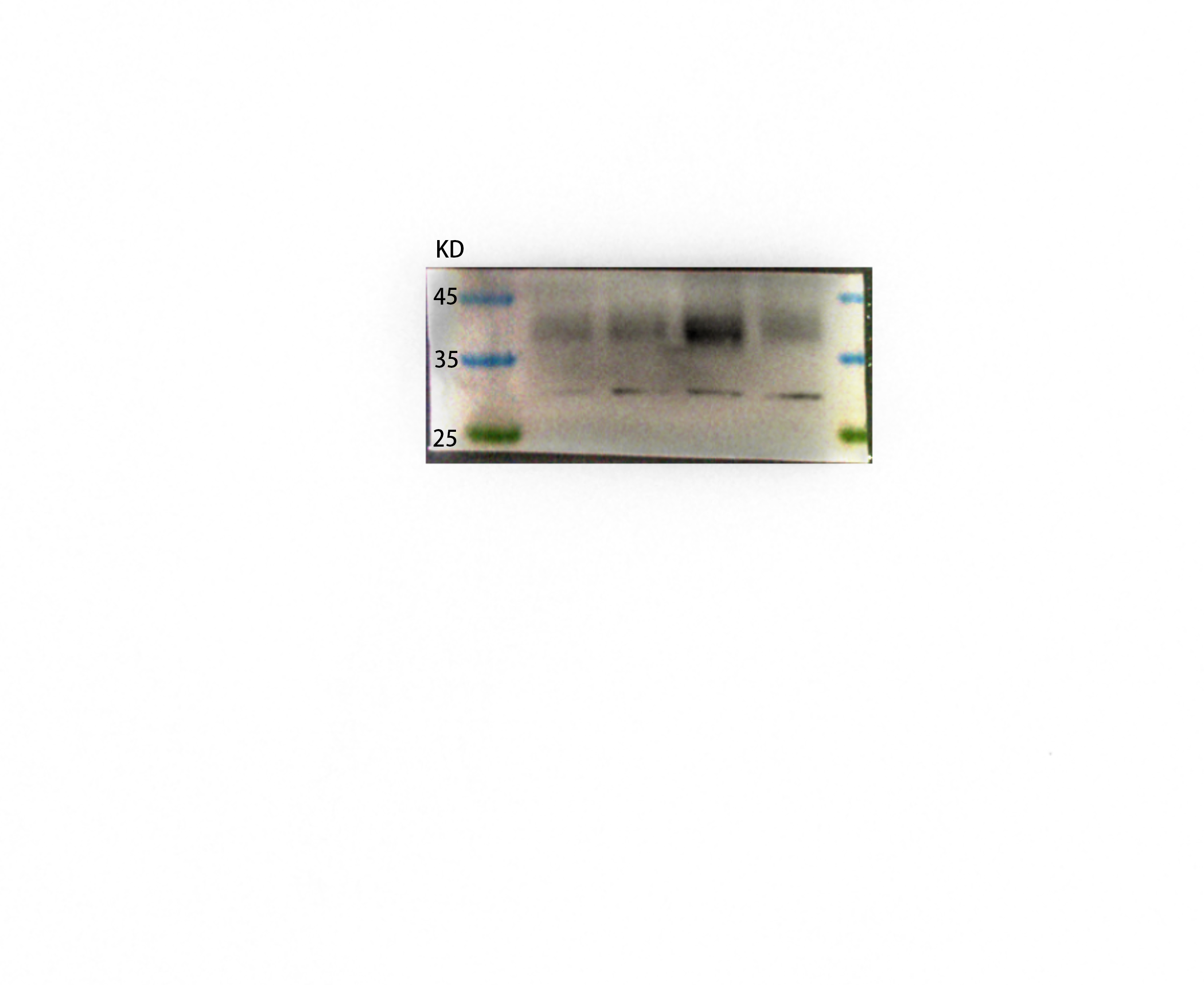

Supplement: Supplementary file 1 — Supplementary Material 1. [file 12964_2025_2550_MOESM1_ESM.zip › Sup_Figure6E_Fadu_SLC7A11+Marker.tif]
