# Supplementary material for: Fusobacterium nucleatum-reprogrammed adipocytes promote tumor cisplatin resistance through the CCL2-CCR2 axis in the necrotic metastatic neck nodes of head and neck carcinoma
Source: Cell Commun Signal. 2025 Nov 24;23:546. doi: 10.1186/s12964-025-02550-z (PMC12750780; doi:10.1186/s12964-025-02550-z)
Supplement: Supplementary file 2 — Supplementary Material 2: Supplemental Table 1. Basic characteristics of included patients. [file 12964_2025_2550_MOESM2_ESM.docx]

**Table 1 Basic characteristics of included patients.**

|  | Total  n = 24 |
| --- | --- |
| Age |  |
| >65 | 10 (42%) |
| Sex |  |
| male | 24 (100%) |
| Hypertension |  |
| Yes | 11 (45.8%) |
| Diabetes |  |
| Yes | 5 (20.8%) |
| Drinking |  |
| Yes | 17 (70.8%) |
| Smoking |  |
| Yes | 19 (79.2%) |
| Tumor site |  |
| hypopharyngeal | 16 (66.7%) |
| larynx | 8 (33.3%) |
| cT classification |  |
| 1~2 | 11 (45.8%) |
| 3~4 | 13 (54.2%) |
| cN classification |  |
| 1 | 5 (20.8%) |
| 2~3 | 19 (79.2%) |
| C stage |  |
| 3 | 4 (16.7%) |
| 4 | 20 (83.3%) |
