# Supplementary material for: Fusobacterium nucleatum-reprogrammed adipocytes promote tumor cisplatin resistance through the CCL2-CCR2 axis in the necrotic metastatic neck nodes of head and neck carcinoma
Source: Cell Commun Signal. 2025 Nov 24;23:546. doi: 10.1186/s12964-025-02550-z (PMC12750780; doi:10.1186/s12964-025-02550-z)
Supplement: Supplementary file 3 — Supplementary Material 3: Supplemental Table 2. DNA probs. [file 12964_2025_2550_MOESM3_ESM.docx]

| FUS664 Forward primer | CTTGTAGTTCCGC(C/T)TACCTC |
| --- | --- |
| EUB338 Forward primer | GCTGCCTCCCGTAGGAGT |

DNA probs
