# Supplementary material for: Fusobacterium nucleatum-reprogrammed adipocytes promote tumor cisplatin resistance through the CCL2-CCR2 axis in the necrotic metastatic neck nodes of head and neck carcinoma
Source: Cell Commun Signal. 2025 Nov 24;23:546. doi: 10.1186/s12964-025-02550-z (PMC12750780; doi:10.1186/s12964-025-02550-z)
Supplement: Supplementary file 4 — Supplementary Material 4: Supplemental Table 3. Details of antibodies. [file 12964_2025_2550_MOESM4_ESM.docx]

| Antibodies | Company | Catalog No. | Dilution ratio |
| --- | --- | --- | --- |
| Beclin-1 (D40C5) | Cell Signaling Technology, USA | # 3495 | 1:1000 for WB |
| SQSTM1/p62 (D6M5X) | Cell Signaling Technology, USA | # 23214 | 1:1000 for WB |
| LC3A/B (D3U4C) | Cell Signaling Technology, USA | # 12741 | 1:1000 for WB |
| LAMP2 (E6A6S) | Cell Signaling Technology, USA | # 34141 | 1:1000 for WB |
| HSL | Cell Signaling Technology, USA | # 4107 | 1:1000 for WB |
| Phospho-HSL (Ser660) | Cell Signaling Technology, USA | # 45804 | 1:1000 for WB |
| CREB (48H2) | Cell Signaling Technology, USA | # 9197 | 1:1000 for WB |
| Phospho-CREB (Ser133) (87G3) | Cell Signaling Technology, USA | # 9198 | 1:1000 for WB |
| SLC7A11 | Cell Signaling Technology, USA | # 12691 | 1:1000 for WB |
| SLC1A5/ASCT2 | Cell Signaling Technology, USA | # 8057 | 1:1000 for WB |
| β-Tubulin | Bioss, China | # BS-4511R | 1:100 for IF |
| β-Tubulin | Proteintech, China | # 66240-1-Ig | 1: 20000 for WB |
| Gapdh | Proteintech, China | # 60004-1-Ig | 1: 20000 for WB |
| Anti-mouse secondary antibody | Abmart, China | #M21001 | 1:5000 for WB |
| Anti-rabbit secondary antibody | Abcam, USA | # Ab6721 | 1:1000 for WB |
| Actin-Tracker | Beyotime, China | # C2205 | 1: 100 for IF |
| CCL2 | Proteintech, China | #26161-1-AP | 1: 100 for IF |

**Details of antibodies**
