# Supplementary material for: Fusobacterium nucleatum-reprogrammed adipocytes promote tumor cisplatin resistance through the CCL2-CCR2 axis in the necrotic metastatic neck nodes of head and neck carcinoma
Source: Cell Commun Signal. 2025 Nov 24;23:546. doi: 10.1186/s12964-025-02550-z (PMC12750780; doi:10.1186/s12964-025-02550-z)
Supplement: Supplementary file 5 — Supplementary Material 5: Supplemental Table 4. Sequences of primers and probes. [file 12964_2025_2550_MOESM5_ESM.docx]

**Sequences of primers and probes**

Mouse

| Gene name | Sequence |
| --- | --- |
| MMP11-F | GGTTTCCACCATCCGAGGAG |
| MMP11-R | CAAAAGCTGCATCCACAGGG |
| Leptin-F | GGGATGGGTAGAGCCTTTGG |
| Leptin-R | AAGTGCTTCCATCGTGTGCT |
| IL6-F | CTTCTTGGGACTGATGCTGGT |
| IL6-1B | GTTGGGAGTGGTATCCTCTGTG |
| IL1B-F | AATCTCGCAGCAGCACATCA |
| IL1B-R | GAAGGTCCACGGGAAAGACA |
| CCL5-F | TTCCCTGTCATTGCTTGCTCT |
| CCL5-R | CCATTTTCCCAGGACCGAGT |
| CCL2-F | TGACCCCAAGAAGGAATGGG |
| CCL2-R | ACCTTAGGGCAGATGCAGTT |

Human

| Gene name | Sequence |
| --- | --- |
| SLC1A5-F | TTCACCCGCAAAAACCCCTA |
| SLC1A5-R | GGCCACGCCATTATTCTCCT |
| SLC7A11-F | TGTGTGGGGTCCTGTCACTA |
| SLC7A11-R | TGAGGAGTTCCACCCAGACT |
| GLS-F | ACAGGGTCTGTTACCTAGCTT |
| GLS-R | TGGGATCAGACGTTCGCAAT |
| GOT1-F | GAAGAAACACCACCCGTCCA |
| GOT1-R | CTCACCAGAGCAGCCTTTCA |
| GLUD1-F | GTTTGGGGGTGCTAAAGCTG |
| GLUD1-R | CATGTCTGGAGCAGGCACAT |
| GCLC-F | AGGTGACATTCCAAGCCTGC |
| GCLC-R | CACTCCCCAGCGACAATCAA |
| GSTP1-F | GCGGGCAAGGATGACTATGT |
| GSTP1-R | CAGCAGGTTGTAGTCAGCGA |

*F. nucleatum*

| Gene name | Sequence |
| --- | --- |
| *F. nucleatum* -F | CAACCATTACTTTAACTCTACCATGTTCA |
| *F. nucleatum* -R | GTTGACTTTACAGAAGGAGATTATGTAAAAATC |
| PGT-F | ATCCCCAAAGCACCTGGTTT |
| PGT-R | AGAGGCCAAGATAGTCCTGGTAA |
